# Computational studies of Brønsted acid-catalyzed transannular cycloadditions of cycloalkenone hydrazones

**DOI:** 10.3762/bjoc.19.37

**Published:** 2023-04-20

**Authors:** Manuel Pedrón, Jana Sendra, Irene Ginés, Tomás Tejero, Jose L Vicario, Pedro Merino

**Affiliations:** 1 Instituto de Biocomputación y Física de Sistemas Complejos (BIFI), Universidad de Zaragoza, 50009 Zaragoza, Spain; 2 Departamento de Química Orgánica e Inorgánica, Universidad del País Vasco (UPV/EHU) P.O. Box 644, 48080 Bilbao, Spain; 3 Instituto de Síntesis Química y Catálisis Homogénea (SQCH), Universidad de Zaragoza-CSIC, 50009 Zaragoza, Spain

**Keywords:** DFT, distortion model, hydrazones, transannular cycloadditions

## Abstract

The contribution to the energy barrier of a series of tethers in transannular cycloadditions of cycloalkenes with hydrazones has been computationally studied by using DFT. The Houk's distortion model has been employed to evaluate the influence of the tether in the cycloaddition reaction. That model has been extended to determine the contribution of each tether and, more importantly, the effect exerted between them. In addition to the distortion induced by the tethers, the entropy effects caused by them has also been studied. The analysis of the evolution of the electron localization function along the reaction revealed the highly concerted character of the reaction.

## Introduction

Transannular cycloaddition reactions (TCRs) are useful for the synthesis of complex natural products and other biologically active compounds with high efficiency and stereoselectivity [1–4]. There are several different ways in which TCRs can occur, depending on the nature of the starting materials and the conditions used [5]. Some common types of TCRs include Diels–Alder reactions [6–20], photocycloadditions [21–28], and other types of multistep cycloadditions [29].

Steric hindrance can have a significant effect on the outcome of these reactions. The tether(s) connecting the reactive functional groups affects the spatial orientation of the reacting species and introduces strain into the starting cyclic molecule. As a consequence, the reaction barrier is highly dependent on distortion and entropic effects as Houk and co-workers demonstrated for transannular Diels–Alder cycloaddition reactions of symmetrically tethered large systems (10–18-membered rings) [29].

**Scheme 1:**
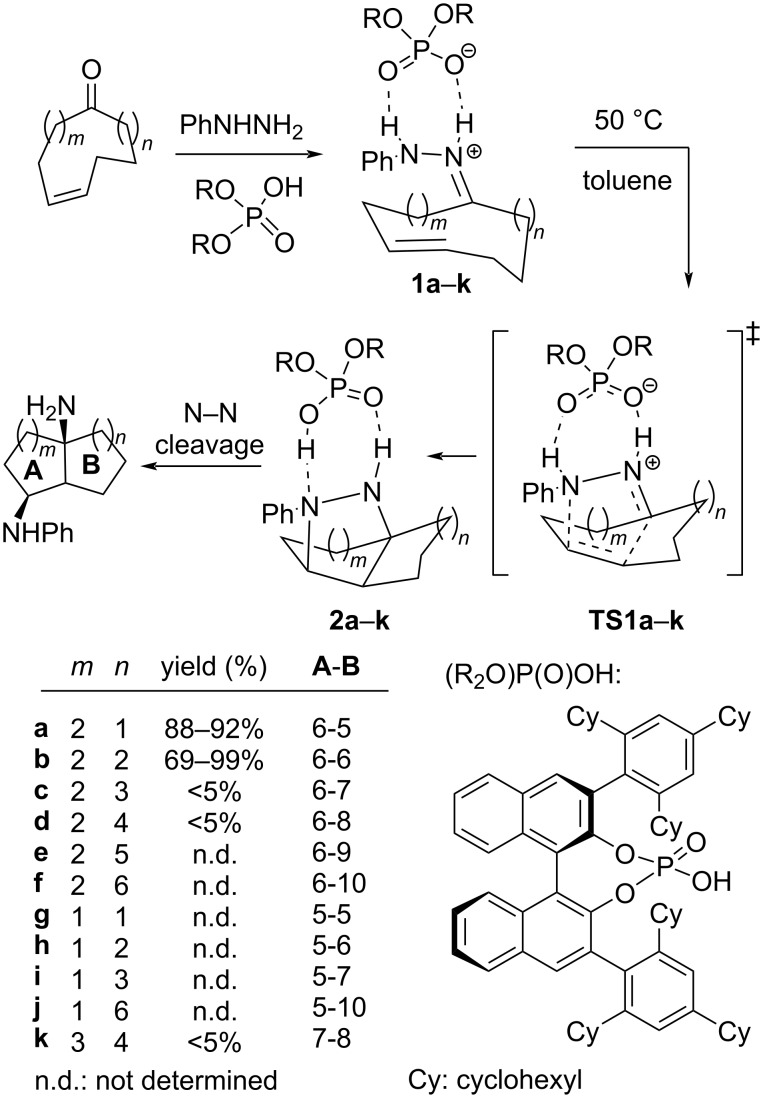
Experimental data (series **a**–**d**, **k**) and non-studied examples (series **e**–**j**) for transannular cycloadditions of cycloalkenone hydrazones.

In this context, we have recently reported the transannular enantioselective (3 + 2) cycloaddition of cycloalkenone hydrazones under Brønsted acid catalysis in route to enantiomerically pure bicyclic 1,3-diamines (Scheme 1) [29]. The reaction led to excellent results when decalines and octahydro-1*H*-indene bicyclic scaffolds were formed (series **a** and **b**) but failed in other cases (series **c**, **d**, and **k**). Series **e**, **f**, **g**, **h**, and **i** have not been tested experimentally.

In this work, we present our results on the computational study of the transannular reaction illustrated in Scheme 1 for several nonsymmetric tether combinations between the hydrazone and double bond moieties leading to a sort of condensed cyclohexanes (series **a**–**f**) and other bicyclic systems (series **g**–**k**) with the aim of explaining the observed lack of reactivity and predicting new reactive substrates. For that purpose, we applied the Houk's distortion model [30] to nonsymmetrically tethered systems and extended the model to estimate the mutual influence of the two different tethers. We have also carried out an analysis of the electron localization function (ELF) [31–32] and the charge transfer along the reaction coordinate to determine the different stages and the polarity of the reaction.

## Results and Discussion

The enantioselective intermolecular cycloaddition between hydrazones and alkenes under chiral BINOL-derived Brønsted acid catalysis has been studied by Houk and Rueping in 2014 [33]. These authors established the origin of the enantioselectivity and the differences between the catalyzed and uncatalyzed reactions, suggesting that the catalyzed reaction is, actually, a so-called (3^+^ + 2) reaction in which distortion effects are crucial for achieving the required ion-pair geometry in the transition state. Following this precedent, we proceeded to calculate the energy barriers and the corresponding activation parameters for all the reactions illustrated in Scheme 1 (series **a**–**k**), which are listed in Table 1. We used the phosphoric acid derived from 2,2'-biphenol as a model for the catalyst.

**Table 1:** Calculated activation parameters for transannular cyclizations illustrated in Scheme 1.^a^

reactant	product	system	Δ*E*(0)^≠^	Δ*H*^≠^	−*T*·Δ*S*^≠^	Δ*G*^≠^

**1a**	**2a**	6-5	21.3	20.6	2.1	23.2
**1b**	**2b**	6-6	20.5	19.7	2.1	22.4
**1c**	**2c**	6-7	27.2	26.4	1.9	28.8
**1d**	**2d**	6-8	32.2	31.5	1.8	33.7
**1e**	**2e**	6-9	30.3	28.9	6.1	36.6
**1f**	**2f**	6-10	35.5	34.5	2.7	37.8
**1g**	**2g**	5-5	23.0	22.3	1.4	24.0
**1h**	**2h**	5-6	21.3	20.4	2.8	23.9
**1i**	**2i**	5-7	30.8	30.1	1.6	32.1
**1j**	**2j**	5-10	35.6	34.4	2.7	37.8
**1k**	**2k**	7-8	33.7	32.5	4.1	37.6

^a^Level: m062x/6-311+G(d,p)/SMD=toluene//m062x/6-31G(d).

**Figure 1:**
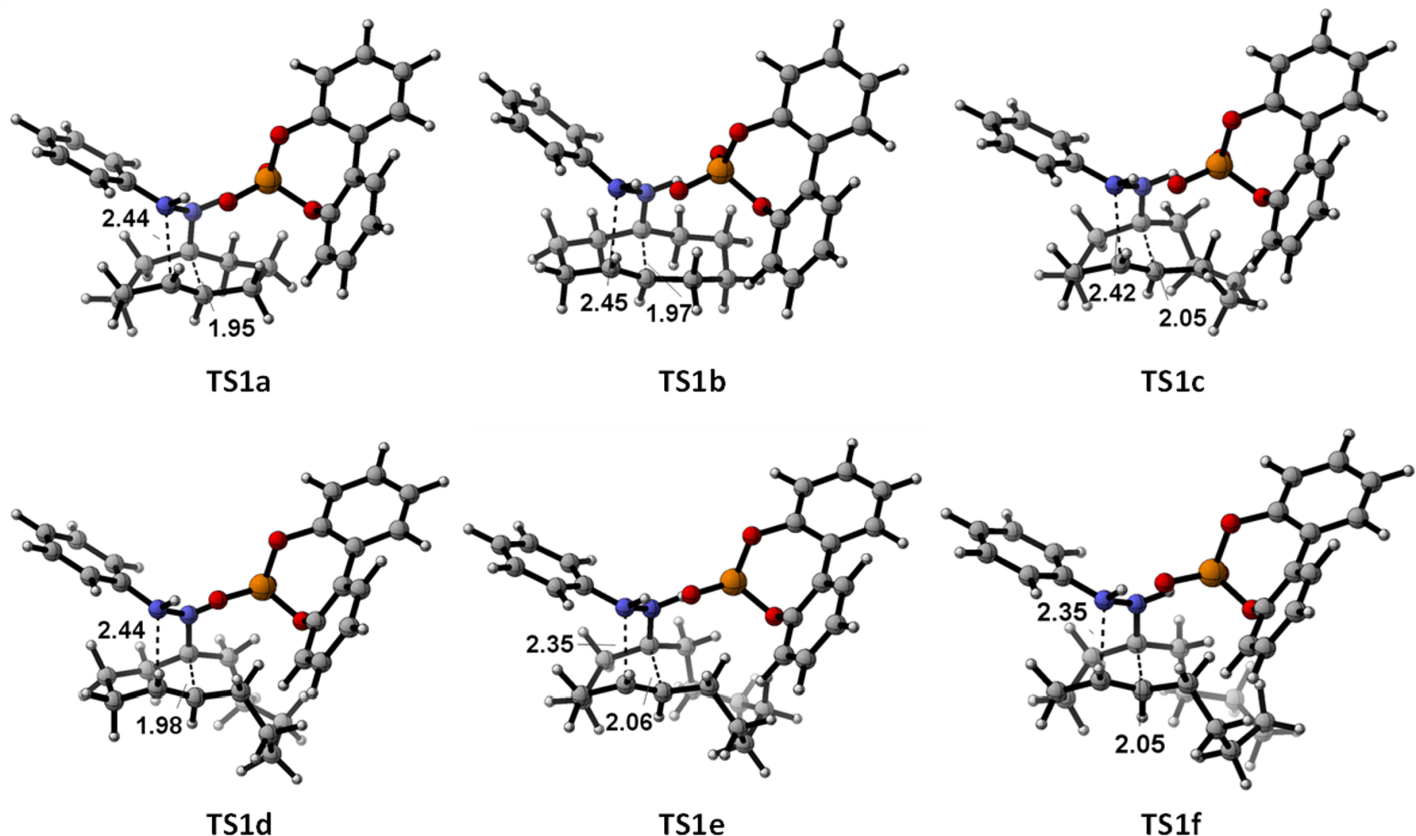
Optimized (m062x/6-31G(d)) geometries for the transition structures of series **a**–**f**.

The optimized geometries of the corresponding transition structures are given in Figure 1 (only those corresponding to fused cyclohexanes are shown, for the rest see Supporting Information File 1).

Only the reactions corresponding to the reaction of **1a** and **1b** show barriers close to 20 kcal/mol thus being plausible to work at ambient temperature or under some heating, which is consistent with the fact that the formation of these adducts were experimentally observed to happen with good yields. Similarly, data of Table 1 predict that the reaction of **1g** and **1h** leading to 5-5 and 5-6 systems (not tested experimentally, yet), respectively could also be observed experimentally. On the other hand, the higher activation barrier of compounds **1c**, **1d**, and **1k** makes the cyclization way more energy demanding, which is fully consistent with the experimental results, where no reaction could be observed. In all these cases, the starting hydrazones were recovered unchanged.

For the other systems (**1e**, **1f**, and **1j**), we predict no reaction at all, since, as before, the energy barriers become unreachable at common reaction conditions.

**Figure 2:**
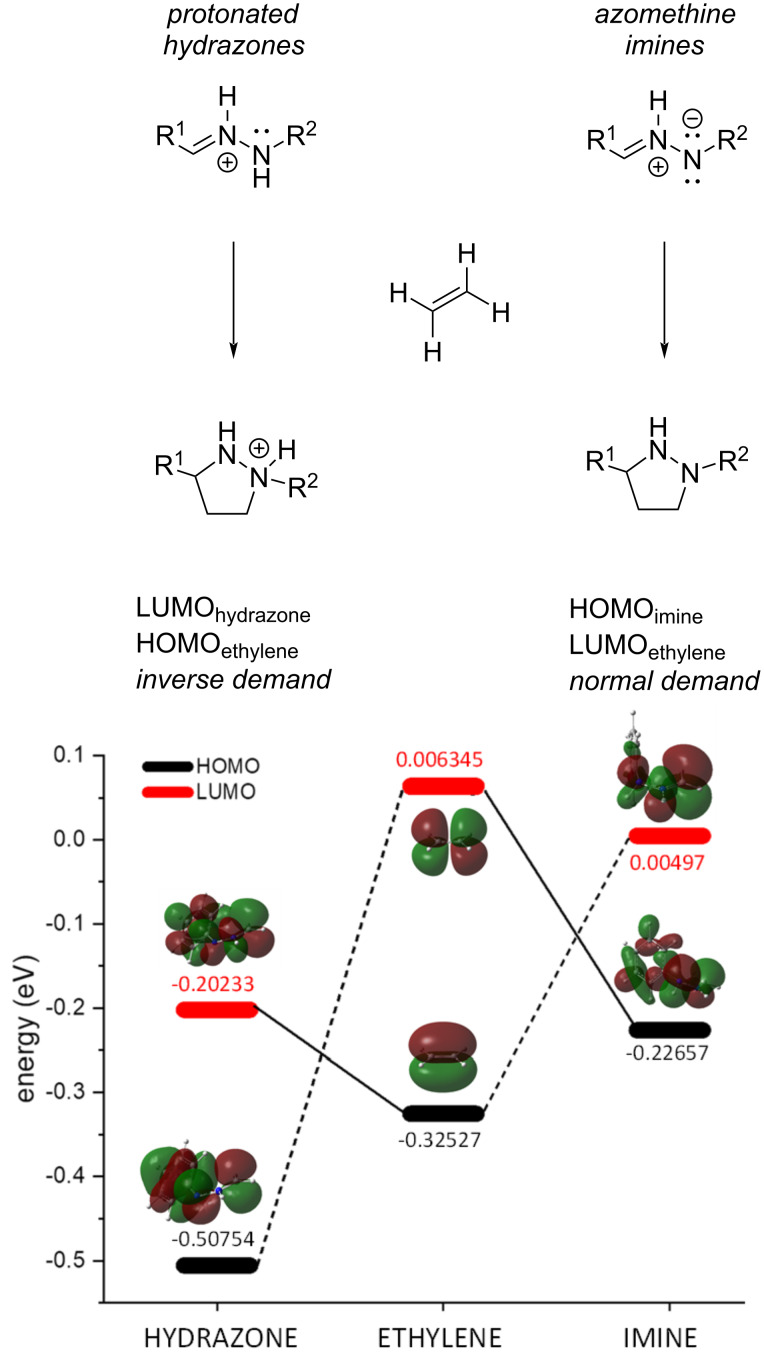
Top: Cycloaddition of protonated hydrazones as inverse-demand reaction of cycloaddition of azomethine imines. Bottom: molecular orbitals involved in the reaction and energy diagram of the reactivity of ethylene with azomethine imine and protonated hydrazone. Energy (eV) of molecular orbitals has been calculated at the m062x/6-311+G(d,p)/SMD=toluene level of theory.

The reaction has been defined by Houk and Rueping as a (3^+^ + 2) monopolar cycloaddition [33] pointing out the protonated state of the imino nitrogen of the hydrazone in contrast to the well-known 1,3-dipolar cycloaddition of azomethine imines in which the terminal nitrogen has a negative charge. While both reacting C–N–N systems fulfil the requirements to give a cycloaddition with an alkene; which are (i) electron density default on the carbon atom and (ii) an electron density excess on the nitrogen atom; the overall positive charge of the hydrazone moiety forces a role inversion of the reagents and whereas in the classical cycloadditions with azomethine imines, they act as a nucleophile (involving their HOMO, interacting with the LUMO of the alkene), in our case, the protonated hydrazone acts as an electrophile (involving their LUMO, interacting now with the HOMO of the alkene) (Figure 2). Thus, we can consider the reaction of hydrazones with alkenes an inverse-demand cycloaddition with respect to that of azomethine imines (Figure 2).

**Figure 3:**
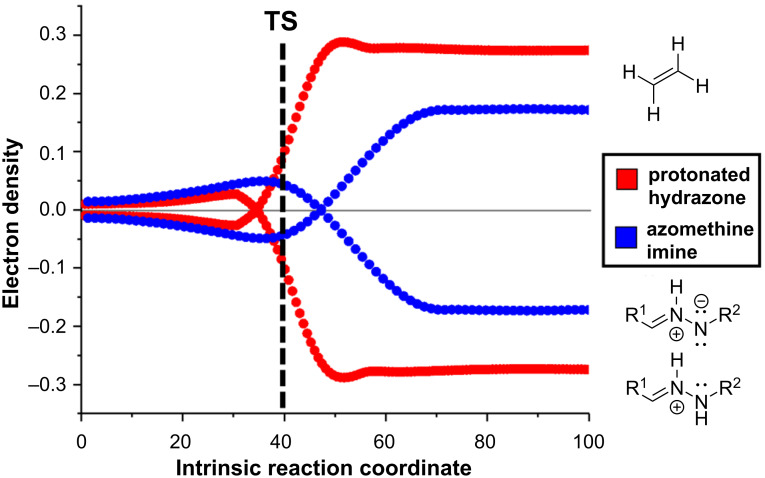
Global electron density transfer (GEDT). Dashed black line indicates both TS.

In fact, we monitored the global electron density transfer (GEDT) [34] between the reagents along the reaction coordinate (Figure 3) and we found, in both cases, values lower than 0.4, typical for nonpolar processes.

**Figure 4:**
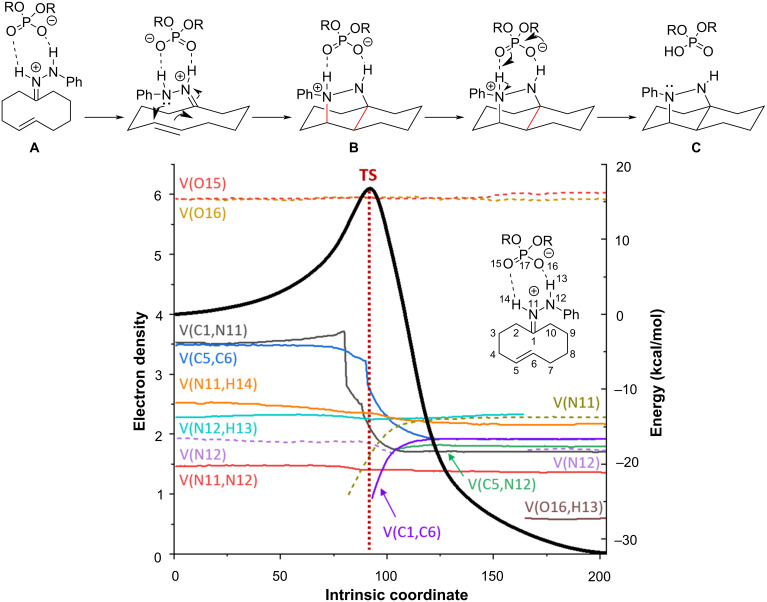
ELF analysis for the reaction of series **b** leading to a system 6-6. Black trace corresponds to IRC. Colored dotted traces refer to lone pairs (monosynaptic basins) and colored plain traces to bonds (disynaptic basins). The vertical red line indicates the transition state (see Supporting Information File 1 for the full data).

To assess the concertedness of the reaction we carried out an analysis of the electron localization function (ELF) [31–32]. The ELF analysis applied to an IRC represents the evolution of the electron density (electron population) during the whole reaction. In consequence, it is possible to analyze the concertedness of the reaction by establishing the moment in which a given bond is broken or formed as well as to analyze changes in the electronic population in bonds and atoms with lone pairs. The ELF analysis of the reaction corresponding to series **b**, leading to a 6-6 system is illustrated in Figure 4.

The first event corresponds to lowering the V(C1,N11) electron population with concomitant decreasing of V(C5,C6) corresponding to the C=N and C=C bonds, respectively. At the same time both V(N11) and V(C1,C6) appear and increase their population till ca. 2 e^−^ indicating the formation of the C1–C6 bond and the pyramidalization of N11. Although the next bond, corresponding to basin V(C5,N12) seems to be formed with some delay, we can consider the cycloaddition as a highly concerted process, because the bond formation occurs during the other reacting basin’s evolution. During the process, N12 loses its lone pair which is involved in the C5–N12 bond formation, and after the cyclization reaction, in a second stage, the lone pair is recovered after the proton (H13) is abstracted by the phosphoric acid. As expected, no significant variations are observed for the bonds N11–N12 and N11–H14, confirming that protonation of N11 is maintained during all the process. In summary, we can define the whole situation as a concerted process taking place in two stages, i.e.: (i) the first one comprises a series of concomitant events in which all bonds involved in the cycloaddition are formed and broken and (ii) a second one consisting of the deprotonation of the nitrogen yielding a neutral compound and liberating the catalyst.

**Figure 5:**
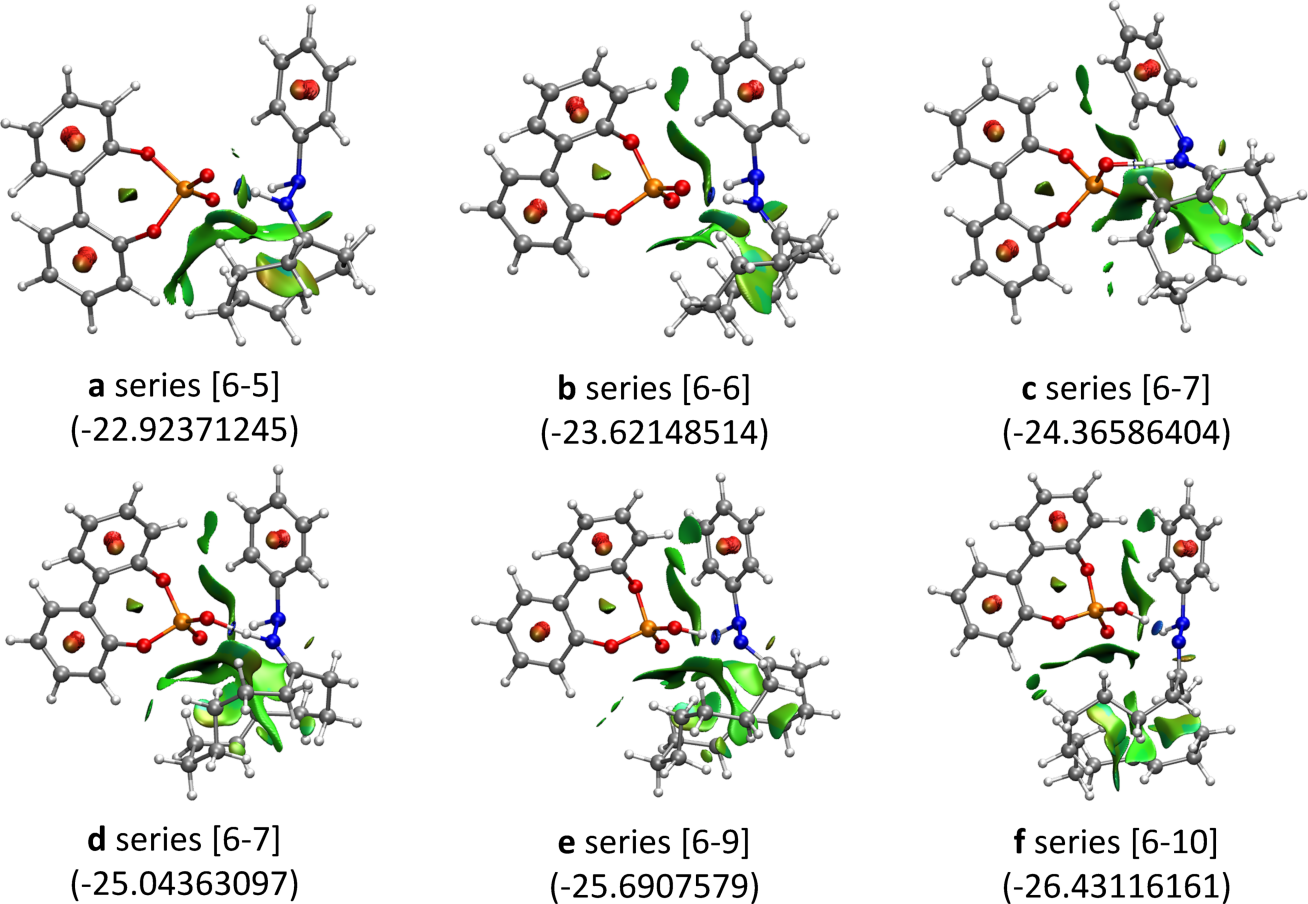
Quantitative NCI analysis [36] for the reaction of series **a**–**f** leading to fused cyclohexanes. The resulting system is given in square brackets. In parentheses the integration over the volumes of −λ^2^·ρ^2^ representing the total integration data corresponding to the weak noncovalent van der Waals interactions (represented in green). Higher forces like H-bonds are indicated as blue discs. In red unfavorable interactions are represented.

The comparative quantitative analysis of the noncovalent interactions (NCI) [35–36] of fused cyclohexanes clearly showed that NCIs increase with the size of the tether (Figure 5) and the same effect is expected to account for the reagents. In general, a barrier could increase either by a destabilization of the transition structure or the stabilization of the reagents. In our case, the more stable is the reagent the higher is the barrier (for similar transition-state energies) justifying the observed reactivity. Only the two systems (**a** and **b**) with lower values of the integration parameter have barriers compatible with observed reactions upon heating, the rest presenting too high barriers to allow the reaction.

**Figure 6:**
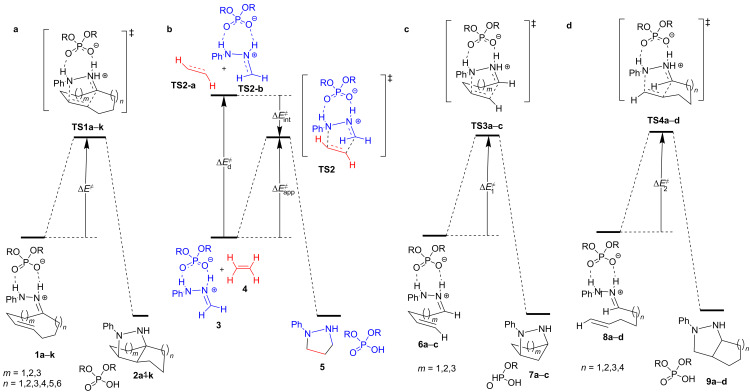
(a) Transannular cycloadditons of compounds **1a**–**k**. (b) Houk’s distortion model applied to the reactions. **TS2-a** and **TS2-b** have been calculated separately. (c) and (d) Model reactions with tether **A** and **B**, respectively, to be subjected to the Houk’s distortion model, too. Compounds **7a**–**c** and **9a**–**d** have not been calculated because they are not relevant for the distortion analysis.

To investigate further the origin of the observed lack of reactivity for medium- and long-size tethers we applied the Houk’s distortion model [30] to our substrates using the modified model [9] that allows the analysis of intramolecular reactions. According to that modified model, the two reactive moieties, hydrazone and double bond, are separated from the tethers and capped with hydrogens resulting in hydrazone **3** and ethylene (**4**). In that way, the reactive components preserve the original geometries adopted during the transannular reactions (Figure 6a and 6b). Following the Houk model [30] the distortion energy (Δ*E*^≠^_d_) corresponds to the difference between the single point corresponding to interacting **3** and **4**, and the sum of single-point calculations for **TS2-a** and **TS2b**.

The apparent activation energy (Δ*E*^≠^_app_) refers to the energy difference between **TS2** and the interacting reagents **3** and **4** (single point calculations). The difference between Δ*E*^≠^_app_ and Δ*E*^≠^ is the distortion energy of the tether (Δ*E*^≠^_tether_) (Equation 1).


[1] $$\begin{array}{c} \Delta E_{\text{app}}^{\neq }=\Delta E_{\text{d}}^{\neq }+\Delta E_{\mathrm{int}}^{\neq }\\ \Delta E_{\text{tether}}^{\neq }=\Delta E^{\neq }-\Delta E_{\text{app}}^{\neq }\\ \Delta E_{\text{tether}}^{\neq }=\Delta E_{\text{t}1}^{\neq }+\Delta E_{\text{t}2}^{\neq }+\Delta E_{\text{ti}}^{\neq } \end{array}$$

**Scheme 2:**
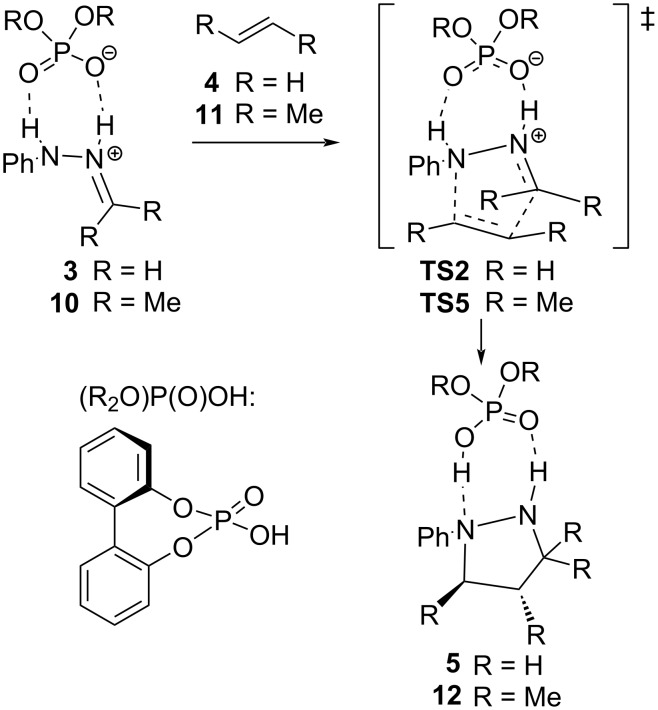
Reaction with simple models.

Since our system has two different tethers, we introduced an additional modification by calculating single points of the system with just one tether (Figure 6c and 6d) in order to estimate the individual contribution of each tether to the energy distortion. The corresponding model reactions for tether **A** (**6a**–**c** to give **7a**–**c** through **TS3a**–**c**) and tether **B** (**8a**–**d** to give **9a**–**d** through **TS4a**–**d**) were constructed by eliminating the other tether and keeping the original geometry. From these calculations and applying the Houk’s model we can obtain the corresponding distortion energy for each different tether (Δ*E*^≠^_t1_ and Δ*E*^≠^_t2_; t1 and t2 correspond to tether **A** and **B**, respectively). Besides, we have added an additional term (Δ*E*^≠^_ti_) corresponding to the penalty (or synergy) exerted by the combination of the two tethers, which might force additional distortion (or relaxation). That term can be directly calculated from the values obtained by applying the Houk’s distortion model to the full system (Δ*E*^≠^_tether_) and each of the isolated tether (Δ*E*^≠^_t1_ and Δ*E*^≠^_t2_). The obtained value for the analysis of the full systems should be the sum of each tether calculated from the individual models plus the term representing the interaction exerted by the combination of both tethers, i.e.: Δ*E*^≠^_tether_ = Δ*E*^≠^_t1_ + Δ*E*^≠^_t2_ + Δ*E*^≠^_ti_ (Equation 1). In addition, and for the purpose of comparison, we also calculated the bimolecular reactions for simple models capped with hydrogen and methyl groups without restrictions (Scheme 2).

The calculated activation parameters for bimolecular cycloadditions for R = H and R = Me are given in Table 2.

**Table 2:** Calculated activation parameters for transannular cyclizations illustrated in Scheme 2.^a^

reactant	product	Δ*E*(0)^≠^	Δ*H*^≠^	−*T*·Δ*S*^≠^	Δ*G*^≠^

**3**	**4**	22.2	20.7	4.2	25.9
**10**	**12**	6.8	5.3	4.6	11.1

^a^Level: m062x/6-311+G(d,p)/SMD=toluene//m062x/6-31G(d).

Table 3 and Table 4 collect the analyses for fused cyclohexanes and cyclopentanes, respectively.

**Table 3:** Distortion–interaction analysis for fused cyclohexanes.

	**1a**	**1b**	**1c**	**1d**	**1e**	**1f**

system	6-5	6-6	6-7	6-8	6-9	6-10
*ΔE* ^≠^_d,dipole_	28.6	27.0	25.7	27.2	26.5	33.9
Δ*E*^≠^_d,alkene_	4.0	7.1	6.4	6.2	7.9	7.7
Δ*E*^≠^_d,total_	32.6	34.1	32.0	33.4	34.4	41.6
Δ*E*^≠^_int_	−22.7	−23.4	−20.7	−22.8	−23.3	−23.2
Δ*E*^≠^_app_	9.9	10.8	11.3	10.7	11.2	18.4
Δ*E*^≠^	20.9	19.8	26.2	31.2	27.4	31.3
Δ*E*^≠^_d,tether_	11.0	9.0	14.9	20.5	16.2	12.9
Δ*E*^≠^_t1_	3.5	7.2	7.2	6.3	8.7	3.0
Δ*E*^≠^_t2_	11.2	6.9	12.4	18.4	14.6	10.7
Δ*E*^≠^_til_	−3.8	−5.0	−4.7	−4.2	−7.1	−0.8

^a^Level: m062x/6-311+G(d,p)/SMD=toluene//m062x/6-31G(d).

**Table 4:** Distortion-interaction analysis for fused cyclopentanes and system 7-8.

	**1g**	**1h**	**1i**	**1j**	**1k**

system	5-5	5-6	5-7	5-10	7-8
Δ*E*^≠^_d,dipole_	19.4	18.6	27.0	30.5	38.5
Δ*E*^≠^_d,alkene_	8.2	9.8	11.0	12.3	7.5
Δ*E*^≠^_d,total_	27.6	28.3	38.1	42.7	46.0
Δ*E*^≠^_int_	−11.1	−11.9	−13.1	−16.6	−23.2
Δ*E*^≠^_app_	16.4	16.4	25.0	26.1	22.8
Δ*E*^≠^	23.2	20.8	27.9	31.8	31.8
Δ*E*^≠^_d,tether_	6.8	4.4	2.9	5.7	9.1
Δ*E*^≠^_t1_	1.8	6.7	1.5	2.8	8.1
Δ*E*^≠^_t2_	2.6	1.4	0.9	2.3	16.6
Δ*E*^≠^_ti_	2.4	−3.7	0.5	0.5	−5.8

^a^Level: m062x/6-311+G(d,p)/SMD=toluene//m062x/6-31G(d).

All the reactions leading to fused cyclohexanes (Table 3) increased tether strain in the transition structures, showing a negative contribution of the tether to the reactivity. The reaction of **1b** has the lowest tether strain, because a decalin is formed. Relatively low values are also observed for the reaction of **1a** and **1f**. However, in the case of **1f** a high distortion of the reactants (particularly of the dipole) is observed contributing to a high barrier. Consequently, only the reactions of **1a** and **1b** are expected to be experimentally observed, as it is confirmed by our previous results. The rest of the compounds are also predicted to be difficult to undergo the transannular reaction as a consequence of the high distortion of the tether. In the case of compounds **1e** distortion of the reagents also contributes to the lack of reactivity.

Interestingly, the Houk’s distortion analysis applied to individual tethers shows that tether **B** (Δ*E*^≠^_t2_) contributes to a greater extent to the barrier of the reaction. Only in the case of the experimentally observed 6-6 both tethers present a similar contribution. Indeed, this case is the only one in which tether **B** (Δ*E*^≠^_t2_) has a value lower than 10 kcal/mol being the main responsible of the lack of reactivity. Notably, in all cases, Δ*E*^≠^_ti_ has a negative value indicating that both tethers have a synergic effect, although in any case it is not enough for favoring the reaction. In the case of fused cyclopentanes we observed similar results predicting the only reaction of the observed 5-6 system.

The differences observed between the terms of the distortion analysis corresponding to the transition structures (Figure 6, **TS1a**–**k**) revealed very similar energies, in the range of 2 kcal/mol for both fused cyclohexanes and cyclopentanes, with the exception of the highly constrained system 5-5. Considering the simplest bimolecular model **3**, the fused cyclohexanes contribute with ca. 6–8 kcal/mol while the fused cyclopentanes contribute with ca. 15 kcal/mol (Table 5).

**Table 5:** Comparison of relative energies (kcal/mol) between transition structure terms of distortion analysis.

system	relative energy^a^

6-5	8.0
6-6	7.2
6-7	6.1
6-8	7.7
6-9	6.8
6-10	7.2
5-5	20.2
5-6	15.4
5-7	14.7
5-10	14.8

^a^Relative to transition structure **TS2**.

## Conclusion

In conclusion, the computational topological study (ELF and NCI analysis) of a series of transannular cycloadditions of hydrazones catalyzed by BINOL phosphoric acids, indicated that the process is an apolar concerted process in which all the events (bonds breaking/formation) take place in a concomitant way. In spite of the polarity of the reacting groups the global charge transfer is not so high to be considered a polar process. The reaction, as previously reported by the classical intermolecular reaction takes place smoothly by the action of the organocatalyst that renders a protonated hydrazone as the reacting functional group. However, in several cases the reaction does not work. Application of the Houk's distortion model to those reactions suggested that the observed lack of reactivity for reactions involving the formation of medium-size fused rings is mainly due to the negative effect of the tethers consisting of allowing a more stable (i.e., less distorted) disposition of the involved functional groups, leading to more stable reagents (rather than more unstable transition structures) which results in an increase of the reaction barrier. In fact NCI analyses points in the same direction. The combined effect of the two tethers is less negative than a simple additive effect as results from the comparison between global and individual distortion analyses of the tethers. Application of these methodologies can be used to predict the reactivity of different substrates in other transannular cycloadditions.

## Supporting Information

File 1 Computational methods, energies, and Cartesian coordinates.
