# Supplementary material for: Computational studies of Brønsted acid-catalyzed transannular cycloadditions of cycloalkenone hydrazones
Source: Beilstein J Org Chem. 2023 Apr 20;19:477–86. doi: 10.3762/bjoc.19.37 (PMC10130903; doi:10.3762/bjoc.19.37)
Supplement: File 1 — Computational methods, energies, and Cartesian coordinates. [file Beilstein_J_Org_Chem-19-477-s001.pdf]

BEILSTEIN JOURNAL OF ORGANIC CHEMISTRY

Supporting Information

for

Computational studies of Brønsted acid-catalyzed transannular cycloadditions of cycloalkenone hydrazones

Manuel Pedrón, Jana Sendra, Irene Ginés, Tomás Tejero, Jose L. Vicario and Pedro Merino

Beilstein J. Org. Chem. **2023,** *19,* 477–486. doi:10.3762/bjoc.19.37

Computational methods, energies, and Cartesian coordinates

License and Terms: This is a supporting information file under the terms of the Creative Commons Attribution License (https://creativecommons.org/licenses/by/4.0). Please note that the reuse, redistribution and reproduction in particular requires that the author(s) and source are credited and that individual graphics may be subject to special legal provisions.
The license is subject to the *Beilstein Journal of Organic Chemistry* terms and conditions: (https://www.beilstein-journals.org/bjoc/terms)

Computational methods

All of the calculations were performed using the Gaussian16 program [1]. Computations were done using the m062x functional [2] in conjunction with standard basis sets 6-31G(d) and 6-311+G(d,p)[3,4]. Geometry full optimizations were made at the m062x/6-31G(d) level. Single point calculations using the 6-311+G(d,p) basis set were carried out over optimized geometries to obtain the energy values. Solvent effects (toluene) were considered using the SMD model [5]. The nature of stationary points was defined on the basis of calculations of normal vibrational frequencies (force constant Hessian matrix). Minimum energy pathways for the reactions studied were found by gradient descent of transition states in the forward and backward direction of the transition vector (IRC analysis) [6]. Analytical second derivatives of the energy were calculated to classify the nature of every stationary point, to determine the harmonic vibrational frequencies, and to provide zero-point vibrational energy corrections. The thermal and entropic contributions to the free energies were also obtained from the vibrational frequency calculations, using the unscaled frequencies. Correction to free energy was made by substracting S_{trans} contribution and considering a 1 M concentration [7]. The conformational searches were performed with the software Macromodel as implemented in the Schrödinger package [8]. A mixed torsional/low-mode sampling method with OPLS4 force field and chloroform as a solvent was used. The cutoff for maximum atom deviation was 0.5 and an energy window of 20 kcal/mol was selected. The resulting conformers in a range of 5.0 kcal/mol were optimized at the level m062x/6-31G(d,p) to locate the global minimum in the case of starting compounds. The conformational variability of the transition structures was carried out in a similar way by using constrained searches. Structural representations were generated using CYLView [9].

ELF analysis

The electronic structures of stationary points were analyzed by the topological analysis of the gradient field of electron localization function (ELF) [10] developed by Silvi and Savin [11,12]. The ELF study was performed with the TopMod program [13] using the corresponding wavefunctions of the all structures of the IRC. The topological analysis of the

gradient field of ELF has showed to be a powerful tool for the study of the bonding changes along an organic reaction [14-16].

NCI Calculations

NCI (non-covalent interactions) were computed using the methodology previously described [17]. Data were obtained with the NCIPLOT4 program [18]. A density cutoff of ρ = 0.1 a.u. was applied and the pictures were created for an isosurface value of s = 0.35 and colored in the [−0.03, 0.03] a.u. sign(λ_2)ρ range using VMD software[19].

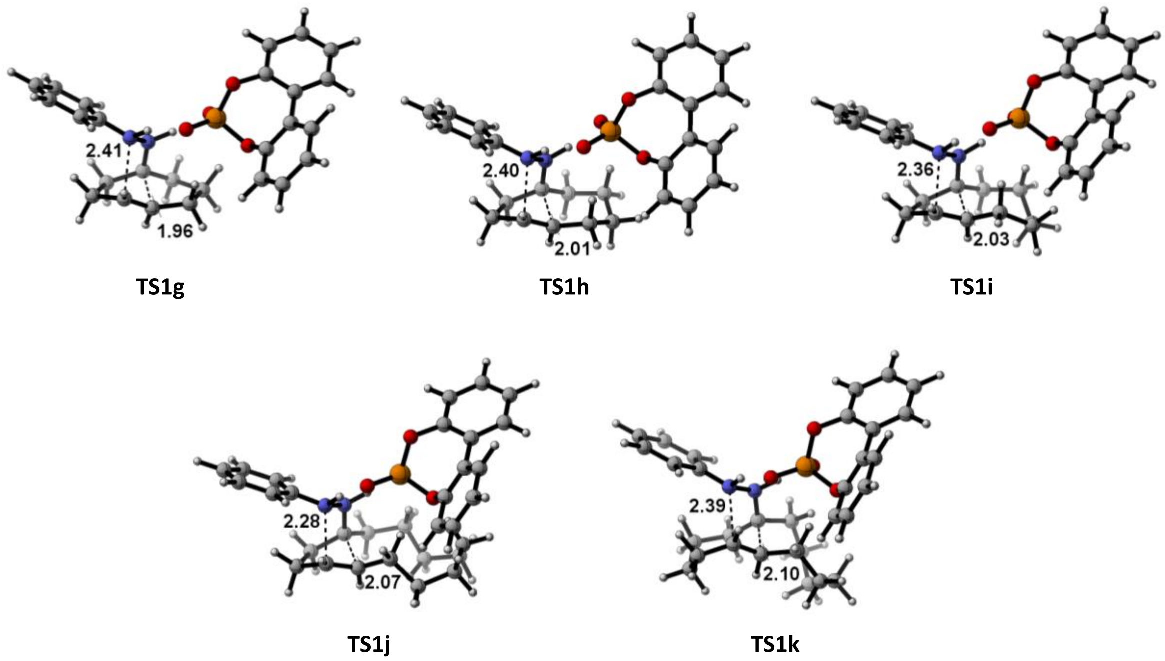

Figure S1: Optimized (m062x/6-31G(d)) geometries for transition structures of series **g**–**k**.

Energies

Table S1: Calculated (m062x/6-311+G(d,p)/SMD=toluene//m062x/6-31G(d)) absolute (hartree) and relative (kcal/mol) energies for transannular cycloadditions of compounds **1**.

	E_0	ΔE_0	G	ΔG	im. freq
**1a**	-1797.447918	0.0	-1797.529036	0.0	
**TS1a**	-1797.413925	21.3	-1797.492133	23.2	-380.8
**2a**	-1797.471650	-14.9	-1797.548721	-12.4	
**1b**	-1836.729861	0.0	-1836.813253	0.0	
**TS1b**	-1836.697226	20.5	-1836.777615	22.4	-404.0
**2b**	-1836.766268	-22.8	-1836.843608	-19.0	
**1c**	-1876.006876	0.0	-1876.090586	0.0	
**TS1c**	-1875.963502	27.2	-1876.044729	28.8	-385.0
**2c**	-1876.028483	-13.6	-1876.109301	-11.7	
**1d**	-1915.286933	0.0	-1915.372402	0.0	
**TS1d**	-1915.235568	32.2	-1915.318738	33.7	-405.4
**2d**	-1915.295914	-5.6	-1915.377698	-3.3	
**1e**	-1954.557859	0.0	-1954.646316	0.0	
**TS1e**	-1954.511988	28.8	-1954.595926	31.6	-433.1
**2e**	-1954.569735	-7.5	-1954.653154	-4.3	
**1f**	-1993.803082	0.0	-1993.893454	0.0	
**TS1f**	-1993.746446	35.5	-1993.833212	37.8	-430.6
**2f**	-1993.841907	-24.4	-1993.927252	-21.2	
**1g**	-1758.190032	0.0	-1758.268049	0.0	
**TS1g**	-1758.153318	23.0	-1758.229741	24.0	-416.8
**2g**	-1758.116045	46.4	-1758.190950	48.4	
**1h**	-1797.445530	0.0	-1797.526123	0.0	
**TS1h**	-1797.411541	21.3	-1797.488085	23.9	-420.2
**2h**	-1797.479896	-21.6	-1797.557943	-20.0	
**1i**	-1836.728570	0.0	-1836.810278	0.0	
**TS1i**	-1836.683966	28.0	-1836.763629	29.3	-458.8
**2i**	-1836.748536	-12.5	-1836.827086	-10.5	
**1j**	-1954.560027	0.0	-1954.646694	0.0	
**TS1j**	-1954.507496	33.0	-1954.590663	35.2	-486.1
**2j**	-1954.565691	-3.6	-1954.647864	-0.7	
**1k**	-1954.561858	0.0	-1954.651974	0.0	
**TS1k**	-1954.508163	33.7	-1954.592010	37.6	-411.9
**2k**	-1954.575287	-8.4	-1954.658337	-4.0	

Table S2: Calculated (m062x/6-311+G(d,p)/SMD=toluene//m062x/6-31G(d)) absolute (hartree) and relative (kcal/mol) energies for bimolecular reactions.

	E_0	ΔE_0	G	ΔG	im. freq
**8a**	-1564.12925672	0.0	-1564.20746098	0.0	
**TS4a**	-1564.09905418	19.0	-1564.17129663	22.7	-407.6
**9a**	-1564.17401478	-28.1	-1564.24538807	-23.8	
**8b**	-1721.23428241	0.0	-1721.32057330	0.0	
**TS4b1**	-1721.22339271	6.8	-1721.30281127	11.1	-452.5
**TS4b2**	-1721.21820334	10.1	-1721.29790940	14.2	-432.8
**9b1**	-1721.28739838	-33.3	-1721.36520349	-28.0	
**9b2**	-1721.26418383	-18.8	-1721.34256928	-13.8	

Cartesian coordinates

1a

0 1

C	3.5348847800	-0.1536675381	0.6585497237
N	2.6584844737	0.6691170458	0.2015516999
N	2.7165154750	1.1307998226	-1.1307712291
H	1.9433698475	0.6227578259	-1.5918012418
O	0.2560512509	-0.1702624973	-1.4360447987
C	3.1396812261	-2.7990405834	-0.2843875177
C	2.0112230676	-2.4978829648	0.3631897633
H	1.2544762282	-1.9255364987	-0.1757836170
H	3.9300841056	-3.3593150974	0.2220542606
O	0.1814037177	1.0502811849	0.8506782134
P	-0.5008880070	0.2140012353	-0.2039703665
O	-1.0336140192	-1.1204853833	0.5958784053
O	-1.9122470776	0.8788334155	-0.6652032524
C	-2.0167374755	-1.9102117750	0.0379901315
C	-2.9444751280	0.9958080569	0.2428598447
C	-3.3480120562	-1.4712242035	0.0512712335
C	-1.6726524878	-3.1680907678	-0.4429131761
C	-3.7015920737	-0.1342103567	0.5782967670
C	-3.2591452745	2.2559349201	0.7355756097
C	-4.3286149239	-2.3460548595	-0.4271736455
C	-2.6658159902	-4.0191878278	-0.9149290778
H	-0.6272882810	-3.4604946613	-0.4356230995
C	-4.8063722647	0.0471660572	1.4168382218
C	-4.3608432088	2.4101458635	1.5694555260
H	-2.6326288468	3.0930600338	0.4477683293
C	-3.9972279248	-3.6091260854	-0.9043421397
H	-2.3985884498	-5.0010567314	-1.2926236959
C	-5.1392302321	1.3046463028	1.9071348123
H	-4.6098382784	3.3932264743	1.9563350753
H	-4.7750473085	-4.2677196005	-1.2769674867
H	-5.3625050404	-2.0122856208	-0.4383169862
H	-5.3941857681	-0.8220307058	1.6987719757
H	-5.9976017379	1.4198851416	2.5610725048
C	2.4527792465	2.5333008461	-1.2002103412
C	1.4660214478	3.0011428917	-2.0675642512
C	3.2116931306	3.4280347083	-0.4470892458
C	1.2583526260	4.3708038481	-2.1927282263
H	0.8552923608	2.2877650216	-2.6147589820
C	2.9822614601	4.7943831799	-0.5650868351
H	3.9793445332	3.0504389681	0.2232243759
C	2.0115170833	5.2712469709	-1.4429666679
H	0.4890310754	4.7331885195	-2.8672730001
H	3.5710538149	5.4895011053	0.0254482362
H	1.8388998755	6.3384029648	-1.5369470893
C	3.4495911563	-2.1992973560	-1.6233719048
H	2.5488414600	-1.6867546952	-1.9812382078
H	3.7145208230	-2.9543584519	-2.3718375279

C	3.3766008952	-0.6280363473	2.0886910645
H	3.5654749420	0.2445203894	2.7276182560
H	4.1722975025	-1.3511471146	2.2922698904
C	1.8063824226	-2.6366349861	1.8363720141
H	0.7932745704	-2.9774910620	2.0744971291
H	2.5229162569	-3.3499202252	2.2627625823
C	2.0136859791	-1.2435660484	2.4681114827
H	1.9763671747	-1.3251748827	3.5591682279
H	1.1956841634	-0.5777466805	2.1723491934
C	4.7720977142	-0.4867136241	-0.1379378361
H	5.2965564456	0.4629879957	-0.3034555608
H	5.4034969926	-1.0982552953	0.5106501545
C	4.6163435979	-1.1875297979	-1.5144932069
H	4.5028435994	-0.4288395142	-2.2901092679
H	5.5672930396	-1.6995688878	-1.6959676016
H	1.7235622927	0.8971379382	0.6896523785

1b

0 1

C	3.5232971746	-0.7294440241	0.7684440795
N	2.5837704364	0.0757270555	0.4225202090
N	2.6047578734	0.7387089536	-0.8247641326
H	1.8815186824	0.2464381171	-1.3761563294
O	0.1952425418	-0.5681215139	-1.3804245199
C	2.6086983202	-2.9154829200	-0.7566722719
C	2.3752469917	-3.5298108131	0.4035038782
H	3.1511779856	-4.1807316773	0.8188314656
H	1.8346764237	-2.2675086884	-1.1784678659
O	0.0957396458	0.2733895177	1.0681056670
P	-0.6127945841	-0.0950512677	-0.2135262298
O	-1.7603316817	-1.1734116867	0.2228860583
O	-1.5406657107	1.1388303671	-0.7419927333
C	-2.8605977849	-1.3872072836	-0.5796839033
C	-2.5767941635	1.5860096269	0.0499226106
C	-3.8896271065	-0.4356647050	-0.6230610466
C	-2.9602534388	-2.5952478955	-1.2590224789
C	-3.7686756690	0.8518231467	0.0970738156
C	-2.4350321886	2.7958222935	0.7177597382
C	-5.0355080632	-0.7487804473	-1.3614316437
C	-4.1085923855	-2.8819694914	-1.9884341856
H	-2.1311962583	-3.2918334993	-1.1945080452
C	-4.8345658020	1.3871380732	0.8273494933
C	-3.5087161617	3.3055406643	1.4394199230
H	-1.4826916071	3.3124028029	0.6509258194
C	-5.1511182291	-1.9588865927	-2.0360557290
H	-4.1877893898	-3.8249344047	-2.5201312770
C	-4.7115479167	2.6029001780	1.4906096775
H	-3.4054029904	4.2501032837	1.9642557276
H	-6.0477993170	-2.1758300589	-2.6075270381
H	-5.8342273166	-0.0142918083	-1.4173678956
H	-5.7617246481	0.8237129155	0.8865669437
H	-5.5496125016	2.9960465068	2.0570929363
C	2.2070981452	2.1042281386	-0.6718475231
C	2.8445541106	2.9208689837	0.2617203176

C	1.2064198486	2.6216036857	-1.4941340994
C	2.4745182861	4.2563163820	0.3749482979
H	3.6255125607	2.5074328312	0.8942410905
C	0.8548145499	3.9630242688	-1.3832103826
H	0.6862795781	1.9645452636	-2.1856021589
C	1.4827019208	4.7841279596	-0.4491801112
H	2.9683816916	4.8885103031	1.1063738169
H	0.0690020722	4.3594689776	-2.0186414199
H	1.1987843209	5.8277650810	-0.3606664451
C	3.9356440424	-2.9486918174	-1.4550159261
H	3.8323621287	-3.2953068161	-2.4903783703
H	4.6089974390	-3.6483469878	-0.9430331627
C	3.4991786098	-1.3298112943	2.1507598235
H	4.0649331966	-2.2658699052	2.1159664993
H	4.1194031119	-0.6340671876	2.7379446067
C	1.1554517159	-3.3407683744	1.2561291064
H	0.4934570699	-2.5889994199	0.8105536264
H	0.5854297697	-4.2778896906	1.3062819157
C	1.5310477753	-2.9375826571	2.6902983645
H	0.6303790461	-2.9751959810	3.3118360213
H	2.2229020880	-3.6882476635	3.0994875337
C	2.1481739032	-1.5432574339	2.8620254362
H	2.3263126679	-1.3928476902	3.9318790251
H	1.4116040527	-0.7884114413	2.5723349856
C	4.7518705412	-0.9070596990	-0.0899553817
H	5.1714466097	0.0956037710	-0.2389381118
H	5.4756997698	-1.4937154976	0.4847524759
C	4.5550316291	-1.5440658886	-1.4827667203
H	3.9339787305	-0.8770020749	-2.0844534190
H	5.5402585900	-1.5816511901	-1.9603762095
H	1.6278832672	0.1735293396	0.9191017816

1c

0 1

C	3.6116452867	0.7777531272	1.3124553121
N	2.5066991823	1.0901669879	0.7392298292
N	2.4743634788	1.4940479923	-0.6155597817
H	1.9847145490	0.7124982721	-1.0785407145
H	1.4836900905	0.9641072742	1.1497746715
O	0.5437729714	-0.5529652154	-0.8845819457
C	3.6604984833	-1.8229729301	-0.2752786325
C	3.8456449567	-2.6661661942	0.7432656960
H	4.8497793572	-2.7897547915	1.1595051212
H	2.6554500817	-1.7024361166	-0.6850581829
O	0.0249483584	0.8221468598	1.2402266782
P	-0.4647556595	-0.0217259869	0.0831458210
O	-1.3772608712	-1.1966616045	0.7575341316
O	-1.5926890425	0.7842216588	-0.7750952437
C	-2.3541501426	-1.8407797009	0.0280584554
C	-2.7504723043	1.1807725513	-0.1383336551
C	-3.5566720738	-1.1833777189	-0.2704401897
C	-2.1538694149	-3.1733231725	-0.3115650644
C	-3.7605249166	0.2380270736	0.0880924634
C	-2.9077367198	2.5197233858	0.1960188891

C	-4.5597037033	-1.9186642985	-0.9104107459
C	-3.1655474252	-3.8820529749	-0.9496383628
H	-1.2038269636	-3.6318495270	-0.0589720934
C	-4.9614873286	0.6913070064	0.6433813758
C	-4.1103299961	2.9448425985	0.7497803042
H	-2.0804388514	3.1980847234	0.0111938131
C	-4.3737847017	-3.2548211836	-1.2458492398
H	-3.0090298363	-4.9226694296	-1.2156155461
C	-5.1402925628	2.0312057606	0.9684853645
H	-4.2422208925	3.9893454485	1.0141966361
H	-5.1654404458	-3.8017069037	-1.7476912873
H	-5.4900097315	-1.4172749260	-1.1626550030
H	-5.7511889239	-0.0288321692	0.8391292830
H	-6.0778408656	2.3602219562	1.4050459924
C	1.7014693886	2.6849571762	-0.7833683360
C	1.9640350126	3.8094589078	-0.0019694415
C	0.7166357855	2.7262994175	-1.7699000544
C	1.2356945348	4.9758382747	-0.2081173126
H	2.7332707901	3.7664912299	0.7641854182
C	0.0051814494	3.9030757062	-1.9787693392
H	0.4887770401	1.8295207003	-2.3394687951
C	0.2585557341	5.0304056719	-1.1998728389
H	1.4376425002	5.8480622893	0.4058620175
H	-0.7674513021	3.9277751939	-2.7409488261
H	-0.3050648265	5.9437164056	-1.3606934302
C	4.7822473849	-1.0235462359	-0.9095053359
H	4.3718018414	-0.3218062705	-1.6434426260
H	5.4401894801	-1.7015179657	-1.4670241668
C	3.5918140444	0.2356720178	2.7142688320
H	4.3170525276	-0.5894138228	2.7293211598
H	4.0256483972	1.0202048403	3.3504658681
C	2.2373891171	-0.2202682834	3.2668884634
H	2.3803229586	-0.4551540573	4.3282028868
H	1.5195213381	0.6057234224	3.2263610301
C	4.9361665069	1.0187743262	0.6478249621
H	4.7876632247	1.7297853281	-0.1671166894
H	5.5868008568	1.4752896762	1.4037249922
C	5.6310916561	-0.2442348578	0.1023392812
H	6.5612879228	0.0899805250	-0.3688951198
H	5.9215953760	-0.8981282229	0.9317295720
C	1.6471007653	-1.4377912611	2.5470616711
H	1.6216458802	-1.2422575838	1.4702957194
H	0.5991014581	-1.5360316152	2.8444465360
C	2.7655135851	-3.4368477608	1.4463471077
H	1.8701175295	-3.4857184592	0.8152292155
H	3.1001525011	-4.4653135664	1.6256751639
C	2.3998616688	-2.7606377756	2.7813454037
H	3.3215757704	-2.5928125772	3.3575231705
H	1.7903586799	-3.4428886264	3.3830596915

1d

0 1

C	3.5616342085	0.6053990307	-0.9734446540
N	2.6265569070	-0.2355917171	-0.7176984134

N	2.7723583272	-1.2208217305	0.2873786754
H	2.0931034882	-0.9250939760	1.0050788025
O	0.3816711362	-0.0963587602	1.2983791989
C	3.0361085696	1.8781629338	1.8770091066
C	2.5520053429	3.1183414844	1.9687031969
H	3.2393960509	3.9500543993	2.1532604570
H	2.3218625879	1.0703454616	1.7168930243
O	0.1243670067	-0.3689500412	-1.2579175983
P	-0.5100579459	-0.2819570523	0.1116689162
O	-1.6474376095	0.8853554398	-0.0040059887
O	-1.4382818422	-1.5904595724	0.4028623132
C	-2.7170561155	0.9248336243	0.8652202206
C	-2.5157080097	-1.8411613199	-0.4214798952
C	-3.7595590989	-0.0048752785	0.7397077999
C	-2.7787964435	1.9615817458	1.7889426417
C	-3.6925658565	-1.1019646649	-0.2517246516
C	-2.4248971010	-2.8724227261	-1.3476599108
C	-4.8727583278	0.1481945840	1.5727148679
C	-3.8956710827	2.0913528322	2.6065224206
H	-1.9471323796	2.6565743668	1.8420744646
C	-4.7976970680	-1.4489483139	-1.0358175771
C	-3.5368343869	-3.1966659573	-2.1167977946
H	-1.4807530159	-3.3995360618	-1.4416817829
C	-4.9472585713	1.1842944435	2.4965131237
H	-3.9432507425	2.8999825736	3.3289946845
C	-4.7262198773	-2.4873426682	-1.9575212445
H	-3.4737689642	-4.0008761408	-2.8431061794
C	2.3762619712	-2.5058570808	-0.2059327120
C	2.9956728111	-3.0390397614	-1.3352624605
C	1.3989010834	-3.2324310823	0.4730044356
C	2.6319185317	-4.3023275056	-1.7877551226
H	3.7587497021	-2.4634384795	-1.8523857262
C	1.0543547753	-4.5028665275	0.0219297721
H	0.8933027266	-2.7876895246	1.3260135632
C	1.6645465155	-5.0407174610	-1.1090900778
H	3.1114903402	-4.7141016436	-2.6703128827
H	0.2887082460	-5.0636885567	0.5490554574
H	1.3859215787	-6.0281777503	-1.4625009473
C	4.5000366343	1.5387124766	1.9942801303
H	4.6240972661	0.5362397233	2.4210940816
H	4.9794714148	2.2416167476	2.6856134057
C	4.9212843109	0.5014380022	-0.3495435132
H	5.0246835307	-0.4766861206	0.1240714899
H	5.6352136377	0.5695728741	-1.1807122297
C	5.2624365885	1.6135052791	0.6622439906
H	6.3371889276	1.5343645316	0.8588033079
H	5.0959371936	2.5997708584	0.2115966987
C	1.1017438774	3.4678180335	1.7891599160
H	0.5334298964	2.5409715282	1.6448932361
H	0.7162948260	3.9391103174	2.7028997858
C	0.8814439557	4.4360718074	0.6102100400
H	1.2636012569	5.4285051490	0.8809876397
H	-0.1970325272	4.5497103393	0.4422378418
C	1.5576419652	3.9776600358	-0.6854663489
H	2.6448946712	3.9812200048	-0.5265799493
H	1.3579496577	4.7042604431	-1.4836200847

C	1.1013145270	2.5883014357	-1.1249883909
H	1.1989588805	1.8989633008	-0.2799942825
H	0.0321555881	2.6054747657	-1.3655874679
C	3.3277122889	1.6736715318	-2.0066078087
H	3.8830519701	2.5595089791	-1.6779087549
H	3.8458446360	1.3268117294	-2.9127796289
C	1.8703438412	2.0276572362	-2.3245872306
H	1.3445029039	1.1491783092	-2.7112788630
H	1.8852549963	2.7660177936	-3.1352214285
H	-5.6796495326	-0.5755926727	1.4985537426
H	-5.7144892066	-0.8758805293	-0.9282774794
H	-5.8191744121	1.2773644145	3.1357932550
H	-5.5940154631	-2.7346887615	-2.5604490012
H	1.6026844311	-0.2043611284	-1.1214676229

1e

0 1

C	3.4196146226	0.9041673483	-1.0674764330
N	2.3904090693	0.1638742402	-0.8875497146
N	2.4918401691	-0.8583062426	0.1035245539
H	1.8914492368	-0.5587861350	0.8780131733
O	0.0309740655	0.0778048234	1.3096029591
C	3.3871714119	2.0546994547	2.0157590183
C	2.6992185744	3.0327690338	2.6062493696
H	3.2321452685	3.9274625660	2.9397614331
H	2.8414970104	1.1637700781	1.7005318977
O	-0.1994410728	0.1828620641	-1.2816304700
P	-0.8310211176	-0.0961363634	0.1209371175
O	-2.1529066490	0.8245532813	0.0404299328
O	-1.4502926836	-1.5816452121	0.1012819762
C	-3.2963690179	0.4946314126	0.7535924115
C	-2.3649177929	-1.9176545739	-0.8854796654
C	-4.0897174604	-0.5856807775	0.3462410878
C	-3.6675831280	1.3234034992	1.8034265676
C	-3.6835158068	-1.4670541121	-0.7715130507
C	-1.9467922921	-2.7454696123	-1.9172352414
C	-5.2887088729	-0.8002500596	1.0345332329
C	-4.8637406980	1.0867302694	2.4714134184
H	-3.0127315487	2.1458263968	2.0707111732
C	-4.5986178741	-1.8995466691	-1.7372935599
C	-2.8768149212	-3.1590909212	-2.8646403040
C	-5.6777999610	0.0249688000	2.0831036260
H	-5.1577676429	1.7311795139	3.2934128417
C	-4.2031228132	-2.7392540392	-2.7722284431
H	-5.6253366697	-1.5494753251	-1.6789724336
C	2.0317770865	-2.1087721064	-0.3766207060
C	2.2965733027	-2.5237500810	-1.6842963448
C	1.3502672204	-2.9645987226	0.4958820588
C	1.8967715593	-3.7898738968	-2.1038969948
H	2.8171668601	-1.8564346357	-2.3632620272
C	0.9624670157	-4.2276134748	0.0682817436
H	1.1074184226	-2.6204395030	1.4976157711
C	1.2363289284	-4.6518483071	-1.2318681336
H	0.4282154849	-4.8790228758	0.7529171900

C	4.8735527833	2.0430613747	1.8024599984
H	5.3022001276	1.1861931013	2.3411900808
H	5.3130629799	2.9448578311	2.2446376013
C	4.7584163975	0.7055461759	-0.3999396146
H	4.7097803370	-0.1553312509	0.2697019085
H	5.4441377598	0.4519856242	-1.2199374678
C	5.3167519791	1.9406420429	0.3348514959
H	6.4100816402	1.8821759371	0.3138268111
H	5.0484056104	2.8582946063	-0.2009918468
C	1.2157273163	2.9982318828	2.8435647611
H	0.8133647838	2.0439434883	2.4858796237
H	1.0192533296	3.0395787262	3.9231554003
C	0.4816817524	4.1707128889	2.1692185189
H	0.7085050744	5.1013914287	2.7052413799
H	-0.5995450934	4.0099205847	2.2671693391
C	3.3202659315	1.9741282006	-2.1333027281
H	2.7638295161	1.5565751670	-2.9799045543
H	4.3377804600	2.1817072188	-2.4834257115
C	2.6598018016	3.3052471127	-1.7192128819
H	3.1025551765	3.6574946042	-0.7799823020
H	2.9415838921	4.0367551590	-2.4840136489
C	0.8483632498	4.3470486343	0.6943324046
H	1.9013123344	4.6521522459	0.6337628042
H	0.2658213341	5.1746259449	0.2682475220
C	0.6286743052	3.0925809670	-0.1510019711
H	1.1364866970	2.2389790816	0.3120394952
H	-0.4416079056	2.8476932774	-0.1600675584
C	1.1259433492	3.2598134010	-1.5887883787
H	0.7071867464	4.1898488372	-1.9938749940
H	0.7268241700	2.4508979421	-2.2124515964
H	0.8300981577	0.2430575509	-1.2675735069
H	2.1103462745	-4.1038282335	-3.1214205917
H	0.9297556595	-5.6388900536	-1.5627087255
H	-0.9051181869	-3.0517326838	-1.9482040978
H	-5.9081887856	-1.6445983060	0.7459890000
H	-2.5645935267	-3.8076303457	-3.6768622887
H	-6.6100341363	-0.1673453877	2.6039212373
H	-4.9280805788	-3.0570889124	-3.5145039487

1f

0 1

C	2.6004230322	1.4739088640	1.3765301388
N	1.5092273780	2.1234970481	1.1849599744
N	1.3313183977	2.7339488287	-0.0578732397
H	1.3984922060	2.0393483375	-0.8026189873
O	0.3200796825	0.2138461124	-0.9216632493
C	5.6500469478	-0.7754817720	-0.6128129841
C	5.2658774102	-1.2005993871	-1.8161359023
H	5.2806761188	-0.4860604302	-2.6408411147
H	5.6365058586	-1.4687255927	0.2318414588
O	-0.8252190965	0.8263776820	1.3496989542
P	-0.8760549861	0.1356489645	-0.0612903275
O	-1.3545433381	-1.3469687072	0.3621158303
O	-2.1716773321	0.7099873701	-0.8193363510

C	-2.0798440037	-2.1275418388	-0.5275948328
C	-3.4127689473	0.6060158411	-0.2051767933
C	-3.4248977228	-1.8296274108	-0.7811264831
C	-1.4610025414	-3.2449533003	-1.0708385499
C	-4.0696051953	-0.6282441345	-0.2054422458
C	-3.9806515236	1.7520329687	0.3318522793
C	-4.1392864723	-2.7136579528	-1.5965342530
C	-2.1935659355	-4.1065482037	-1.8799569115
H	-0.4148102692	-3.4208668380	-0.8436422882
C	-5.3566482958	-0.6744016005	0.3405863872
C	-5.2603595085	1.6772853306	0.8710115351
C	-3.5367066608	-3.8429124598	-2.1387544741
H	-1.7143240866	-4.9811875473	-2.3075131421
C	-5.9498839276	0.4655935935	0.8706885338
H	-5.8804211289	-1.6257803188	0.3652488431
C	0.1385566675	3.4775556957	-0.1643907800
C	-0.3236336395	4.2501965561	0.9050450425
C	-0.5663897111	3.4728049743	-1.3719758515
C	-1.4764419415	5.0138022122	0.7567159657
H	0.2272286002	4.2451581368	1.8392979006
C	-1.7100316663	4.2502371671	-1.5121704939
H	-0.2274442222	2.8373690453	-2.1859312308
C	-2.1716518264	5.0279614663	-0.4516439575
H	-2.2517514768	4.2333397057	-2.4529088430
C	6.0348902649	0.6363481646	-0.2703136300
H	5.9871038191	1.2656986436	-1.1676956807
H	7.0696499632	0.6703594270	0.0931556470
C	3.6684868162	1.3395980725	0.3232847184
H	3.4037485792	0.4588952235	-0.2854167807
H	3.5991215737	2.2007921800	-0.3506653105
C	5.1110292517	1.2009588722	0.8229406271
H	5.1658688290	0.5345598335	1.6906873311
H	5.4775283919	2.1778894697	1.1574039020
C	4.7913042754	-2.5913338535	-2.1641459889
H	3.9437450214	-2.4993584888	-2.8567092391
H	5.5798261692	-3.1045450306	-2.7314590737
C	4.3642846773	-3.4479342028	-0.9687858991
H	5.1893833940	-3.5118634295	-0.2471242502
C	2.7354691958	0.7542095792	2.6944967604
H	1.8664789928	1.0101255452	3.3097094023
H	3.6255759924	1.1301193390	3.2147633211
C	2.8376709463	-0.7735253905	2.5429113854
H	3.7266489355	-1.0233305949	1.9485046370
H	2.9988984589	-1.2114564042	3.5364587432
C	1.6482234593	-2.9268319677	1.8272379816
C	1.6021819285	-1.3966484766	1.8955353101
H	0.7044552176	-1.0926953092	2.4484980075
H	1.4864606645	-0.9927953409	0.8821001501
H	0.0035624472	1.3817774135	1.4958718344
H	-1.8277034076	5.6119125674	1.5923483843
H	-3.0656219477	5.6331594038	-0.5637229296
H	-3.4090243511	2.6753869055	0.3094608963
H	-5.1787734072	-2.4897485612	-1.8185231866
H	-5.7176838704	2.5656289197	1.2945607181
H	-4.1117116856	-4.5097539939	-2.7727783941
H	-6.9464538664	0.4059611174	1.2958970744

C	3.1107719415	-2.9061724344	-0.2759300615
H	3.1940628867	-1.8165336151	-0.2000916146
H	2.2313915100	-3.0909669072	-0.9093282483
C	2.8845053600	-3.4913176346	1.1164047344
H	2.7967740718	-4.5836529866	1.0495351222
H	3.7789323648	-3.3003962868	1.7264596382
H	0.7403127045	-3.2718751944	1.3159716666
H	1.6041433857	-3.3375550832	2.8443080719
H	4.1844692020	-4.4752978966	-1.3070873348

1g

0 1

C	-2.9537067458	-1.4599518186	-1.2071164864
N	-2.7018686507	-0.2911502235	-0.7297379267
N	-3.1233008640	0.0753649898	0.5608312044
H	-2.2959303947	-0.1088208750	1.1595822418
H	-1.9158740664	0.3248994866	-1.1105076875
O	-0.5193913704	-0.1743662478	1.4422727997
C	-1.5267106506	-3.1210779569	0.1661620032
C	-1.2607122244	-3.7831230217	-0.9605194442
H	-1.9906257345	-4.5154048058	-1.3163682235
H	-0.8424928185	-2.3499534882	0.5261085160
O	-0.4227504775	0.9218280736	-0.9139630253
P	0.2800768321	0.3605906616	0.2947717476
O	1.2816286807	-0.8084380569	-0.2924400759
O	1.3654713036	1.4153906385	0.8912883114
C	2.3441429236	-1.2536159867	0.4656049233
C	2.4536048084	1.7823602138	0.1268075461
C	3.4881233144	-0.4528473586	0.5914787644
C	2.2904047367	-2.5317428975	1.0100076165
C	3.5361181553	0.9024733955	-0.0010838243
C	2.4807371037	3.0557169263	-0.4279340164
C	4.5855009917	-0.9851298354	1.2756385544
C	3.3947618611	-3.0380147385	1.6870740978
H	1.3807986285	-3.1130618992	0.8916528357
C	4.6644552101	1.3534099885	-0.6941882196
C	3.6127415186	3.4800978676	-1.1144384280
H	1.6107912696	3.6910716227	-0.3033452723
C	4.5462083174	-2.2649203337	1.8174966715
H	3.3533150732	-4.0349801929	2.1141604934
C	4.7088748352	2.6293748688	-1.2441277472
H	3.6376736121	4.4743929002	-1.5490283543
H	5.4082252764	-2.6535871922	2.3498964940
H	5.4721717740	-0.3687708691	1.3953831719
H	5.5062205582	0.6771094519	-0.8149442349
H	5.5928436897	2.9553013662	-1.7827238725
C	-3.4511700781	1.4626847938	0.6214170574
C	-4.3587841538	2.0105198246	-0.2841524412
C	-2.8924762280	2.2508985985	1.6276278848
C	-4.7062298516	3.3533690882	-0.1814333900
H	-4.7931706860	1.3825871688	-1.0570929681
C	-3.2629646699	3.5871032934	1.7326730114
H	-2.1567068673	1.8150879147	2.2980907634
C	-4.1664710128	4.1439498976	0.8300045571

H	-5.4095798721	3.7801464895	-0.8897418164
H	-2.8265749398	4.2005327906	2.5146210468
H	-4.4442587849	5.1897596042	0.9108666824
C	-2.9367493623	-3.0816503421	0.7137797917
H	-2.9880442755	-2.4865256724	1.6276479938
H	-3.3394994623	-4.0759533078	0.9305671091
C	-2.3535273275	-1.7949849238	-2.5596580917
H	-2.8580542634	-2.6840652700	-2.9487018112
H	-2.5976302698	-0.9615709829	-3.2288812112
C	-0.2608016589	-3.2940516937	-1.9689203176
H	0.7065675562	-3.0705594348	-1.5076234894
H	-0.0950309875	-4.0441410519	-2.7473112557
C	-0.8072898579	-1.9750295426	-2.5912938870
H	-0.5009865746	-1.9040398369	-3.6395588937
H	-0.3562361489	-1.1207100004	-2.0793506789
C	-3.8055897621	-2.4165313168	-0.4019947915
H	-4.6532675183	-1.8721895743	0.0232327916
H	-4.1717434195	-3.1881131668	-1.0840987998

1h

0 1

C	-2.9445114011	1.6157329680	0.6972010131
N	-2.6612488785	0.3903796631	0.4282232363
N	-2.9749864904	-0.1718201184	-0.8229807015
H	-2.0875676436	-0.1204391095	-1.3615377621
O	-0.3274076578	-0.1946444893	-1.5655919436
C	-1.2570626204	3.0175083501	-0.7891624881
C	-0.9385003475	3.9595230613	0.0988388097
H	-1.6353177644	4.7851678448	0.2752398400
H	-0.5793491014	2.1771966206	-0.9524868084
O	-0.4456945259	-0.8440098073	0.9515511631
P	0.3618904095	-0.5564604581	-0.2858491191
O	1.4147782500	0.6363240581	0.1396670160
O	1.4005713041	-1.7699751722	-0.6029669488
C	2.5362080095	0.8576630536	-0.6334408748
C	2.4158980966	-2.0466608081	0.2888050961
C	3.6291409774	-0.0137985215	-0.5266612907
C	2.5880561040	1.9890053708	-1.4397415485
C	3.5507743220	-1.2252452691	0.3193450847
C	2.3219587220	-3.1865755398	1.0771386693
C	4.7905115381	0.3035401040	-1.2376678677
C	3.7539701686	2.2818830956	-2.1392731303
C	4.6053558627	-1.5992222346	1.1582848350
C	3.3823484840	-3.5360018682	1.9057653950
H	1.4165819173	-3.7806178328	1.0186462725
C	4.8598244467	1.4416773511	-2.0334039708
C	4.5284292542	-2.7440262044	1.9432421978
H	3.3126667981	-4.4263121980	2.5227351129
H	5.7696431240	1.6638899502	-2.5815384537
H	5.6390588655	-0.3722088810	-1.1760614399
H	5.4861492160	-0.9644861044	1.2024797885
H	5.3566358372	-3.0116459345	2.5914639740
C	-3.3668597461	-1.5371045627	-0.6895010680
C	-4.3638947567	-1.8997553864	0.2153232653

C	-2.7783113662	-2.4959543224	-1.5147870166
C	-4.7708706552	-3.2275356619	0.2943394781
H	-4.8210333559	-1.1420675002	0.8457224496
C	-3.2076807359	-3.8163624118	-1.4404189522
C	-4.2007211346	-4.1884820403	-0.5366105780
H	-5.5440892453	-3.5091280561	1.0024584607
C	-2.6279472440	2.9037743825	-1.4176140829
H	-2.6165882623	2.1637252723	-2.2205239870
H	-2.9646210903	3.8554902404	-1.8399722424
C	-2.5343784871	2.1690314880	2.0391798612
H	-2.8608227620	3.2109579110	2.0822589273
H	-3.1383265567	1.6254992821	2.7779689952
C	0.2233039761	3.8481146841	1.0379001059
H	0.9289198445	3.0984538311	0.6636812986
H	0.7580859921	4.7999119514	1.1352121657
C	-0.2663162403	3.3985405519	2.4284164452
H	0.6084224036	3.2931972096	3.0791996871
H	-0.8927810359	4.1823627561	2.8767873147
C	-1.0296596859	2.0641275749	2.4300739813
H	-0.9855295625	1.6477119291	3.4400537166
H	-0.5118720032	1.3410202007	1.7913361216
C	-3.6532242605	2.4556641096	-0.3301796597
H	-4.4617432346	1.8755086412	-0.7820475948
H	-4.0675339041	3.3312127017	0.1750124772
H	1.7105139590	2.6244716333	-1.5084155243
H	3.7952917173	3.1643685592	-2.7698167777
H	-1.9758106934	-2.2002490730	-2.1850331295
H	-2.7480973871	-4.5619685256	-2.0815516422
H	-4.5245581706	-5.2223783159	-0.4764182926
H	-1.9369715926	-0.1708299940	0.9716466403

1i

0 1

C	3.6626212391	-0.6451548399	-0.5228734241
N	2.4654759068	-1.0601470168	-0.3492108170
N	2.1957027175	-1.6767660877	0.9044845796
H	1.8588884248	-0.9283098582	1.5150848213
O	0.4349862588	0.6152989704	1.2555962095
C	4.6211537201	1.6040933767	1.0334440686
C	3.4508367682	2.2336670786	0.9320401531
H	2.5684013419	1.7659077031	1.3726553072
H	5.5144896944	2.0387072884	0.5775979820
O	0.0574937138	-0.3723453559	-1.1170869494
P	-0.5151477969	0.3223761425	0.1622947619
O	-1.2729293863	1.5894634720	-0.4893455122
O	-1.7413807460	-0.5699455937	0.6992773563
C	-2.3540015347	2.1706225483	0.1573779310
C	-2.8032355928	-0.8395456702	-0.1529531034
C	-3.5912447012	1.5141676740	0.1778241511
C	-2.1896969389	3.4431049983	0.6871498177
C	-3.7527639913	0.1583756141	-0.3933473286
C	-2.9146767471	-2.1161255520	-0.6842391050
C	-4.6730257053	2.1932228277	0.7483740469
C	-3.2810965600	4.0952718755	1.2500164286

C	-4.8590340799	-0.1794082930	-1.1801098752
C	-4.0232573453	-2.4242795178	-1.4653423178
H	-2.1333371136	-2.8388090929	-0.4684152792
C	-4.5259131894	3.4702670924	1.2771437960
C	-4.9980194262	-1.4577901932	-1.7083075131
H	-4.1237550385	-3.4193820932	-1.8865858063
H	-5.3796775181	3.9728431270	1.7196537642
H	-5.6362673880	1.6928135579	0.7899050063
H	-5.6015840203	0.5843114725	-1.3932550163
H	-5.8608464713	-1.6961863315	-2.3216941781
C	1.2126547529	-2.6890108739	0.8104841484
C	1.2101323977	-3.5826929485	-0.2635806474
C	0.2697692317	-2.8323812044	1.8331736456
C	0.2813940979	-4.6179253298	-0.3003602617
H	1.9391992109	-3.4622838060	-1.0582415155
C	-0.6450380912	-3.8774995999	1.7926648559
C	-0.6431148926	-4.7798283542	0.7295797817
H	0.2883378301	-5.3087075058	-1.1382645642
C	4.7428712693	0.2175975108	1.6023260882
H	3.8833582776	0.0144743797	2.2494764330
H	5.6454672927	0.1015981584	2.2121439875
C	3.9976927538	0.0752181953	-1.8077225037
C	3.2195710366	3.4691437204	0.0973956838
H	2.2686689956	3.9332957533	0.3813617028
H	4.0072124318	4.2109767078	0.2735412945
C	3.1886108272	3.1022036599	-1.4006412173
H	4.2094469110	2.8292376740	-1.6973231567
H	2.9318774057	3.9893103990	-1.9902270496
C	2.2193470413	1.9425153988	-1.7181900523
H	1.3489856859	2.3122660619	-2.2701944525
C	4.7755774248	-0.8484582883	0.4777214125
H	4.6776724713	-1.8425773410	0.9218155757
H	5.7302924646	-0.7882304383	-0.0568268855
H	-1.2078513693	3.9015816153	0.6397223378
H	-3.1576629599	5.0895440362	1.6667439624
H	0.2475282520	-2.1095061927	2.6444187365
H	-1.3746132820	-3.9763490887	2.5905327679
H	-1.3599897000	-5.5941837094	0.7007246495
H	1.0333399979	-0.6727637139	-0.9807696576
C	2.8480267330	0.7949411241	-2.5214830289
H	1.8261428272	1.5371070504	-0.7816726583
H	3.2416479611	1.1884402540	-3.4671405907
H	2.0697479152	0.0724084679	-2.7894502181
H	4.8043092043	0.7805720285	-1.5805513834
H	4.4449120994	-0.6723881244	-2.4793991757

1j

0 1

C	2.9170159619	1.4799938028	-0.5155990360
N	2.5018846800	0.3304666291	-0.9029547914
N	2.7289794032	-0.7595822342	-0.0590902941
H	2.2978378149	-0.6052133265	0.8516424013
H	0.8993343903	0.1257403439	-1.4199686428
O	0.2133912722	-0.2016128117	1.1852581415

C	1.7577068685	3.1757889859	1.6921426278
C	0.4298975618	3.1287725672	1.5853998965
H	-0.0671755385	2.1665949993	1.7310845786
H	2.2717801645	4.1278345613	1.5325165259
O	-0.1027425463	-0.0006362871	-1.4040472754
P	-0.6746722951	-0.3510825519	0.0166376059
O	-2.0065118325	0.5593830584	0.0202279456
O	-1.2750570424	-1.8392820085	-0.0635973006
C	-3.1111181500	0.1760456676	0.7705179347
C	-2.2261311355	-2.1308602375	-1.0325732048
C	-3.9126801282	-0.8868277650	0.3351663804
C	-3.4337817216	0.9272178841	1.8925616474
C	-3.5428167715	-1.6991390785	-0.8455683661
C	-1.8412907045	-2.8992260711	-2.1213730926
C	-5.0766122918	-1.1572291757	1.0622150041
C	-4.5959586089	0.6356639807	2.5986847408
H	-2.7699240779	1.7315780111	2.1924352960
C	-4.4903883599	-2.0875471410	-1.7984662064
C	-2.8032722237	-3.2696261366	-3.0547911864
H	-0.7993407000	-3.1944957014	-2.2058129966
C	-5.4220160809	-0.4040347851	2.1784905652
H	-4.8526291359	1.2196905029	3.4763888472
C	-4.1278856383	-2.8667324198	-2.8912227615
H	-2.5173973775	-3.8709801140	-3.9117212524
H	-6.3275624735	-0.6380997351	2.7284167994
H	-5.7015808559	-1.9891370652	0.7503641445
H	-5.5167884009	-1.7512021733	-1.6825669733
H	-4.8777419577	-3.1510339783	-3.6222183481
C	2.3181502369	-1.9929083157	-0.6064086991
C	2.5051852843	-2.2717993916	-1.9636996973
C	1.7540031310	-2.9618133470	0.2290769833
C	2.1369090817	-3.5138675694	-2.4698429125
H	2.9398555384	-1.5133647902	-2.6057760997
C	1.3981504365	-4.2022419063	-0.2869414844
H	1.5686882343	-2.7241685488	1.2730443894
C	1.5898866714	-4.4891385348	-1.6370977998
H	2.2889070389	-3.7230279861	-3.5246823073
H	0.9534198798	-4.9429692709	0.3703814022
H	1.3106965940	-5.4587934753	-2.0368341494
C	2.6293490344	1.9809588623	1.9579218385
H	1.9885292217	1.1085870271	2.1224899562
H	3.2239733689	2.1326168856	2.8674284189
C	3.6200854869	1.7175651329	0.7954040583
H	4.2414935580	0.8459469598	1.0279026353
H	4.2775941403	2.5879520944	0.6884809799
C	-0.4516232385	4.2914692030	1.2270425507
H	-1.0387533251	4.5960350934	2.1046017879
H	0.1659743742	5.1556146066	0.9506353583
C	-1.4262031858	3.9312753201	0.0941235338
H	-2.0744264282	3.1172963536	0.4391289847
C	2.6823935811	2.6677711071	-1.4092225616
H	3.6552688918	3.1622037690	-1.5486768519
H	2.0748691520	3.3862192602	-0.8449579047
C	2.0623052580	2.3941514514	-2.7777031889
H	1.1270790653	1.8324629667	-2.6763191343
H	2.7449040621	1.7487616895	-3.3418761063

C	0.3853911789	4.2866456756	-3.3899141795
C	1.7924546619	3.6962592142	-3.5500895629
H	2.5352524111	4.4516845879	-3.2566875012
H	1.9525371844	3.5189340444	-4.6196269559
C	-0.7487245648	3.4888862082	-1.2050576135
H	-0.0343575311	2.6860283666	-0.9805279947
H	-1.5043225179	3.0350820997	-1.8587678479
C	-0.0769555551	4.6390350432	-1.9649252887
H	-0.8115135544	5.4526741855	-2.0346655674
H	0.7647374766	5.0512048504	-1.3899902070
H	-0.3415899087	3.5888540844	-3.8276209387
H	0.3430993243	5.1927904116	-4.0060929799
H	-2.0804258179	4.7894363849	-0.1075366975

1k

0 1

C	3.2354971049	1.0650672724	-0.8195188806
N	2.2866819045	0.2086852286	-0.8346151604
N	2.4071816832	-0.8999085771	0.0461732195
H	1.9215036864	-0.6372763915	0.9091014800
O	0.0779674011	0.1252949489	1.4210245429
C	5.4842611665	-1.3632579855	-3.5829239680
C	5.8063552036	-0.5162566688	-4.5598590164
H	6.7658792499	0.0055386937	-4.5170197257
H	4.5197272395	-1.8755968965	-3.6358428050
O	-0.3208947827	0.3394059182	-1.1396024040
P	-0.8703426037	0.0294140451	0.2953859131
O	-2.1535356931	1.0046494189	0.3332814098
O	-1.5525008410	-1.4292526697	0.2566912387
C	-3.2553931168	0.6965492800	1.1173599446
C	-2.5506832432	-1.6837236727	-0.6711805312
C	-4.1226871683	-0.3344571176	0.7336426868
C	-3.5137638977	1.4997149693	2.2198592918
C	-3.8371500580	-1.1859715212	-0.4427062088
C	-2.2457579139	-2.4832978841	-1.7630583775
C	-5.2764259674	-0.5267613410	1.5012277353
C	-4.6669716313	1.2861014006	2.9665335585
H	-2.8069759736	2.2844129623	2.4662050084
C	-4.8376796571	-1.5401546419	-1.3541733534
C	-3.2585723995	-2.8188411176	-2.6546889263
H	-1.2234429829	-2.8294615230	-1.8829032216
C	-5.5520132028	0.2731371948	2.6040295499
H	-4.8722708402	1.9104833258	3.8299768756
C	-4.5553347780	-2.3505351530	-2.4479033952
H	-3.0340116403	-3.4444217715	-3.5126606325
H	-6.4509678094	0.0989223409	3.1861493131
H	-5.9509497100	-1.3339675590	1.2301870853
H	-5.8412282693	-1.1515775313	-1.2058058011
H	-5.3453229254	-2.6072418391	-3.1461852837
C	1.8418740108	-2.0768347400	-0.4904733570
C	1.9253710375	-2.3589139565	-1.8576281508
C	1.2475748368	-3.0059028373	0.3710411908
C	1.4474665203	-3.5725530471	-2.3462501462
H	2.3575573687	-1.6229643987	-2.5288168932

C	0.7766053893	-4.2129718367	-0.1276226991
H	1.1406678415	-2.7618183550	1.4244907308
C	0.8812741732	-4.5103774566	-1.4868639641
H	1.5219162765	-3.7827244453	-3.4093320491
H	0.3120620664	-4.9223299797	0.5502304875
H	0.5128020675	-5.4559838696	-1.8711161870
C	4.5185853693	0.9396327166	-0.0385641134
H	4.8004310047	1.9407363697	0.3053604944
H	4.3879241539	0.2970402746	0.8336138472
C	4.9138922029	-0.1953033417	-5.7251690908
H	5.3663301560	-0.5572548124	-6.6577269273
H	3.9663898579	-0.7350707059	-5.6071096298
C	4.6465235836	1.3115880930	-5.8687482586
H	3.9166001302	1.4638115438	-6.6736915928
H	5.5704580882	1.8101963209	-6.1881199959
C	4.1413241887	1.9818100027	-4.5883069759
H	4.9450791312	1.9614390431	-3.8408902639
H	3.9429965404	3.0416212179	-4.7983535264
C	2.8782320982	1.3412194679	-4.0077514695
H	2.1518980385	1.1834663176	-4.8158868584
H	3.1222739117	0.3471705333	-3.6075606418
C	3.0720222814	2.3196528713	-1.6484055453
H	4.0708344895	2.6974699614	-1.8969778462
H	2.6323087997	3.0717899846	-0.9781433850
C	2.2200420924	2.1867396107	-2.9130788569
H	1.2400643514	1.7744190300	-2.6556465047
H	2.0361326413	3.1946309899	-3.3048854297
C	5.5470668371	-1.1515731584	-1.1020166817
H	4.5005570826	-1.4730155537	-1.1639250314
H	5.9540992355	-1.6190539098	-0.1980099082
C	6.2902525981	-1.6539787410	-2.3508213011
H	6.4522947083	-2.7349501292	-2.2627792613
H	7.2790759889	-1.1827280875	-2.4221522073
C	5.6186478183	0.3700277317	-0.9583217059
H	5.5365092447	0.8314472291	-1.9526805013
H	6.6012337287	0.6550938114	-0.5654503515
H	0.7032705241	0.3299851035	-1.1745406039

2a

0 1

C	3.5989777567	-0.3184442862	0.8196738944
N	2.5487069897	0.3062966675	0.4181461952
N	2.4664580206	0.8721694278	-0.8727255591
H	1.8052391027	0.2496250216	-1.3641626444
O	0.2366169191	-0.7982874316	-1.1968606094
C	3.2121355438	-2.5932727177	-0.6553726290
C	3.0358489085	-3.1374919818	0.5518213577
H	3.8627091235	-3.6716957253	1.0282271801
H	2.3673180791	-2.0722259939	-1.1101342164
O	0.0950467593	0.2197755589	1.1751775275
P	-0.6011209639	-0.3123439996	-0.0570854662
O	-1.6346151009	-1.4527705720	0.4881851732
O	-1.6476346759	0.7854981839	-0.6567114304
C	-2.7329521777	-1.8204864709	-0.2595357977

C	-2.6989544912	1.2064193858	0.1303956574
C	-3.8437003173	-0.9681603809	-0.3394377837
C	-2.7434352942	-3.0816766568	-0.8428791227
C	-3.8190975728	0.3783551157	0.2746855478
C	-2.6469321715	2.4746072317	0.6948921769
C	-4.9766539277	-1.4359820834	-1.0131820395
C	-3.8808863312	-3.5227395236	-1.5096200856
H	-1.8544980186	-3.6968181206	-0.7544186980
C	-4.9088574929	0.8775671535	0.9953176678
C	-3.7426260116	2.9471015685	1.4087474202
H	-1.7461727224	3.0643429838	0.5559612449
C	-5.0025083833	-2.7002436719	-1.5908773561
H	-3.8900082380	-4.5074653421	-1.9661127220
C	-4.8766233306	2.1497255622	1.5554708409
H	-3.7099723983	3.9368941635	1.8532248751
H	-5.8914953395	-3.0378104375	-2.1137802377
H	-5.8387226012	-0.7799730879	-1.0971382092
H	-5.7805567332	0.2430537101	1.1298645428
H	-5.7311330959	2.5143162591	2.1164678700
C	1.9057762560	2.1860530271	-0.8198843497
C	0.8340429345	2.5061320035	-1.6528709526
C	2.4524086327	3.1498082414	0.0270781746
C	0.3202023216	3.7989649078	-1.6418629775
H	0.3867022747	1.7340951940	-2.2728316273
C	1.9210167193	4.4344960754	0.0417142275
H	3.2893198004	2.8890083030	0.6691014356
C	0.8573141381	4.7662613987	-0.7951995989
H	-0.5203237841	4.0412436167	-2.2847193037
H	2.3446335543	5.1814340782	0.7059789562
H	0.4474490015	5.7710158236	-0.7837194061
C	4.5683568894	-2.4602318234	-1.3007958325
H	4.4812296242	-1.9630436610	-2.2718090324
H	5.0355972423	-3.4358461256	-1.4782819966
C	3.5363692220	-0.9596245729	2.1833686788
H	3.6362286258	-0.1512643710	2.9202121916
H	4.4250885817	-1.5870424176	2.2914100938
C	1.8516540586	-2.8034892413	1.4053197206
H	1.0739783210	-2.3723444083	0.7680171041
H	1.4206694040	-3.6823437076	1.8949017930
C	2.2603800007	-1.7735815866	2.5019821002
H	2.4620728930	-2.2899106578	3.4457359077
H	1.4154032772	-1.1020506441	2.6789643184
C	4.8891974120	-0.2755920280	0.0426086408
H	4.7408417000	0.3579012817	-0.8336574261
H	5.6122077230	0.2201938822	0.7060303733
C	5.4813836725	-1.6380290693	-0.3705302849
H	6.4451069987	-1.4431714567	-0.8531971420
H	5.6957256240	-2.2301932533	0.5264946390
H	1.5823990676	0.2823426813	0.9291570100

2b

0 1

C	-2.9163239182	-1.5672779637	-1.2206706874
N	-2.4622701106	-0.1545570549	-1.1435348448

N	-2.3775754369	0.1883592061	0.2676609649
H	-1.2916525804	0.2448181796	0.5833152594
O	0.0426905945	0.4385927589	1.0180019875
C	-3.0166634833	-0.9618517185	1.0319865440
C	-2.5912600281	-2.1532023687	0.1718564849
H	-3.2512583419	-3.0056346968	0.3718514005
H	-2.5730642007	-0.9626966371	2.0321916367
O	0.2995398067	0.6183535007	-1.5429106801
P	0.9409815648	0.4721038651	-0.2080762228
O	1.8439724723	-0.8957263974	-0.2494754049
O	2.0767853030	1.5860504700	0.1192829980
C	2.7745493655	-1.1381037510	0.7381902237
C	3.2695784213	1.5768350013	-0.5763007578
C	3.9863867989	-0.4341423167	0.7388716137
C	2.5210406487	-2.1461928876	1.6605202888
C	4.2485364008	0.6264204009	-0.2592275444
C	3.4994758864	2.5747466923	-1.5147343035
C	4.9440361012	-0.7847907071	1.6961181739
C	3.4882857321	-2.4761934451	2.6031711293
H	1.5661415352	-2.6603129513	1.6161806505
C	5.4829929522	0.7210160333	-0.9102988674
C	4.7339920865	2.6462618649	-2.1501605111
H	2.7033377977	3.2810038138	-1.7228585570
C	4.7040129832	-1.7958364240	2.6195702257
H	3.2920084685	-3.2633510947	3.3243169063
C	5.7295796559	1.7205527615	-1.8442696246
H	4.9170282148	3.4240399231	-2.8846801900
H	5.4603441848	-2.0458488852	3.3564778190
H	5.8814671420	-0.2361027383	1.7190318018
H	6.2467207822	-0.0190414276	-0.6879236004
H	6.6929417041	1.7698052403	-2.3414009604
C	-2.8841780391	1.5248331589	0.5907899051
C	-3.4722229387	2.3350042900	-0.3700994839
C	-2.6808871469	1.9737976563	1.8935593487
C	-3.9093599771	3.6054706983	0.0000454977
H	-3.5627562006	1.9704645361	-1.3860285866
C	-3.1254116156	3.2416938322	2.2495799770
H	-2.1532827771	1.3495506276	2.6086651772
C	-3.7472428666	4.0570312152	1.3057975166
H	-4.3708554221	4.2460707626	-0.7445130512
H	-2.9696043239	3.5979140404	3.2625194729
H	-4.0873212652	5.0489935762	1.5849640726
C	-4.5397742920	-0.8382857783	1.0692840788
H	-4.8423297898	0.0104162693	1.6912349458
H	-4.9113758149	-1.7441494906	1.5624028694
C	-2.2248122954	-2.2826804889	-2.3813169524
H	-2.7800603918	-3.2115941833	-2.5687101658
H	-2.3311149876	-1.6666729802	-3.2829650162
C	-1.1358330692	-2.5887943884	0.3718058833
H	-0.4882144204	-1.7189528723	0.5113039016
H	-1.0729589133	-3.1635307830	1.3045163861
C	-0.6133492139	-3.4074866304	-0.8088052927
H	0.4376566720	-3.6631015471	-0.6377497974
H	-1.1705933639	-4.3525661884	-0.8848007755
C	-0.7559342657	-2.6198551728	-2.1115495961
H	-0.3635464731	-3.2010540621	-2.9524153063

H	-0.1520836355	-1.7059589934	-2.0523275623
C	-4.4452438586	-1.5744760467	-1.3914373884
H	-4.6966415466	-1.2113883768	-2.3946510175
H	-4.7921406122	-2.6133950957	-1.3249543604
C	-5.1395210901	-0.6978119588	-0.3406485259
H	-5.0511977650	0.3465586521	-0.6509368222
H	-6.2103199948	-0.9208937160	-0.3061542684
H	-1.5082218085	-0.0182828083	-1.5079424153

2c

0 1

C	-3.0575585273	-1.2454087931	-0.9168813488
N	-2.4561092203	0.1036889452	-1.0959852533
N	-2.2422720054	0.6489364632	0.2310775273
H	-1.1611365690	0.6225206454	0.4854797914
H	-1.5050418175	0.0211599972	-1.4845434008
O	0.2223932821	0.6633709763	0.9486850327
C	-2.8895621530	-0.3189577341	1.2134347050
C	-2.6368220221	-1.6585851120	0.5139482554
H	-3.3416334441	-2.3932230524	0.9214793211
H	-2.3393115843	-0.1938188672	2.1499802487
O	0.4053984256	-0.0767273450	-1.5114141764
P	1.0889704209	0.2620103319	-0.2304213770
O	2.0306091018	-1.0114964554	0.1755669534
O	2.2063048161	1.4368497464	-0.3775148144
C	3.0213959512	-0.8682223956	1.1227357102
C	3.3560876307	1.1960767039	-1.1016749858
C	4.2100684263	-0.2027327199	0.7927382208
C	2.8486155375	-1.4710726268	2.3628007488
C	4.3824582384	0.4308261905	-0.5332369246
C	3.4984833688	1.7953078019	-2.3467051242
C	5.2293631694	-0.1741474717	1.7499762161
C	3.8758663016	-1.4282537847	3.2985903624
H	1.9086195555	-1.9734975836	2.5651197169
C	5.5739880371	0.2997127202	-1.2540739918
C	4.6915529023	1.6482461624	-3.0452924834
H	2.6681989480	2.3711835360	-2.7403518957
C	5.0707970861	-0.7810722904	2.9905780572
H	3.7426402927	-1.8992617091	4.2674174016
C	5.7332587398	0.9030254078	-2.4962441572
H	4.8064232164	2.1143901255	-4.0187190611
H	5.8739851054	-0.7402906959	3.7191616854
H	6.1498946037	0.3522982010	1.5134803591
H	6.3732538511	-0.3044207881	-0.8337309736
H	6.6643384470	0.7819749885	-3.0404117597
C	-2.6307947970	2.0531583575	0.3588157635
C	-3.5624367564	2.6240461586	-0.4988004981
C	-2.0332408816	2.7820706864	1.3821147394
C	-3.9208215274	3.9552392297	-0.3062493958
H	-3.9717019424	2.0308311468	-1.3080456742
C	-2.4024999022	4.1120944739	1.5617177599
H	-1.2665421782	2.3204633994	1.9974046978
C	-3.3479815347	4.6978974819	0.7237484351
H	-4.6460993525	4.4143813399	-0.9703336475

H	-1.9371926080	4.6933164736	2.3509837490
H	-3.6284750349	5.7368154389	0.8643749891
C	-4.3942957885	-0.0643218045	1.3859882469
H	-4.6023878615	1.0051893373	1.3003823992
H	-4.6635634688	-0.3428210917	2.4097305964
C	-2.5402598119	-2.1450481170	-2.0447746125
H	-3.1072051007	-1.8984933737	-2.9504253128
H	-1.4923159659	-1.8953802233	-2.2562607647
C	-2.6368544211	-3.6488175079	-1.7444111213
H	-3.5459090826	-3.8682361032	-1.1651908365
H	-2.7465951922	-4.1866155594	-2.6920645598
C	-4.5989061782	-1.1097342729	-0.9742235596
H	-4.8229845958	-0.2916572363	-1.6662575573
H	-5.0250814004	-2.0197911612	-1.4128916294
C	-5.2543819427	-0.8801019788	0.3999149491
H	-6.2280122087	-0.3973205840	0.2761937721
H	-5.4619202902	-1.8559199954	0.8511285267
C	-1.4007906727	-4.2051190112	-1.0236843324
H	-0.5131188725	-3.9155269577	-1.6011119216
H	-1.4404809659	-5.3006193436	-1.0439541280
C	-1.2228271775	-2.2352009162	0.6318709104
H	-0.5553339804	-1.7724638080	-0.1014048427
H	-0.8151689925	-1.9926740100	1.6203601149
C	-1.2090991994	-3.7545665135	0.4273164595
H	-1.9861084732	-4.2086237883	1.0591300682
H	-0.2507909539	-4.1493326844	0.7828156314

2d

0 1

C	3.0646807840	1.4898399809	-0.8738642842
N	2.5227187776	0.1200612327	-1.0785965363
N	2.3344063730	-0.4486625017	0.2417561661
H	1.2530840062	-0.5677311177	0.4691057533
O	-0.1034531908	-0.7796407327	0.9726314365
C	2.8013410172	0.5926243810	1.2631618744
C	2.4709230714	1.8918884417	0.5100749095
H	3.0378787476	2.7165804081	0.9589839170
H	2.1678403070	0.4301131689	2.1389070142
O	-0.3655470740	-0.1744447146	-1.5134998107
P	-1.0115962186	-0.4927482505	-0.2082673085
O	-2.0240110124	0.7422477599	0.1499308108
O	-2.0555262045	-1.7385364102	-0.2766921507
C	-2.9866239723	0.5915572788	1.1244494682
C	-3.2334799413	-1.6064398988	-0.9836456553
C	-4.1355120251	-0.1659248926	0.8581311399
C	-2.8321549734	1.2762795585	2.3238872162
C	-4.2939420480	-0.8786289555	-0.4289104778
C	-3.3640461217	-2.2766933300	-2.1933717989
C	-5.1326062674	-0.2057383138	1.8381633978
C	-3.8368804812	1.2209636817	3.2831897801
H	-1.9253209112	1.8522692442	2.4764297564
C	-5.5063809641	-0.8593000527	-1.1261512088
C	-4.5786030268	-2.2403624570	-2.8689485250

H	-2.5074630013	-2.8194680552	-2.5776022796
C	-4.9913581736	0.4799862291	3.0391464278
H	-3.7176790485	1.7556480557	4.2202745410
C	-5.6535448306	-1.5343308893	-2.3323882067
H	-4.6843561033	-2.7621735059	-3.8147627485
C	2.8947013363	-1.7915972510	0.3948892413
C	3.8658281710	-2.2772447432	-0.4699827927
C	2.4122282267	-2.5535755967	1.4555256387
C	4.3863760998	-3.5493899797	-0.2449073871
H	4.1767249830	-1.6737897995	-1.3137927404
C	2.9430782478	-3.8219531176	1.6673265763
H	1.6098313450	-2.1674436049	2.0770505880
C	3.9339457411	-4.3188405423	0.8236778678
H	5.1442919387	-3.9413067389	-0.9155901844
H	2.5692238547	-4.4268628498	2.4868548844
H	4.3419389228	-5.3106069631	0.9900733034
C	4.2890666901	0.4784675180	1.6096030966
H	4.6012916183	-0.5691503815	1.5856862138
H	4.4176573019	0.8118790034	2.6442803568
C	4.6175421763	1.3786427936	-0.7587473840
H	4.9170493596	0.4813403817	-1.3092766925
H	5.0791490206	2.2272875024	-1.2746017444
C	5.1535161310	1.3379110652	0.6773612444
H	6.1862003395	0.9771765003	0.6811739433
H	5.1897415314	2.3595160455	1.0703124761
C	0.9696444690	2.2199285312	0.5519602239
H	0.4479946945	1.7102327790	-0.2581203188
H	0.5427312762	1.8042488914	1.4723949838
C	0.6507671455	3.7152094868	0.5078069692
H	0.9757997945	4.1747572769	1.4512567880
H	-0.4397724576	3.8293346716	0.4627006080
C	1.2886250928	4.4827131281	-0.6480217554
H	2.3829725939	4.4285473915	-0.5612970086
H	1.0406576327	5.5447011625	-0.5336184260
C	0.8627370662	4.0452376731	-2.0523130711
H	-0.2285999149	4.1187237281	-2.1361802939
H	1.2744267456	4.7783193797	-2.7581591067
C	2.7592061285	2.3017187195	-2.1472630607
H	3.3450568330	3.2270370203	-2.1014186442
H	3.2089925162	1.7116244496	-2.9565543065
C	1.3067703532	2.6446017926	-2.5175010741
H	0.6087720308	1.8707616726	-2.1859125842
H	1.2330238892	2.6317697730	-3.6104238818
H	-6.0211081600	-0.8025825445	1.6517266207
H	-6.3331929655	-0.2852187242	-0.7171872085
H	-5.7763770829	0.4288426815	3.7866203278
H	-6.6018345508	-1.4996263707	-2.8588779185
H	1.5737653405	0.1447228457	-1.4785919860

2e

0 1

C	2.8866395254	1.3105114005	-0.8231069581
N	2.4298663000	-0.0845903773	-1.0540626826
N	2.3063392781	-0.7049204008	0.2523469421

H	1.2265856950	-0.8467609287	0.4805448250
O	-0.1653417215	-1.1611820908	0.7914152627
C	2.8421799460	0.2992348062	1.2742843883
C	2.3945666485	1.6173979185	0.6255131673
H	2.9681260491	2.4420224299	1.0642255014
H	2.3170488367	0.0700505145	2.2052816120
O	-0.4267786258	-0.1146219057	-1.5565513909
P	-1.0629100762	-0.6327586360	-0.3123446550
O	-2.0197339996	0.5594336469	0.2778559882
O	-2.1621893751	-1.8038090117	-0.5748732555
C	-2.9671510037	0.2727207889	1.2362444675
C	-3.3437193106	-1.4999828775	-1.2196687984
C	-4.1530305429	-0.3781714734	0.8692741084
C	-2.7580126751	0.7191427608	2.5356617410
C	-4.3635818770	-0.8420356640	-0.5198967301
C	-3.5220633860	-1.9363336576	-2.5263699551
C	-5.1323595945	-0.5541085498	1.8521052858
C	-3.7457905818	0.5314128039	3.4959838689
H	-1.8219913483	1.2174219346	2.7671132598
C	-5.5850211220	-0.6505140280	-1.1739154176
C	-4.7444576467	-1.7315792259	-3.1563425426
C	-4.9374892960	-0.1038310823	3.1529070299
H	-3.5842091999	0.8801628825	4.5111269602
C	-5.7798598430	-1.0918352702	-2.4776214352
H	-6.3801083439	-0.1273999317	-0.6497836746
C	2.8733451093	-2.0514463182	0.3213906873
C	3.8573611040	-2.4702759671	-0.5644104430
C	2.3848634653	-2.8860699822	1.3214929728
C	4.3834743713	-3.7513793033	-0.4221173407
H	4.1756982230	-1.8045832102	-1.3578169877
C	2.9206310585	-4.1638437041	1.4511458991
H	1.5715184790	-2.5488377201	1.9572640985
C	3.9226869455	-4.5954217024	0.5856446048
H	2.5411207165	-4.8267140091	2.2217408298
C	4.3650819787	0.2255780376	1.4485132565
H	4.7100887545	-0.8017831651	1.3040802543
H	4.5942709641	0.4791042158	2.4884330034
C	4.4407448156	1.3218753752	-0.8675876081
H	4.7435436283	0.5007272080	-1.5250524746
H	4.7936060826	2.2452241515	-1.3385401528
C	5.1029532091	1.1947377419	0.5110529692
H	6.1466184871	0.8856547027	0.4018806956
H	5.1302933979	2.1841960714	0.9804616172
C	0.8845598773	1.8784612771	0.7957010287
H	0.3643162322	1.7253449039	-0.1531023897
H	0.4615794799	1.1211251036	1.4648869185
C	0.5123798650	3.2508439358	1.3686648438
H	0.9218905564	3.3431092853	2.3839388863
H	-0.5805661609	3.2718716141	1.4687767083
C	2.3146083172	2.1675461426	-1.9561693841
H	1.2259615967	2.0471186354	-1.9818876785
H	2.6799345942	1.7355595346	-2.8958557102
C	2.7089578890	3.6551991105	-1.8655238514
H	3.2390206491	3.8374265064	-0.9225542031
H	3.4331621921	3.8971478050	-2.6510573025
C	0.9564069978	4.4617580605	0.5408389387

H	2.0515236236	4.5221452732	0.5538250752
H	0.6148340941	5.3694813679	1.0500366253
C	0.4397841802	4.4467812309	-0.9126678240
H	-0.0958881095	3.5106998132	-1.1070582098
H	-0.3067336156	5.2362640129	-1.0489429863
C	1.5338791200	4.6344371901	-1.9722611423
H	1.9331533933	5.6544692852	-1.9009962090
H	1.0809191090	4.5469475849	-2.9675850674
H	1.4684882575	-0.0829638281	-1.4308171352
H	5.1517659144	-4.0920873222	-1.1087362843
H	4.3338864555	-5.5946425293	0.6867287740
H	-2.6958743740	-2.4347660250	-3.0213141689
H	-6.0499186760	-1.0710517660	1.5851135224
H	-4.8872627917	-2.0704715813	-4.1775268058
H	-5.7097478858	-0.2572199312	3.8997087676
H	-6.7339562804	-0.9261798883	-2.9674495307

2f

0 1

C	2.9156053210	1.1742860787	-0.5578007802
N	2.4072704714	-0.1641383663	-0.9656028805
N	2.2343218821	-0.9389698655	0.2489938508
H	1.1425613228	-1.0670140471	0.4428299453
O	-0.2549472140	-1.3691786473	0.6916822322
C	2.7897200426	-0.0950244666	1.3960731751
C	2.4040368951	1.3115842802	0.9115180042
H	2.9977758925	2.0517210895	1.4607148040
H	2.2382762174	-0.4150029291	2.2840875055
O	-0.4435133765	-0.0072329576	-1.4958225246
P	-1.1177158380	-0.6726620081	-0.3453474148
O	-2.0594916336	0.4502171614	0.3868358809
O	-2.2373205694	-1.7704711058	-0.7833879943
C	-3.0246062297	0.0548966341	1.2872443811
C	-3.4026350118	-1.3530320910	-1.3935637567
C	-4.2178273672	-0.5129909596	0.8213615759
C	-2.8241677156	0.3138121661	2.6377680328
C	-4.4179158947	-0.7744342619	-0.6214377665
C	-3.5717731721	-1.6009967155	-2.7497952927
C	-5.2134428839	-0.8028942073	1.7596811949
C	-3.8283874736	0.0166872354	3.5521965710
H	-1.8820211018	0.7560913479	2.9453494933
C	-5.6245908702	-0.4643508355	-1.2565773441
C	-4.7799045046	-1.2819565924	-3.3594872677
C	-5.0272496527	-0.5403040062	3.1121243812
H	-3.6741584658	0.2194966391	4.6074642973
C	-5.8102017924	-0.7166171204	-2.6109180298
H	-6.4153197296	0.0010990835	-0.6743644237
C	2.7470711295	-2.3051913788	0.1535996598
C	2.2229802839	-3.2340898306	1.0469102530
C	3.7140882704	-2.6541972003	-0.7796861143
C	2.7045452307	-4.5391452171	1.0190928716
H	1.4241243857	-2.9423847073	1.7225507041
C	4.1858098844	-3.9640040148	-0.7955265140
H	4.0604622607	-1.9110222919	-1.4880678912

C	3.6887550481	-4.9040264286	0.1038076914
H	4.9400850127	-4.2514917611	-1.5211403668
C	4.3040681831	-0.2568046630	1.5804736821
H	4.6086645587	-1.2706534661	1.3069564514
H	4.5250793714	-0.1507725620	2.6473772524
C	4.4686016918	1.1259541699	-0.5740362587
H	4.7562125866	0.3878706509	-1.3298419346
H	4.8709565593	2.0873682078	-0.9063313077
C	5.0961786893	0.7924992198	0.7867061512
H	6.1292642621	0.4583462948	0.6530787272
H	5.1523040286	1.7112525500	1.3806633343
C	0.9003653770	1.6021036322	1.1062815961
H	0.3770705833	1.5986178126	0.1465906122
H	0.4463141772	0.7807500587	1.6712251497
C	0.6050064695	2.9075082576	1.8519971041
H	-0.4805986254	2.9817596510	1.9973270788
C	2.3709626021	2.1595457173	-1.5990659192
H	1.2835420223	2.2078597162	-1.4943588367
H	2.5389237666	1.7023731188	-2.5836464176
C	2.9825113276	3.5723442882	-1.6179746531
H	3.8724559384	3.5565526359	-2.2567560786
H	3.3458106036	3.8623040531	-0.6250222770
C	1.1683156859	5.3882113302	-1.1095484303
C	2.0076522972	4.6383874799	-2.1550782700
H	2.5727899285	5.3874486904	-2.7216532669
H	1.3335367646	4.1592919947	-2.8778897218
H	1.4530552004	-0.0730275830	-1.3507687650
H	2.2971346898	-5.2740486184	1.7057248430
H	4.0575089440	-5.9245770597	0.0812957739
H	-2.7493932863	-2.0463810166	-3.2988766950
H	-6.1368087852	-1.2597307684	1.4143384482
H	-4.9152893421	-1.4739156377	-4.4192771311
H	-5.8118773259	-0.7785734808	3.8230308264
H	-6.7532418933	-0.4614053170	-3.0835097922
C	1.1123250555	4.1586307467	1.1376643331
H	1.0602144272	5.0180624178	1.8178866276
H	2.1781774075	4.0312263751	0.9195777871
C	0.3495936107	4.5106268262	-0.1479478790
H	0.0150771622	3.6039658972	-0.6654562663
H	-0.5703491460	5.0346945342	0.1362637073
H	0.4977585340	6.0629598358	-1.6537849627
H	1.8306777623	6.0352460686	-0.5165669782
H	1.0539730819	2.8557142390	2.8533862122

2g

0 1

C	-3.0985936815	-1.6174493467	-1.8463902390
N	-2.6062108076	-0.2742117465	-1.4425404007
N	-2.5806441742	-0.3070766556	0.0157131894
H	-1.5291504146	-0.3209720053	0.3853017382
H	-1.6285515208	-0.0933764647	-1.7194691276
O	-0.1888268377	-0.2201385241	0.9487372834
C	-3.2256971627	-1.6349359920	0.3688938979
C	-2.6336925188	-2.5408149408	-0.7090980009

H	-3.2033898961	-3.4768529240	-0.7301156952
H	-3.0402775539	-1.8431069130	1.4232685954
O	0.1675534481	0.4486189282	-1.5152274581
P	0.7525418151	0.1891792245	-0.1703079104
O	1.9121649626	-0.9506486678	-0.3453568724
O	1.6236154094	1.4299730847	0.4205254691
C	2.8137342953	-1.1723122381	0.6736597028
C	2.8278307932	1.7631939831	-0.1660267422
C	3.8602384679	-0.2643145642	0.8837191485
C	2.7091375803	-2.3428912448	1.4149782542
C	3.9595036177	0.9756595002	0.0824653110
C	2.9026768369	2.9279716249	-0.9193010626
C	4.8119328244	-0.5789945116	1.8589346324
C	3.6666094594	-2.6333707143	2.3803341413
H	1.8782585099	-3.0101264142	1.2116517555
C	5.1824448558	1.4076684063	-0.4408799208
C	4.1295624841	3.3353182265	-1.4311352848
H	1.9935048124	3.4957486255	-1.0836659072
C	4.7225484303	-1.7513266318	2.6008628774
H	3.5872293237	-3.5474163276	2.9603951897
C	5.2727388681	2.5765199519	-1.1879895868
H	4.1916444695	4.2442935027	-2.0210271333
H	5.4695668580	-1.9711893346	3.3566808407
H	5.6197353260	0.1237541244	2.0434661257
H	6.0658255006	0.7984306282	-0.2707207083
H	6.2313382956	2.8883210154	-1.5898962413
C	-3.1886047639	0.8658981294	0.6464202294
C	-4.0857650362	1.6739471548	-0.0394899817
C	-2.8253120878	1.1267241507	1.9637930929
C	-4.6550021979	2.7553405435	0.6276453728
H	-4.3020858385	1.4627647009	-1.0802125068
C	-3.4029374923	2.2113619891	2.6167756173
H	-2.0725380706	0.5120900718	2.4486323230
C	-4.3210017786	3.0216715898	1.9533382293
H	-5.3559599249	3.3968191773	0.1033416692
H	-3.1210421046	2.4300705944	3.6414095405
H	-4.7644720414	3.8701457757	2.4644303823
C	-4.7028501033	-1.5756098934	-0.0751480996
H	-5.2192590406	-0.7006917022	0.3243104612
H	-5.2185160233	-2.4621664826	0.3034315964
C	-2.3535386189	-2.1131104083	-3.0702884531
H	-2.8801238645	-2.9863329755	-3.4709406007
H	-2.2903976138	-1.3664316044	-3.8671783080
C	-1.1468918188	-2.8001067072	-0.9797799121
H	-0.4963953305	-2.1721056858	-0.3687806600
H	-0.8934527675	-3.8348169768	-0.7368710439
C	-0.9585481928	-2.5045400656	-2.5036386810
H	-0.5512512107	-3.3681600907	-3.0344624553
H	-0.2494620615	-1.6821820599	-2.6287666026
C	-4.6151766747	-1.5867544370	-1.6425634720
H	-5.0710569762	-0.7024323338	-2.0928438555
H	-5.0909150427	-2.4782891194	-2.0618067430

2h

0 1

C	-3.4727864733	-1.4796196726	-0.9147640362
N	-2.6572389130	-0.2461458737	-1.0571436360
N	-2.4518273093	0.2160567021	0.3124382732
H	-1.3706519297	0.1301784857	0.5988648639
O	0.0077717712	0.2001186706	1.0125766336
C	-3.2615180679	-0.7447383774	1.1729728738
C	-3.0176750378	-2.0665959717	0.4400288874
H	-3.7520166058	-2.8051484058	0.7876435714
H	-2.9321265886	-0.6439835972	2.2081997662
O	0.2022601616	-0.3823157069	-1.4838054004
P	0.8835775429	-0.0359269491	-0.2053184719
O	1.9698846988	-1.2107884771	0.1201232775
O	1.8578769447	1.2642834875	-0.3080028413
C	2.9349072740	-1.0036803475	1.0827380073
C	3.0286379810	1.1906480518	-1.0353776210
C	4.0390454262	-0.1905588620	0.7948468282
C	2.8265027512	-1.6803633858	2.2913647347
C	4.1367662590	0.5227880036	-0.4976576833
C	3.1022663716	1.8615673962	-2.2495985948
C	5.0462802481	-0.0921641369	1.7601935214
C	3.8398435529	-1.5656506342	3.2362507277
C	5.3355427769	0.5644693289	-1.2167554438
C	4.3046628797	1.8863500362	-2.9472331858
H	2.2118129820	2.3572433006	-2.6202799599
C	4.9543856627	-0.7730716737	2.9687764057
C	5.4250270138	1.2406077032	-2.4280827795
H	4.3653623480	2.4087333616	-3.8966989768
H	5.7457548329	-0.6767264541	3.7050650670
H	5.9010393741	0.5470893437	1.5571885084
H	6.1987592321	0.0369229023	-0.8205299451
H	6.3640424760	1.2533864191	-2.9718904359
C	-2.7687154343	1.6321438173	0.5103103896
C	-3.5579760701	2.3329122786	-0.3917794884
C	-2.2431001738	2.2377313901	1.6476370757
C	-3.8505046102	3.6684659191	-0.1270886755
H	-3.9058162064	1.8365502105	-1.2897726221
C	-2.5444912921	3.5726174009	1.8982321899
C	-3.3522135833	4.2870356063	1.0166330629
H	-4.4646290794	4.2281985922	-0.8251966953
C	-4.7479561215	-0.4910796538	0.8841897127
H	-5.0266864361	0.5565554593	1.0101461281
H	-5.3482795566	-1.0797020054	1.5828888286
C	-3.2778144855	-2.4013133406	-2.1046570557
H	-4.0432141131	-3.1860799396	-2.0440400519
H	-3.4554351832	-1.8492689534	-3.0348917252
C	-1.6354264018	-2.7046522565	0.4006201354
H	-0.8604085083	-1.9527095799	0.2457987189
H	-1.4178689137	-3.1885947093	1.3588986828
C	-1.5783628673	-3.7154017768	-0.7525257970
H	-0.5866888664	-4.1765547755	-0.7842078247
H	-2.3040153747	-4.5218080679	-0.5706252713
C	-1.8822359612	-3.0455078083	-2.0977475079
H	-1.8268977817	-3.7827744668	-2.9053002284
H	-1.1082945869	-2.2949735203	-2.2998032937
C	-4.8977084640	-1.0024554269	-0.5829454231

H	-5.2199396997	-0.2192504359	-1.2734843865
H	-5.6077549315	-1.8325065948	-0.6471336214
H	1.9472752669	-2.2923064804	2.4619208034
H	3.7584811386	-2.0940788867	4.1809096657
H	-1.5761959717	1.6799484703	2.2981526877
H	-2.1328132557	4.0571446140	2.7774187704
H	-3.5801940912	5.3297307838	1.2130461987
H	-1.7070120202	-0.4333365310	-1.4119153187

2i

0 1

C	3.0643449008	1.6574503249	-1.3922395362
N	2.5448557215	0.2689867666	-1.2801333958
N	2.5014870561	0.0048166864	0.1540021036
H	1.4470978684	-0.0800291727	0.5139981957
O	0.1323946738	-0.3283488749	1.0850575275
C	3.1171707767	1.2428138884	0.7934031219
C	2.5322041681	2.3431365540	-0.1016636642
H	3.0710813186	3.2802840968	0.0921255998
H	2.8545760160	1.2479965642	1.8527252178
O	-0.3028224818	-0.2460513610	-1.4513111914
P	-0.8503352518	-0.3710558177	-0.0698972872
O	-1.9771434652	0.8015912993	0.1129063635
O	-1.7518904078	-1.7050568617	0.1614714638
C	-2.8782266083	0.7425995319	1.1537975667
C	-2.9627355651	-1.8307318255	-0.4891266565
C	-3.9476943172	-0.1618314323	1.0991396592
C	-2.7514971203	1.6524767521	2.1964708217
C	-4.0752943440	-1.1198721850	-0.0212811587
C	-3.0633854665	-2.7307748969	-1.5423027536
C	-4.8963110067	-0.1138874508	2.1257323491
C	-3.7067149767	1.6805991541	3.2061899270
C	-5.3073491670	-1.3566511581	-0.6397471604
C	-4.2986469079	-2.9470889823	-2.1426553569
H	-2.1672901241	-3.2484144669	-1.8663613328
C	-4.7837345410	0.7972832466	3.1696184340
C	-5.4239201349	-2.2623987849	-1.6877990031
H	-4.3812915969	-3.6490424307	-2.9663495351
H	-5.5294093701	0.8114532018	3.9578865701
H	-5.7214258163	-0.8204047788	2.1044239514
H	-6.1763929970	-0.8005272396	-0.2992085485
H	-6.3889527272	-2.4247955752	-2.1568664290
C	3.1273013719	-1.2619009995	0.5396330990
C	4.0246604786	-1.9122214944	-0.2970550128
C	2.7837196460	-1.7755981471	1.7863425178
C	4.6133733209	-3.0944721417	0.1442101442
H	4.2273820049	-1.5030095485	-1.2794998508
C	3.3806341356	-2.9578755172	2.2128032200
C	4.2991480409	-3.6145613716	1.3971751786
H	5.3148744645	-3.6139489629	-0.5006719419
C	4.6062299090	1.2678313044	0.4409047542
H	5.1163312824	0.3379824504	0.6990898468
H	5.0874292687	2.0756125870	0.9986525594
C	2.7216777770	2.2237265580	-2.7633884820

C	1.0250203861	2.5558339010	-0.0196268531
H	0.5218211383	1.7252140597	-0.5143127728
H	0.6990485786	2.5279535177	1.0269615532
C	0.5379254658	3.8453754098	-0.6814111510
H	0.7854637386	4.7115195426	-0.0551812705
H	-0.5557786950	3.7913699441	-0.7235106794
C	1.0962090597	4.0736452333	-2.0964520840
H	2.0590775166	4.5990877189	-2.0297194844
C	4.5734747778	1.5589722613	-1.0873965745
H	5.0369847273	0.7680219937	-1.6826960643
H	5.0719191542	2.5036226780	-1.3249757377
H	-1.9055633493	2.3316784571	2.1880858311
H	-3.6094283449	2.3913534202	4.0208064930
H	2.0306773362	-1.2755832044	2.3880268668
H	3.1138582082	-3.3723248261	3.1793926428
H	4.7582329760	-4.5392762022	1.7317099153
H	1.5644345341	0.1859838017	-1.5912156025
C	1.3071406382	2.7974763128	-2.9307320341
H	0.4236135754	4.7560525883	-2.6263238514
H	1.1811583766	3.0371405809	-3.9917497410
H	0.5442860285	2.0423332799	-2.6984051000
H	3.4414745729	3.0208653496	-2.9882542866
H	2.9021137933	1.4284896930	-3.4975679105

2j

0 1

C	3.1165578321	1.7080083287	-0.5508267417
N	2.6784929337	0.3540666897	-0.9788130198
N	2.6057659880	-0.4179234860	0.2565617719
H	1.5436968405	-0.6881649212	0.4617820876
H	1.7115913394	0.3677846750	-1.3402122872
O	0.2084339773	-1.2290359419	0.6744560023
C	3.1176883287	0.5283087706	1.3402228503
C	2.4838405087	1.8485946733	0.8763304291
H	2.9555518997	2.6766117800	1.4209638899
H	2.8098730337	0.1342777953	2.3105585480
O	-0.2022501959	0.0906271520	-1.5107876153
P	-0.7561644821	-0.6677550047	-0.3526103298
O	-1.8401116271	0.3103716438	0.3910085374
O	-1.7131273352	-1.9123207899	-0.7790701343
C	-2.7242334204	-0.2173616126	1.3070205531
C	-2.9362338036	-1.6669521395	-1.3699506738
C	-3.8321831663	-0.9482326905	0.8575635039
C	-2.5378654242	0.0629213491	2.6555435682
C	-4.0128615092	-1.2386266087	-0.5820439606
C	-3.0880291348	-1.9388038375	-2.7237023738
C	-4.7626922635	-1.3745625064	1.8104993560
C	-3.4766188920	-0.3723814204	3.5845882199
H	-1.6585195409	0.6269714238	2.9508637860
C	-5.2611531666	-1.1076909216	-1.1991256885
C	-4.3385260835	-1.7983151792	-3.3149596767
H	-2.2189252709	-2.2624757878	-3.2857319993
C	-4.5936160405	-1.0891832635	3.1607933835
H	-3.3343775316	-0.1532980135	4.6381027212

C	-5.4285547672	-1.3865269491	-2.5506481071
H	-4.4605539799	-2.0092879729	-4.3726094258
H	-5.3264621516	-1.4353839653	3.8823695174
H	-5.6185862906	-1.9556355362	1.4785551444
H	-6.1019762267	-0.7608926377	-0.6047766833
H	-6.4052522915	-1.2713221161	-3.0093454327
C	3.2997794965	-1.7056911766	0.1903421541
C	4.2740590245	-1.9589984598	-0.7656928313
C	2.9364723205	-2.6583357908	1.1367329643
C	4.9208925839	-3.1921570611	-0.7491461838
H	4.4905138667	-1.2078784105	-1.5159896711
C	3.5916275427	-3.8856058847	1.1405648173
H	2.1252731895	-2.4512324918	1.8284384089
C	4.5875159781	-4.1514531844	0.2036888270
H	5.6831281668	-3.4045387021	-1.4918839528
H	3.3105299862	-4.6394375757	1.8684836768
H	5.0924398255	-5.1120620351	0.2063984816
C	4.6117711701	0.7449183512	1.1166379245
H	5.1731216947	-0.1890951631	1.0529308774
H	5.0135618884	1.3169219786	1.9572123974
C	4.6157342971	1.5677961929	-0.1997229677
H	5.1496495474	1.0696569884	-1.0133742303
H	5.0628299392	2.5557096672	-0.0538159638
C	0.9537052043	1.8926708382	1.0145039659
H	0.5094811615	1.7730909966	0.0270398749
H	0.6134428803	1.0163553425	1.5782480536
C	0.3678274059	3.1436779450	1.6880111002
H	0.3404396443	2.9950220840	2.7752771154
C	2.8955036410	2.6842934509	-1.7066081337
H	3.3587603145	2.2033291691	-2.5772730860
H	3.4911984192	3.5839033104	-1.5040883575
C	1.4555850835	3.1111231314	-2.0358957469
H	0.7549924722	2.2804205488	-1.8927962037
H	1.3979954946	3.3566006738	-3.1024251307
C	-0.4428333160	4.6069420042	-1.0692557146
C	1.0610270876	4.3469728756	-1.2173042006
H	1.5314138708	4.2781265423	-0.2333446487
H	1.5212825008	5.2251045460	-1.6901318594
C	-1.0541864009	3.4636796308	1.1975279439
H	-1.7375712119	2.6938197016	1.5768208986
H	-1.3706204297	4.4119382268	1.6523448278
C	-1.2614429543	3.5438448174	-0.3235782559
H	-1.1170130002	2.5569628386	-0.7789994357
H	-2.3223480266	3.7719517459	-0.4828817352
H	-0.8675286147	4.7230611236	-2.0753573291
H	-0.5802180005	5.5754506102	-0.5681947317
H	1.0174191705	4.0108326242	1.5346363407

2k

0 1

C	2.7459664221	1.3381859700	-0.9981870595
N	2.3578505436	-0.0959045689	-1.0925490558
N	2.1499366013	-0.5988313110	0.2484938835
H	1.0426080313	-0.6924163813	0.4526085570

O	-0.2883447366	-0.7990810661	0.9067931750
C	2.5523352807	0.4803782662	1.2520735706
C	2.3071518636	1.7654687426	0.4397773138
H	2.9799953004	2.5408452473	0.8235313135
H	1.8072183879	0.3780879074	2.0460331043
O	-0.5294145117	-0.1083002816	-1.5604386950
P	-1.1873111091	-0.4621311247	-0.2721917378
O	-2.1909904900	0.7630312835	0.1297678995
O	-2.2378356440	-1.6994832129	-0.3835069958
C	-3.1512753104	0.5822750582	1.1023991515
C	-3.4167266508	-1.5354247863	-1.0824756992
C	-4.3052640252	-0.1582476721	0.8129182206
C	-2.9866966653	1.2199598780	2.3259972404
C	-4.4717144430	-0.8225062776	-0.4986886097
C	-3.5544011033	-2.1606353230	-2.3152354693
C	-5.2984318430	-0.2290374029	1.7952103566
C	-3.9876083842	1.1345068713	3.2870829567
H	-2.0751258777	1.7832682062	2.4960918292
C	-5.6858563064	-0.7708828871	-1.1909931461
C	-4.7705965510	-2.0928175676	-2.9855214110
H	-2.7022700813	-2.6941964378	-2.7215960694
C	-5.1476691587	0.4101941939	3.0204469454
H	-3.8610700871	1.6324980542	4.2432179295
C	-5.8400617469	-1.4006970174	-2.4206244749
H	-4.8817997036	-2.5793597772	-3.9493282520
H	-5.9296576061	0.3355250229	3.7691192988
H	-6.1910321180	-0.8137305054	1.5907982089
H	-6.5082490370	-0.2075843799	-0.7588173918
H	-6.7894707352	-1.3414175448	-2.9428471686
C	2.6634970548	-1.9491192234	0.4678106236
C	2.2357285418	-2.6014931189	1.6230039211
C	3.4875369732	-2.5788439050	-0.4545755565
C	2.6886590279	-3.8903884592	1.8788742150
H	1.5345567287	-2.1121034398	2.2928392595
C	3.9308312810	-3.8728036554	-0.1860134059
H	3.7455111230	-2.0650606538	-1.3723008773
C	3.5435169618	-4.5256849338	0.9796133084
H	2.3588911863	-4.4042140637	2.7758564197
H	4.5754238472	-4.3727930141	-0.9018021222
H	3.8905775520	-5.5342045319	1.1797346208
C	4.2648728501	1.4683218066	-1.2147598403
H	4.4573687350	1.2205337180	-2.2662917214
H	4.5411610429	2.5234459464	-1.0831910742
C	0.8522078176	2.2653191372	0.5798609469
H	0.2127397558	1.8243154020	-0.1899623210
H	0.4550440727	1.8926068261	1.5309035201
C	0.6765244438	3.7879374026	0.5806759937
H	1.3632629661	4.2245199808	1.3209543025
H	-0.3372451170	3.9964675473	0.9429531693
C	0.8581430796	4.5237000068	-0.7506281359
H	0.5734208689	5.5684538755	-0.5839099551
H	0.1395837176	4.1306337085	-1.4825018994
C	2.2785448843	4.4781756723	-1.3434869878
H	2.5429054697	5.4660698203	-1.7367222224
H	3.0070293531	4.2770073490	-0.5460221153
C	2.0256367412	2.0334502396	-2.1685382256

H	0.9435360764	1.9754839548	-2.0176507527
H	2.2270376455	1.4199253877	-3.0568273934
C	2.4468386490	3.4692390038	-2.4828143275
H	3.4845572433	3.4882773767	-2.8355841095
H	1.8351195057	3.8051460675	-3.3293936268
C	5.1268369030	0.9565948522	1.1446053245
H	5.1125399338	2.0480278161	1.2645375682
H	6.0399553973	0.6117494107	1.6417599946
C	3.9417424412	0.3285769385	1.8816055675
H	3.8724017800	0.7777761015	2.8790518079
H	4.1388747182	-0.7357612838	2.0484637362
C	5.1887293699	0.6177090728	-0.3437815480
H	4.9730128285	-0.4469542935	-0.4891739486
H	6.2119478166	0.7737863581	-0.7033687939
H	1.4236722275	-0.1556103794	-1.5223200579

TS1a

0 1

C	2.9218828488	-1.0549896897	1.4984755302
N	2.2511545186	0.0649276178	1.1021536977
N	2.3841116799	0.3872123642	-0.2368533410
H	1.4361294172	0.4172402418	-0.6749444222
O	-0.1522679789	-0.0914824193	-1.0572040304
C	2.5646115152	-1.9719108508	-0.8342570270
C	2.2408632184	-2.5153677439	0.3947327080
H	2.9930724024	-3.1855173718	0.8123176645
H	1.7905149502	-1.6355493153	-1.5201607852
O	-0.4297330232	0.5975216457	1.4195367235
P	-1.0478883667	0.2589534957	0.0970709145
O	-2.1089644483	-0.9623256864	0.3815338108
O	-2.0686651443	1.4232737352	-0.4194105956
C	-3.0782216028	-1.2560018880	-0.5509423626
C	-3.2303226082	1.6746624685	0.2774088122
C	-4.2059037596	-0.4309898324	-0.6641431807
C	-2.9556154405	-2.4199358309	-1.3006193343
C	-4.3212332765	0.8056381260	0.1400758340
C	-3.3200122416	2.8378358821	1.0322479658
C	-5.2176753732	-0.8239151180	-1.5459459479
C	-3.9735272214	-2.7890528044	-2.1726392425
H	-2.0603246555	-3.0204279522	-1.1784254505
C	-5.5180718837	1.1532815454	0.7746957421
C	-4.5197787756	3.1608416387	1.6561151010
H	-2.4429574577	3.4709769636	1.1090196491
C	-5.1097142102	-1.9916284111	-2.2928792622
H	-3.8785728729	-3.6978437516	-2.7587109322
C	-5.6228972680	2.3197594540	1.5237467443
H	-4.5922868198	4.0688838685	2.2464208622
H	-5.9050359116	-2.2724281953	-2.9757582986
H	-6.0898006280	-0.1850698569	-1.6546547500
H	-6.3671209559	0.4805918314	0.6895186431
H	-6.5599429159	2.5661025521	2.0126758988
C	3.2668432635	1.4104533416	-0.6384675849
C	3.3818106942	1.6447663221	-2.0144842042
C	4.0206131156	2.1683997419	0.2601852579

C	4.2563607971	2.6172625845	-2.4809948749
H	2.7693991819	1.0664739803	-2.7020584518
C	4.9018731257	3.1324468711	-0.2224853158
H	3.8828221469	2.0176777931	1.3260143841
C	5.0292000544	3.3603603211	-1.5893104506
H	4.3340128743	2.7956075601	-3.5489863144
H	5.4808414415	3.7209285029	0.4827699963
H	5.7134489950	4.1175103894	-1.9571789548
C	4.0115589888	-1.8901200483	-1.2115074159
H	4.1879487720	-0.9594185085	-1.7616573108
H	4.2686509621	-2.7101158794	-1.8969113901
C	2.3520143059	-1.6545027058	2.7722501427
H	2.3527280404	-0.9073730825	3.5730896015
H	3.0047035940	-2.4740549073	3.0884699571
C	0.8714563992	-2.6846125469	1.0001605142
H	0.1280818087	-2.1435833381	0.4114513432
H	0.5903132344	-3.7422563443	0.9796812421
C	0.9219895488	-2.1587242922	2.4669787964
H	0.6489755370	-2.9375485033	3.1829660001
H	0.2002159780	-1.3457210821	2.5802186883
C	4.4283568595	-1.0853248452	1.2064987036
H	4.7494570809	-0.0579020541	1.0232636475
H	4.9347749885	-1.4158152555	2.1201766706
C	4.9097282458	-1.9700274016	0.0333828370
H	5.9268536369	-1.6688664154	-0.2321446048
H	4.9830283438	-3.0102294033	0.3678208026
H	1.2667592742	0.1934444930	1.4179549490

TS1b

0 1

C	2.7904806596	-1.1923372593	1.3408814055
N	2.1895677809	-0.0349096124	0.9549463033
N	2.4074878843	0.3777465548	-0.3460313426
H	1.4756497489	0.4756398161	-0.8113305972
O	-0.1674508602	0.1766937851	-1.2279013165
C	2.7204900807	-1.9361041489	-1.0950159793
C	2.2112235574	-2.5480409418	0.0401474618
H	2.8602145538	-3.3014365792	0.4957278431
H	2.0409538886	-1.5352864497	-1.8425149094
O	-0.4003937525	0.8190476253	1.2665662885
P	-1.0414034486	0.4766191448	-0.0440754651
O	-2.0393289704	-0.7971874137	0.2615623766
O	-2.1287864960	1.5976102132	-0.5137804211
C	-3.0323257117	-1.1238154196	-0.6333317610
C	-3.2740263767	1.7946857257	0.2264164085
C	-4.1956624524	-0.3437369137	-0.6975972791
C	-2.8963068612	-2.2808285700	-1.3917222509
C	-4.3316259802	0.8815297921	0.1207373800
C	-3.3843862829	2.9475311800	0.9942953334
C	-5.2248993786	-0.7724661117	-1.5417699746
C	-3.9323849081	-2.6863618172	-2.2253292522
H	-1.9744911036	-2.8471685445	-1.3079357165
C	-5.5179318903	1.1738712652	0.8015926278
C	-4.5729061136	3.2151801884	1.6640005607

H	-2.5323688830	3.6165250711	1.0444819446
C	-5.1016697938	-1.9323714943	-2.2983817847
H	-3.8257779137	-3.5893680143	-2.8183222120
C	-5.6439645746	2.3292408825	1.5643178366
H	-4.6616900663	4.1150801199	2.2644524365
H	-5.9115734467	-2.2410803512	-2.9513965747
H	-6.1247588527	-0.1676901167	-1.6128059507
H	-6.3407237578	0.4667334399	0.7413567369
H	-6.5720134928	2.5322371372	2.0890081489
C	3.2656773929	1.4828495159	-0.5717774965
C	3.9628038781	2.1195181041	0.4558898981
C	3.4299732158	1.9033366303	-1.8950755302
C	4.8369289941	3.1593151497	0.1497536005
H	3.7968485593	1.8099110326	1.4822307746
C	4.2965333756	2.9493580136	-2.1844685438
H	2.8703204026	1.4060852558	-2.6830051367
C	5.0116435783	3.5781508606	-1.1658155423
H	5.3744888943	3.6537841440	0.9531590120
H	4.4142662917	3.2745552893	-3.2134838507
H	5.6898285320	4.3930575240	-1.3957647059
C	4.1978581966	-1.9361137539	-1.3216048880
H	4.4523027386	-1.4950582181	-2.2881585229
H	4.5440706773	-2.9774516933	-1.3327943731
C	2.2407310287	-1.7305471053	2.6553550445
H	2.7778274269	-2.6559807253	2.8934588891
H	2.5018832418	-1.0046910653	3.4361600162
C	0.7266183373	-2.7696857325	0.2179727320
H	0.1913012939	-1.8909687879	-0.1542696051
H	0.4258820318	-3.6071811263	-0.4255663005
C	0.3298665145	-3.0864026507	1.6610028181
H	-0.7544676275	-3.2285503342	1.7022000966
H	0.7918037718	-4.0392447549	1.9594273027
C	0.7292797986	-1.9973209001	2.6566594560
H	0.4477217595	-2.3090013019	3.6679216295
H	0.1649814750	-1.0817186036	2.4460748725
C	4.3063052748	-1.3258148578	1.1916642117
H	4.7422275379	-0.5711787004	1.8611280999
H	4.5771853811	-2.3023012223	1.6080429697
C	4.9365170771	-1.1673322038	-0.1960227058
H	4.9895473135	-0.1139694969	-0.4667634559
H	5.9692733087	-1.5251783119	-0.1409044385
H	1.2174905413	0.1687688436	1.2643063671

TS1c

0 1

C	2.8442394910	-1.0304046026	0.9799071738
N	2.1344212460	0.1233173851	0.9234374285
N	2.2516549026	0.8758172529	-0.2230644871
H	1.2967901142	1.0367858257	-0.6172008315
H	1.1448530613	0.0832030932	1.2592587010
O	-0.2934439765	0.6411133769	-1.0849924868
C	2.5182390757	-1.1006550626	-1.5968204592
C	2.1634887421	-2.0460674141	-0.6603191823
H	2.9317211818	-2.7873688725	-0.4447624470

H	1.7542037551	-0.5896239599	-2.1787966106
O	-0.5496416417	0.0646677327	1.4257305387
P	-1.1789172194	0.3624594043	0.0944665078
O	-2.1965776843	-0.8845693492	-0.2209499743
O	-2.2453737509	1.5950604272	0.2019791292
C	-3.2036573640	-0.7380974365	-1.1482628145
C	-3.3778426465	1.4462121069	0.9720066881
C	-4.3524950815	-0.0043938648	-0.8199122845
C	-3.0951816050	-1.4027366060	-2.3642746670
C	-4.4559779495	0.7010455188	0.4763262480
C	-3.4553509746	2.1129376788	2.1886854111
C	-5.3976345557	0.0258043366	-1.7485627671
C	-4.1466260661	-1.3568237981	-3.2727003084
H	-2.1839266466	-1.9532649273	-2.5720615257
C	-5.6288613208	0.6614789165	1.2370052204
C	-4.6309621820	2.0567537667	2.9286659037
H	-2.5880628092	2.6687673482	2.5275738400
C	-5.3031264754	-0.6442804767	-2.9629610737
H	-4.0615663051	-1.8758694519	-4.2223063637
C	-5.7220688472	1.3336721625	2.4502835490
H	-4.6942700772	2.5768071846	3.8793718401
H	-6.1249013522	-0.6009062283	-3.6704318272
H	-6.2862523314	0.6046343007	-1.5121880993
H	-6.4673143025	0.0748942650	0.8715127296
H	-6.6400121742	1.2838518525	3.0270777793
C	3.1306239338	1.9770004677	-0.2645949811
C	3.8844221482	2.3952418253	0.8342165913
C	3.2382415681	2.6599233969	-1.4824754530
C	4.7593287761	3.4704209102	0.7006340506
H	3.7507602731	1.8979128215	1.7895551489
C	4.1054035625	3.7381942932	-1.5979229398
H	2.6283647298	2.3396471053	-2.3235854448
C	4.8788802243	4.1441199687	-0.5108016761
H	5.3385161219	3.7918987886	1.5608690544
H	4.1775138651	4.2645494542	-2.5445401933
H	5.5578487420	4.9848075317	-0.6056343380
C	3.9680846548	-0.8560435860	-1.8805774910
H	4.1366040949	0.2143353896	-2.0425031840
H	4.2546548246	-1.3572336221	-2.8158419178
C	2.4216122942	-1.8755629798	2.1722377923
H	3.0627567052	-1.5451446943	3.0000156602
H	1.3963572184	-1.5975979384	2.4460480220
C	2.5110543937	-3.4078236581	2.0426844285
H	3.3463955361	-3.7029200815	1.3928454547
H	2.7608813040	-3.7999718074	3.0338331147
C	4.3479876296	-0.9698692931	0.6661935509
H	4.6728871210	0.0546343856	0.8678497376
H	4.8539264060	-1.6065553249	1.4003896317
C	4.8432902791	-1.3777999467	-0.7367261966
H	5.8661215399	-1.0099574223	-0.8581184502
H	4.9091116319	-2.4698642432	-0.7927623309
C	1.1977030189	-4.0851744257	1.6095957699
H	0.3974342171	-3.6818881524	2.2436170837
H	1.2709394828	-5.1520770033	1.8507337154
C	0.7546544384	-2.5153815510	-0.3820743725
H	0.2613774714	-1.8682799899	0.3527301329

H	0.1528245089	-2.4371141514	-1.2936851414
C	0.7679096330	-3.9545336427	0.1434166412
H	1.4267519092	-4.5635015583	-0.4913279985
H	-0.2347704883	-4.3841321512	0.0501720498

TS1d

0 1

C	-2.7470331052	-0.9363858929	-0.9889669047
N	-2.1213189892	0.2633377694	-0.8432982905
N	-2.2974722145	0.9232431417	0.3566630451
H	-1.3482678175	1.0966849708	0.7593244117
O	0.3110430460	0.8084783574	1.1048839516
C	-2.5990303381	-1.1729732969	1.5655462430
C	-2.1026333814	-1.9840534972	0.5589862867
H	-2.7640372747	-2.8018198311	0.2668833293
H	-1.9106282024	-0.6507565463	2.2247172492
O	0.5521126214	0.3657842323	-1.4365655451
P	1.1863289162	0.5824510292	-0.0929730597
O	2.1844208071	-0.7014883170	0.1467719004
O	2.2754146473	1.7990974559	-0.1262639667
C	3.1812719416	-0.6373063065	1.0929954994
C	3.4183978553	1.6762196695	-0.8841408571
C	4.3431201784	0.1031505256	0.8324102519
C	3.0513949298	-1.3914503273	2.2537747604
C	4.4774812863	0.8877474348	-0.4147615178
C	3.5275653183	2.4128035875	-2.0575389093
C	5.3750525780	0.0548416788	1.7750582927
C	4.0904884527	-1.4232444636	3.1768938549
H	2.1347746189	-1.9520844010	2.4064453470
C	5.6633382882	0.8764807685	-1.1561792683
C	4.7155925156	2.3836994600	-2.7789491514
H	2.6745476854	3.0011325429	-2.3772572478
C	5.2567456473	-0.6999316060	2.9365227045
H	3.9885287601	-2.0115273267	4.0834941895
C	5.7880513852	1.6179643262	-2.3254235264
H	4.8031359795	2.9584233254	-3.6956516663
C	-3.1520098942	2.0529435593	0.3906600444
C	-3.2900110088	2.7148099531	1.6144912904
C	-3.8722977452	2.4865644113	-0.7233860133
C	-4.1520156364	3.7985678629	1.7201401602
H	-2.7159573528	2.3714602271	2.4710968659
C	-4.7420920279	3.5669357044	-0.5999431221
H	-3.7289281295	1.9875241119	-1.6759019070
C	-4.8895303992	4.2262989809	0.6166896606
H	-4.2495830426	4.3109632577	2.6722081409
H	-5.2983871747	3.9017367009	-1.4702214874
H	-5.5647265143	5.0709136711	0.7038089800
C	-4.0732154176	-1.1338752547	1.8163976068
H	-4.3122550271	-0.5156771503	2.6852151604
H	-4.4087283063	-2.1547892288	2.0398043267
C	-4.2549302347	-1.0279458571	-0.7664628041
H	-4.7104359469	-0.4084691736	-1.5516452039
H	-4.5448216611	-2.0614081366	-0.9854744882
C	-4.8433046910	-0.6027756998	0.5833723404

H	-4.8914411362	0.4837388365	0.6418780980
H	-5.8769170963	-0.9598742092	0.6279750914
C	-0.6207767895	-2.2222966312	0.3872635479
H	-0.2653219642	-1.7859445076	-0.5532126821
H	-0.0795056524	-1.6785171159	1.1667677394
C	-0.2407873668	-3.7026728725	0.4092459616
H	-0.3654248499	-4.1089180245	1.4209216734
H	0.8276850337	-3.7695850391	0.1718463027
C	-1.0297816093	-4.5767914272	-0.5735939211
H	-2.0410999235	-4.7689254110	-0.1892000341
H	-0.5448334923	-5.5587214343	-0.6039642137
C	-1.1385260401	-4.0370389029	-2.0118654754
H	-0.2250729924	-3.4880374144	-2.2764533611
H	-1.1689622354	-4.9028595671	-2.6816344415
C	-2.2438159971	-1.6459111928	-2.2451016099
H	-1.2020282037	-1.3492230220	-2.4164397833
H	-2.8205268851	-1.1878622831	-3.0597271749
C	-2.3763663232	-3.1771165797	-2.3594345511
H	-3.2446157427	-3.5429408593	-1.7947900454
H	-2.6171993866	-3.3784972440	-3.4078654891
H	6.2733752399	0.6381259241	1.5924862608
H	6.4869054722	0.2568847355	-0.8119188830
H	6.0686982939	-0.7163243428	3.6563605957
H	6.7157641123	1.5890719183	-2.8878349392
H	-1.1323863907	0.3071752629	-1.1784426230

TS1e

0 1

C	-2.7607233313	-0.8319198183	-0.8565334129
N	-2.0607006026	0.3356149423	-0.8778689938
N	-2.2041934084	1.1663089359	0.2098621700
H	-1.2442962547	1.4070102420	0.5584562007
O	0.4040857831	1.2431202868	0.9132221851
C	-2.6436100509	-0.5704064513	1.7265540935
C	-2.1221339291	-1.5886543323	0.9473548062
H	-2.7832770894	-2.4341809514	0.7722347334
H	-1.9874426508	0.0179183736	2.3637881378
O	0.6158250261	0.1828849934	-1.4409815338
P	1.2645018993	0.6906997881	-0.1853414410
O	2.2004735642	-0.5419796693	0.3663811070
O	2.4101602529	1.8097649289	-0.5035002518
C	3.2005604556	-0.2977096285	1.2794525562
C	3.5452066373	1.4527708128	-1.1967439948
C	4.3965989875	0.3009963124	0.8589668407
C	3.0347791580	-0.7394154061	2.5871773492
C	4.5660030046	0.7526362461	-0.5397579147
C	3.6878265823	1.8772758322	-2.5123246552
C	5.4264587107	0.4336141306	1.7958328585
C	4.0726221490	-0.5959802654	3.5010868350
H	2.0911581134	-1.1997034457	2.8619623620
C	5.7485695512	0.5062645674	-1.2447920606
C	4.8717920698	1.6184739197	-3.1934231546
C	5.2728660735	-0.0100740018	3.1043728372
H	3.9431916645	-0.9403466358	4.5223830488

C	5.9068468620	0.9356806440	-2.5574043591
H	6.5419326369	-0.0496380118	-0.7526626774
C	-3.0996698354	2.2611001019	0.1478348885
C	-3.8116818894	2.5998088106	-1.0037125774
C	-3.2560310913	3.0198083332	1.3131755454
C	-4.6967039066	3.6747819465	-0.9744392464
H	-3.6391070962	2.0402358578	-1.9173539318
C	-4.1327117686	4.0969637028	1.3255329156
H	-2.6786562110	2.7593234530	2.1966390252
C	-4.8652568625	4.4249082875	0.1852006262
H	-4.2441926431	4.6831848937	2.2322639900
C	-4.1254938276	-0.4068341606	1.8605155870
H	-4.3772311542	0.6608468040	1.8726072414
H	-4.4521625614	-0.8091777429	2.8294490900
C	-4.2828084049	-0.7561384444	-0.6553488271
H	-4.5918020626	0.2648297989	-0.8957449235
H	-4.7430438605	-1.4073029199	-1.4065547490
C	-4.8576399156	-1.1194679902	0.7234360753
H	-5.9181620688	-0.8519609182	0.7368585934
H	-4.8159313181	-2.2045814579	0.8700677513
C	-0.6398091292	-1.8713984631	0.9129801771
H	-0.2284912810	-1.6333634585	-0.0742350862
H	-0.1336925465	-1.1772175469	1.5916102278
C	-0.2616993377	-3.3132768367	1.2771430783
H	-0.5491281886	-3.5156714149	2.3167309500
H	0.8321692318	-3.3854897174	1.2306830376
C	-2.2726133570	-1.7881438923	-1.9315180567
H	-1.1778289657	-1.7519335559	-1.9475900402
H	-2.5910017715	-1.3528585242	-2.8878061717
C	-2.7913739390	-3.2348052676	-1.8316753000
H	-3.2512218241	-3.4131052800	-0.8535341254
H	-3.5967834210	-3.3887615659	-2.5579221752
C	-0.8729960298	-4.3920038913	0.3718735531
H	-1.9637670592	-4.3953591179	0.4951481542
H	-0.5461625203	-5.3719638691	0.7359659164
C	-0.5077319491	-4.2430766344	-1.1205385272
H	0.0511022589	-3.3133528706	-1.2773632946
H	0.1847370708	-5.0405347115	-1.4097257071
C	-1.7051570845	-4.2915955372	-2.0802187244
H	-2.1751466359	-5.2813830408	-2.0200675230
H	-1.3375168407	-4.1852918596	-3.1084985378
H	-1.0645483275	0.2645236829	-1.1904249783
H	-5.2443174588	3.9351820094	-1.8749876667
H	-5.5512612215	5.2651981237	0.1987903230
H	2.8635615464	2.4102507547	-2.9733109211
H	6.3520794855	0.9113606288	1.4867391591
H	4.9852949768	1.9494160847	-4.2209948070
H	6.0840698355	0.1098743274	3.8151956052
H	6.8311730963	0.7277537498	-3.0867222850

TS1f

0 1

C	0	2.7682296734	-0.7044048915	0.6875695893
N	0	2.0248242743	0.4266346007	0.8498891377

N	0	2.1288849816	1.3789015072	-0.1399250830
H	0	1.1562190086	1.6301798822	-0.4456765618
O	0	-0.4972811603	1.5111916904	-0.7751532928
C	0	2.6342694783	-0.1629571144	-1.8459654339
C	0	2.1279038048	-1.2641928337	-1.1773384474
H	0	2.7966978143	-2.1185576783	-1.0902380446
H	0	1.9687335532	0.4786409576	-2.4185259267
O	0	-0.6398982109	0.1362649375	1.4162760227
P	0	-1.3222051093	0.7847216929	0.2467138003
O	0	-2.2206245197	-0.3959093660	-0.4609819332
O	0	-2.5039590469	1.8070152992	0.7208241493
C	0	-3.2343380322	-0.0641302494	-1.3300786343
C	0	-3.6199116960	1.3178528249	1.3623394588
C	0	-4.4476834712	0.4274162717	-0.8282569443
C	0	-3.0615451742	-0.3131940082	-2.6868102088
C	0	-4.6210857350	0.6773580463	0.6196819915
C	0	-3.7663019857	1.5536152451	2.7240551582
C	0	-5.4885191585	0.6502142001	-1.7353200878
C	0	-4.1104696202	-0.0828071604	-3.5698386899
H	0	-2.1028678063	-0.6938460926	-3.0245071824
C	0	-5.7882758217	0.2948116273	1.2887696782
C	0	-4.9346447271	1.1618951995	3.3677635128
C	0	-5.3284523650	0.3974249194	-3.0930003624
H	0	-3.9759302441	-0.2766772627	-4.6294096376
C	0	-5.9503697067	0.5352536599	2.6482977761
H	0	-6.5657566159	-0.2170325540	0.7282112982
C	0	2.9837172984	2.4940795924	0.0419463368
C	0	3.1111564300	3.3769956634	-1.0358897247
C	0	3.6812892331	2.7362736663	1.2260910303
C	0	3.9449123327	4.4827870855	-0.9302329524
H	0	2.5442363348	3.1897023862	-1.9442714464
C	0	4.5237806155	3.8416543017	1.3147150866
H	0	3.5317663770	2.0750975352	2.0735853005
C	0	4.6633286545	4.7164062419	0.2417491756
H	0	5.0608128445	4.0259390218	2.2400716469
C	0	4.1120892075	0.0405432659	-1.9648656327
H	0	4.3423944144	1.1087557672	-1.8625134122
H	0	4.4406085065	-0.2489735094	-2.9726527020
C	0	4.2826703660	-0.5329563662	0.4938471859
H	0	4.5394264408	0.4858159712	0.7950840938
H	0	4.7834416751	-1.2021317680	1.2008577861
C	0	4.8607133143	-0.7714276260	-0.9087347608
H	0	5.9178480674	-0.4908049393	-0.9006928045
H	0	4.8291058265	-1.8388417218	-1.1541016068
C	0	0.6443523972	-1.5518449527	-1.1826487988
H	0	0.2346751120	-1.5068165072	-0.1673519002
H	0	0.1365852751	-0.7422762628	-1.7168129941
C	0	0.2689573879	-2.8992564800	-1.8152377098
H	0	-0.8256481558	-2.9783147430	-1.8094951721
C	0	2.3135710274	-1.8064763789	1.6288729096
H	0	1.2256787821	-1.8944073748	1.5474368626
H	0	2.4922189240	-1.4381067873	2.6489030095
C	0	2.9970221966	-3.1747442331	1.4915807391
H	0	3.9846642671	-3.1248325568	1.9629150227
H	0	3.1838295331	-3.4230896534	0.4413804365
C	0	1.3009998997	-5.1452895253	1.2134209177

C	0	2.1747963246	-4.3009521780	2.1549822086
H	0	2.8539791628	-4.9817236875	2.6795539798
H	0	1.5360862794	-3.8566991424	2.9298725211
H	0	1.0342693874	0.2839232204	1.1569955627
H	0	4.0338154496	5.1656702100	-1.7692814560
H	0	5.3159646792	5.5794065642	0.3202122452
H	0	-2.9577044568	2.0485803232	3.2505182031
H	0	-6.4283976544	1.0466744753	-1.3611270370
H	0	-5.0510049561	1.3454275001	4.4312891333
H	0	-6.1486632920	0.5844564322	-3.7785640222
H	0	-6.8623106589	0.2240900829	3.1476641069
C	0	0.8711199756	-4.1138286940	-1.1040766021
H	0	0.6897798061	-5.0128501232	-1.7059003823
H	0	1.9627493515	-4.0130929727	-1.0753224509
C	0	0.3280188559	-4.3588056239	0.3140983263
H	0	0.0587409345	-3.4120804270	0.7956007111
H	0	-0.6159910971	-4.9074323182	0.2243491479
H	0	0.7415895882	-5.8504148493	1.8379736937
H	0	1.9526649616	-5.7601436451	0.5770796127
H	0	0.5832573919	-2.9053016105	-2.8666165268

TS1g

0 1

C	2.8984714356	-1.0609639766	1.5626845239
N	2.2924388898	0.0092566822	1.0020844962
N	2.5379340021	0.2244271405	-0.3367946613
H	1.6157413639	0.3211979540	-0.8243783672
H	1.3002417914	0.2267163730	1.2684451415
O	-0.0469771938	0.0749270830	-1.1313105052
C	3.1134015269	-2.0993146581	-0.5982107629
C	2.4059661529	-2.6144097763	0.4670489356
H	3.0247530230	-3.1986049280	1.1525895370
H	2.7224952832	-1.9292961722	-1.5956809605
O	-0.3150412278	0.6674122362	1.3719351980
P	-0.9383310265	0.3569916754	0.0418448767
O	-1.9522218835	-0.9088056264	0.3068092734
O	-2.0050879340	1.5020859864	-0.4162924825
C	-2.9254353151	-1.2090931089	-0.6196303507
C	-3.1655853720	1.6889986228	0.3029618769
C	-4.0816691895	-0.4198740094	-0.6919997225
C	-2.7750760298	-2.3455897792	-1.4053620240
C	-4.2272059009	0.7866555473	0.1520990106
C	-3.2850706265	2.8241111934	1.0954366835
C	-5.0926881420	-0.8202652151	-1.5713433552
C	-3.7929444637	-2.7228039045	-2.2739808140
H	-1.8584286180	-2.9186783275	-1.3138964151
C	-5.4262712315	1.0720024322	0.8131633935
C	-4.4863857611	3.0849953470	1.7448208430
H	-2.4298611616	3.4855169356	1.1801520163
C	-4.9566755495	-1.9608397337	-2.3546646815
H	-3.6763249426	-3.6099104390	-2.8886258762
C	-5.5611962830	2.2099482612	1.6002235224
H	-4.5822001647	3.9712217205	2.3642303437
H	-5.7519945243	-2.2478167288	-3.0349707848

H	-5.9871887476	-0.2083666983	-1.6487175799
H	-6.2524885336	0.3727993799	0.7176034981
H	-6.4993855320	2.4076641619	2.1086413188
C	3.5373693076	1.1423281775	-0.7309680489
C	4.2996980468	1.8724165800	0.1816043663
C	3.7694354313	1.2790589794	-2.1044899600
C	5.3031527720	2.7189984658	-0.2839040356
H	4.0753553804	1.7999255811	1.2406053695
C	4.7661998402	2.1343442386	-2.5546466591
H	3.1576192409	0.7160860684	-2.8044401661
C	5.5449456825	2.8525736569	-1.6473446450
H	5.8884570332	3.2896759168	0.4305567675
H	4.9361413256	2.2397455780	-3.6215512279
H	6.3247084184	3.5178391624	-2.0026125949
C	4.5297704485	-1.7365991846	-0.2012015158
H	4.9208981517	-0.9456534950	-0.8444218674
H	5.2126137559	-2.5902559809	-0.2855871147
C	2.1401747027	-1.5714122333	2.7729944406
H	2.7584991104	-2.3038895909	3.2991669959
H	1.9289724434	-0.7546184293	3.4700599082
C	0.9428970770	-2.6910194678	0.7796577646
H	0.3816314017	-2.0453824093	0.0989003433
H	0.5505207407	-3.7065710497	0.6710425585
C	0.8041454627	-2.2094856212	2.2634180243
H	0.5456936808	-3.0446643982	2.9195254446
H	-0.0040112986	-1.4777899859	2.3273782849
C	4.3967717391	-1.2597734811	1.2804192899
H	4.9580548455	-0.3393149248	1.4665663679
H	4.7728271452	-2.0183008033	1.9727847636

TS1h

0 1

C	2.7946641305	-1.1634885701	1.4090746393
N	2.2280922977	-0.0358849022	0.9297124358
N	2.5227343234	0.3103083753	-0.3679477390
H	1.6228728058	0.4803915287	-0.8760025748
O	-0.0466937250	0.3263404644	-1.2828695341
C	3.1296221748	-1.9575074386	-0.8681714803
C	2.3184443372	-2.5757149263	0.0571616157
H	2.8377860085	-3.2512786670	0.7430259817
H	2.8198392133	-1.6926708032	-1.8743573552
O	-0.2875453229	0.9403033242	1.2168774191
P	-0.9246558656	0.5874941792	-0.0953310081
O	-1.8768938245	-0.7204943390	0.2144783315
O	-2.0512320090	1.6756235182	-0.5440131413
C	-2.8786498726	-1.0722302736	-0.6613385939
C	-3.1888504500	1.8362451449	0.2172803158
C	-4.0646792794	-0.3251355383	-0.6993361176
C	-2.7262114543	-2.2239803983	-1.4247011495
C	-4.2205726695	0.8926490763	0.1266238198
C	-3.3182678868	2.9819978908	0.9926039017
C	-5.0983242858	-0.7807523272	-1.5238801418
C	-3.7675313584	-2.6567211070	-2.2379729097
C	-5.4020682039	1.1471724122	0.8307772184

C	-4.5014359680	3.2117801362	1.6853805517
H	-2.4853005328	3.6753414380	1.0303285522
C	-4.9583554006	-1.9352554371	-2.2857627411
C	-5.5476957452	2.2950638677	1.6011648548
H	-4.6053903469	4.1061005154	2.2916792655
H	-5.7724369657	-2.2650416199	-2.9230677339
H	-6.0159071877	-0.2011620338	-1.5751785460
H	-6.2048025669	0.4164877830	0.7824502978
H	-6.4713745761	2.4683366325	2.1438977835
C	3.5543902649	1.2404514755	-0.6310288387
C	4.3009002686	1.8498087045	0.3777520124
C	3.8344496722	1.5166303259	-1.9740686010
C	5.3392861415	2.7135210335	0.0369191789
H	4.0383935434	1.6734430469	1.4155334842
C	4.8648689679	2.3885979888	-2.2988818625
C	5.6297272803	2.9845912297	-1.2962136891
H	5.9130394136	3.1896568329	0.8260656467
C	4.5140388611	-1.6602870464	-0.3492130099
H	4.9454227918	-0.8000248913	-0.8650069763
H	5.1971085142	-2.5041514762	-0.5022529383
C	2.1305112840	-1.6912184604	2.6660351479
H	2.6575444408	-2.6012477491	2.9745157561
H	2.2675149948	-0.9573184182	3.4702624523
C	0.8297883292	-2.7565340168	0.0083904686
H	0.3681685365	-1.8568999441	-0.4154688197
H	0.5648138244	-3.5916742619	-0.6536268611
C	0.2977348920	-3.0474549220	1.4215163482
H	-0.7907564263	-3.1522712327	1.3750041059
H	0.7002229006	-4.0173307201	1.7497169579
C	0.6264320093	-1.9819520009	2.4766515028
H	0.2382035516	-2.3203752409	3.4431898360
H	0.0872628979	-1.0582341271	2.2406342611
C	4.2974094989	-1.3731997526	1.1618966376
H	4.8631458839	-0.4993135622	1.5006984303
H	4.6174068901	-2.2224093903	1.7721464819
H	-1.7872666198	-2.7645295266	-1.3623393659
H	-3.6478424628	-3.5553099686	-2.8351083887
H	3.2337911428	1.0473183803	-2.7487073641
H	5.0725005275	2.6029328856	-3.3425372851
H	6.4360437317	3.6630215838	-1.5538498713
H	1.2568066593	0.2404213156	1.2049399448

TS1i

0 1

C	2.8591553740	-1.1288555992	1.2851993960
N	2.2260994795	-0.0000236800	0.8908999796
N	2.4779894633	0.4428068926	-0.3849574683
H	1.5581136528	0.6629242429	-0.8445763423
O	-0.0961855867	0.7316536480	-1.1968874874
C	3.2376475549	-1.6861803780	-1.0647192762
C	2.4031174256	-2.4010674425	-0.2280961692
H	2.8730765641	-3.1533540835	0.4091749354
H	2.9524731280	-1.3818808018	-2.0679686187
O	-0.3551196851	0.6992419991	1.3762162684

P	-0.9811420873	0.6814592151	0.0122146404
O	-1.9210885248	-0.6698014786	-0.0227613016
O	-2.1173878295	1.8361569802	-0.1623702006
C	-2.8850789093	-0.8041443202	-0.9959635118
C	-3.2791715177	1.7806946753	0.5770010541
C	-4.0854002936	-0.0886529395	-0.8849456480
C	-2.6788765436	-1.7164819942	-2.0239011018
C	-4.2892555414	0.8784899549	0.2162067658
C	-3.4551141935	2.6910164551	1.6119773767
C	-5.0825901402	-0.3333355163	-1.8342281394
C	-3.6844243490	-1.9420691148	-2.9572005433
C	-5.4957558762	0.9325320175	0.9217134678
C	-4.6625774616	2.7236771543	2.3002782374
H	-2.6382163616	3.3629645882	1.8510822886
C	-4.8911318117	-1.2521277209	-2.8600304349
C	-5.6873446296	1.8459994627	1.9518929916
H	-4.8023488503	3.4342966584	3.1088032574
H	-5.6771102888	-1.4203046162	-3.5891516161
H	-6.0116151511	0.2263109544	-1.7691300665
H	-6.2819092846	0.2285109464	0.6629429394
H	-6.6301389654	1.8654520674	2.4890343736
C	3.4757713817	1.4285323993	-0.5920114164
C	4.2151032237	1.9895185872	0.4488995673
C	3.7210065157	1.8134179153	-1.9141761033
C	5.2166347649	2.9132672111	0.1584244938
H	3.9760109217	1.7276620397	1.4741666159
C	4.7142527928	2.7440485090	-2.1884064460
C	5.4748092694	3.2913329615	-1.1551166885
H	5.7865889099	3.3507539157	0.9722795656
C	4.6013075306	-1.3881483783	-0.5094426580
H	5.0034848680	-0.4690760088	-0.9411140934
H	5.3151799492	-2.1884382992	-0.7382300670
C	2.3232583702	-1.6989877671	2.5839966863
C	0.9273429400	-2.5184560640	-0.4959668705
H	0.4988886694	-1.5122653211	-0.4207180103
H	0.7671694658	-2.8058773955	-1.5434714429
C	0.1624875514	-3.4793963123	0.4229174453
H	0.2071778352	-4.4968201706	0.0161670480
H	-0.8908883482	-3.1784436020	0.4063436217
C	0.6417917414	-3.5325774304	1.8809224764
H	1.5673023546	-4.1221421056	1.9493724546
C	4.3663007237	-1.2580172922	1.0144506588
H	4.9008507072	-0.4052663974	1.4466763242
H	4.7145464603	-2.1531275940	1.5367657405
H	-1.7270078104	-2.2359045198	-2.0691607173
H	-3.5245682672	-2.6537749843	-3.7611021266
H	3.1224839619	1.3822073713	-2.7121805976
H	4.8957905893	3.0431235414	-3.2159018224
H	6.2525002006	4.0154910863	-1.3733640313
H	1.2439892620	0.1903367489	1.2062225782
C	0.8575415304	-2.1753746688	2.5761275328
H	-0.1034082807	-4.1038619508	2.4444973431
H	0.5638861080	-2.2683003243	3.6264742975
H	0.1972488900	-1.4092951130	2.1507044784
H	2.9685872383	-2.5315477607	2.8858868193
H	2.4496412196	-0.9183320530	3.3453822978

TS1j

0 1

C	-2.9253715523	-0.9114714185	-1.7445739085
N	-2.1825823364	0.1267775404	-1.2746119072
N	-2.2706818057	0.3523364573	0.0765288461
H	-1.3028559152	0.4212943520	0.4987328319
H	-1.2003423695	0.2210967037	-1.6077090771
O	0.2480347250	0.3569463118	1.0028258072
C	-2.9692173228	-1.7671453592	0.5592639440
C	-2.2707597846	-2.3918796847	-0.4593020116
H	-2.8340508224	-3.0620406529	-1.1143753533
H	-2.5487279447	-1.6197011747	1.5504543891
O	0.5522117880	0.3549797574	-1.5694220810
P	1.1527583043	0.3074301137	-0.1943359372
O	2.0880350637	-1.0417416629	-0.1605948452
O	2.2898852727	1.4575078625	0.0166875164
C	3.0355766869	-1.1958893758	0.8262112953
C	3.4642515009	1.4088176923	-0.7025307529
C	4.2398115823	-0.4831020568	0.7470804270
C	2.8086137399	-2.1268434048	1.8332452664
C	4.4657181981	0.4991939325	-0.3361113246
C	3.6603312341	2.3310530644	-1.7233015816
C	5.2196796402	-0.7481017855	1.7089342136
C	3.7980031611	-2.3728534185	2.7788390888
H	1.8559934897	-2.6470788906	1.8514056282
C	5.6843538606	0.5586075895	-1.0199254420
C	4.8795379679	2.3689016299	-2.3903305677
H	2.8494026182	3.0081562811	-1.9681142860
C	5.0076494338	-1.6844550516	2.7147362904
H	3.6229667527	-3.0993821874	3.5660393570
C	5.8960049947	1.4842571968	-2.0352030061
H	5.0349516707	3.0888807179	-3.1876347151
H	5.7806760428	-1.8684194503	3.4538048751
H	6.1515894610	-0.1908920243	1.6697409915
H	6.4639243830	-0.1510505205	-0.7567295888
H	6.8478625937	1.5075655797	-2.5559086389
C	-3.1655356053	1.3332143882	0.5654646808
C	-4.0224175850	2.0649123513	-0.2563969032
C	-3.1763046522	1.5324679207	1.9503380174
C	-4.9110711613	2.9716370092	0.3174444039
H	-3.9591638668	1.9488639777	-1.3331413828
C	-4.0611314273	2.4458966549	2.5066096024
H	-2.4764331097	0.9779650509	2.5699884314
C	-4.9409619335	3.1624747539	1.6950317127
H	-5.5736374696	3.5432484179	-0.3251240463
H	-4.0613526788	2.6016026185	3.5807887512
H	-5.6321624842	3.8743865603	2.1335088028
C	-4.3928419177	-1.4035506832	0.2495473821
H	-4.7230498614	-0.5628004481	0.8632508660
H	-5.0741134695	-2.2379430620	0.4534215639
C	-4.3739595089	-1.0481808210	-1.2558902643
H	-4.9298329713	-0.1288899388	-1.4726950680
H	-4.8361607655	-1.8447078598	-1.8447929250

C	-0.7741383163	-2.5596059967	-0.3928832630
H	-0.3344573780	-1.8785900030	-1.1288723889
H	-0.4155546392	-2.1764433072	0.5703892628
C	-0.2342471705	-3.9764559322	-0.6437003454
H	-0.1887832617	-4.5257406671	0.3044725436
C	-2.7008428590	-1.2220502289	-3.2150819263
H	-3.0815070710	-0.3422136552	-3.7517314436
H	-3.3655368842	-2.0491806631	-3.4890578193
C	-1.2661900333	-1.5550558564	-3.6715614527
H	-0.5216876778	-1.0365699742	-3.0578332123
H	-1.1247194233	-1.1714917033	-4.6878413035
C	0.4352630464	-3.5107246558	-3.7559686810
C	-1.0320808002	-3.0708602574	-3.6750711640
H	-1.5036952450	-3.5054267897	-2.7912276479
H	-1.5740117108	-3.4952460642	-4.5312825876
C	1.1603701023	-3.9772187612	-1.2898654814
H	1.8874106233	-3.6069431607	-0.5568918984
H	1.4372267601	-5.0179092329	-1.5057808355
C	1.3352658101	-3.1397601061	-2.5677436624
H	1.2499203105	-2.0713969528	-2.3375052513
H	2.3760408945	-3.2731493171	-2.8865514223
H	0.8674159480	-3.0752848709	-4.6667960932
H	0.4630147567	-4.6000764574	-3.8982148221
H	-0.9312796263	-4.5443569417	-1.2709684720

TS1k

0 1

C	-2.6172242058	-0.8367773526	-1.0355536541
N	-1.9378951831	0.3286832368	-0.9149742796
N	-2.0694322946	1.0771303602	0.2299892063
H	-1.1103132582	1.2475790752	0.6081049639
O	0.5015256905	0.8561622164	1.0584869249
C	-2.2019176065	-0.8210506637	1.6718720835
C	-1.9942292937	-1.8069507927	0.7253251344
H	-2.8021856962	-2.5256416616	0.6075317758
H	-1.3192478559	-0.3439568543	2.0972176921
O	0.7437706938	0.1921762525	-1.4305891646
P	1.3802506625	0.5028404790	-0.1061340316
O	2.3432501292	-0.7759488428	0.2511940447
O	2.4975408786	1.6864202719	-0.2412808843
C	3.3545440124	-0.6480416337	1.1765182824
C	3.6258217540	1.4701280739	-1.0014985923
C	4.5336273459	0.0286055938	0.8329253685
C	3.2172553349	-1.2790985813	2.4074444909
C	4.6701811403	0.6944437019	-0.4811354718
C	3.7351603113	2.1001979419	-2.2351058712
C	5.5774205930	0.0382221544	1.7635902655
C	4.2681489191	-1.2546330231	3.3173165625
H	2.2845725401	-1.7885498539	2.6256254006
C	5.8429030170	0.5853941148	-1.2354128296
C	4.9098645364	1.9751406824	-2.9681513094
H	2.8932589674	2.6828008908	-2.5925171448
C	5.4533120767	-0.5974570276	2.9937032209
H	4.1605810171	-1.7475427032	4.2784782879

C	5.9681151821	1.2200904408	-2.4658588069
H	4.9979935980	2.4664768951	-3.9320536197
H	6.2750264874	-0.5707815531	3.7020498342
H	6.4898809122	0.5733055171	1.5153088958
H	6.6548225698	-0.0257830734	-0.8508188968
H	6.8850989480	1.1164587709	-3.0370018071
C	-2.9044415184	2.2179795780	0.2232450354
C	-2.9559919652	2.9804178737	1.3944589466
C	-3.6763150941	2.5818644828	-0.8816252879
C	-3.7904324657	4.0895143221	1.4603757185
H	-2.3299466540	2.7006032347	2.2379770973
C	-4.5144640414	3.6897168939	-0.7981708399
H	-3.5999090226	2.0047059771	-1.7973855270
C	-4.5805296827	4.4459341430	0.3689483091
H	-3.8210847609	4.6792375252	2.3711663537
H	-5.1109138092	3.9693714131	-1.6612086888
H	-5.2321125870	5.3114709546	0.4243094825
C	-4.1301020615	-0.9225256089	-0.9063437966
H	-4.5013486383	-0.4489727403	-1.8296267052
H	-4.3820573500	-1.9844766642	-1.0026389808
C	-0.5806132100	-2.3128307491	0.4760299483
H	-0.1049517600	-1.7975438022	-0.3659321343
H	0.0331827917	-2.0410795473	1.3413449362
C	-0.4918992817	-3.8272444196	0.2589085454
H	-1.1001956493	-4.3402052520	1.0174214132
H	0.5457433387	-4.1286659033	0.4407093021
C	-0.8861563804	-4.3416477795	-1.1312434169
H	-0.6491407386	-5.4107312845	-1.1611772562
H	-0.2353758368	-3.8726072098	-1.8818880540
C	-2.3614710625	-4.1524682836	-1.5398065415
H	-2.7206393446	-5.0692111267	-2.0206926576
H	-2.9903545681	-4.0365532619	-0.6490401474
C	-2.0565749025	-1.6265894992	-2.2164921864
H	-0.9648117185	-1.6508306109	-2.1304827073
H	-2.2544407612	-0.9918562099	-3.0912391064
C	-2.6080248515	-3.0174707020	-2.5417513611
H	-3.6785185653	-2.9550741246	-2.7695909026
H	-2.1263167748	-3.3053616162	-3.4842869262
C	-4.7432208702	-1.0488371689	1.6263660047
H	-4.7186573139	-2.1403187892	1.5064205744
H	-5.6156012254	-0.8347868579	2.2528339281
C	-3.5028838261	-0.5550020315	2.3632762816
H	-3.4439641010	-1.0174107157	3.3598573319
H	-3.6002909181	0.5236094866	2.5426833688
C	-4.9459446075	-0.3682999918	0.2708211522
H	-4.8101944698	0.7095499513	0.3826877735
H	-5.9915631012	-0.5073918410	-0.0278345387
H	-0.9423405638	0.2902599026	-1.2375248112

3

0 1

C	0	3.2389420000	0.8066680000	-1.0872740000

N	0	2.3242610000	-0.0613660000	-1.2717130000
N	0	2.5531860000	-1.4207890000	-1.2959290000
H	0	1.6798210000	-1.8648310000	-1.0086850000
H	0	0.7695520000	0.3239070000	-1.3466610000
O	0	-0.0507660000	-1.8089930000	-0.0817340000
O	0	-0.2448900000	0.4913460000	-1.2458910000
P	0	-0.9146700000	-0.6657390000	-0.4348320000
O	0	-1.5843320000	0.1258700000	0.8011690000
O	0	-2.2039520000	-1.1445550000	-1.2724490000
C	0	-2.6294300000	-0.4651410000	1.4975720000
C	0	-3.2668660000	-0.2799250000	-1.4902200000
C	0	-3.9064320000	-0.4944200000	0.9260770000
C	0	-2.3860490000	-0.9350370000	2.7804890000
C	0	-4.1430400000	0.0198460000	-0.4409860000
C	0	-3.4728310000	0.1940660000	-2.7783300000
C	0	-4.9505100000	-1.0096900000	1.7014960000
C	0	-3.4409220000	-1.4437770000	3.5301080000
H	0	-1.3747980000	-0.8862780000	3.1689470000
C	0	-5.2569870000	0.8134520000	-0.7350250000
C	0	-4.5870430000	0.9822220000	-3.0440370000
H	0	-2.7587820000	-0.0721120000	-3.5498250000
C	0	-4.7252630000	-1.4779270000	2.9907660000
H	0	-3.2584650000	-1.8143230000	4.5335280000
C	0	-5.4823010000	1.2892060000	-2.0214040000
H	0	-4.7542760000	1.3560780000	-4.0489090000
H	0	-5.5498440000	-1.8792260000	3.5706830000
H	0	-5.9457150000	-1.0575250000	1.2688160000
H	0	-5.9389420000	1.0716610000	0.0700930000
H	0	-6.3503700000	1.9079800000	-2.2235680000
C	0	3.6807380000	-1.9653170000	-0.6174570000
C	0	4.9514790000	-1.8986340000	-1.1918930000

C	0	3.4997250000	-2.6117190000	0.6064110000
C	0	6.0405390000	-2.4561230000	-0.5292690000
H	0	5.0685520000	-1.4304030000	-2.1649860000
C	0	4.5892170000	-3.1895330000	1.2513050000
H	0	2.5011670000	-2.6556470000	1.0339790000
C	0	5.8617810000	-3.1044290000	0.6912140000
H	0	7.0281140000	-2.4013880000	-0.9767630000
H	0	4.4443190000	-3.7000470000	2.1983590000
H	0	6.7112700000	-3.5490570000	1.1998270000
H	0	4.2731650000	0.5127380000	-0.8950290000
H	0	2.9979203100	1.8491656700	-1.0846009300

4

0 1

C	0	2.1559160000	-3.5282150000	-0.1923850000
C	0	1.0723920000	-2.9788490000	0.3622690000
H	0	0.5609800000	-2.1921850000	-0.1948280000
H	0	2.7066960000	-4.3101740000	0.3370580000
H	0	2.5956892800	-3.1147411400	-1.0758660900
H	0	0.7763317900	-3.1025812100	1.3830229800

5

0 1

C	3.2397084145	-1.9147968920	-2.2946652975
N	2.9197882448	-1.8859877849	-0.8643341116
N	2.7999198705	-0.4914750911	-0.5417585327
H	1.5296396552	-0.2538730149	0.3817297827

O	0.5953840586	-0.0584804948	0.7929297558
C	2.4552988147	0.2701028767	-1.7951405650
C	2.3890512844	-0.7931820391	-2.8964525626
H	2.7712525792	-0.4167243217	-3.8479676117
H	1.5034077445	0.7936284197	-1.6587319424
O	-0.0620131609	-1.7864701731	-1.0107919702
P	-0.5100355971	-0.7259627800	-0.0878206934
O	-1.2545575183	0.4506911449	-0.9114722748
O	-1.6060538887	-1.1222356358	1.0206194326
C	-1.9483771965	1.4441733729	-0.2364966906
C	-2.9238008110	-1.3260077906	0.6320886889
C	-3.1857760826	1.1516223945	0.3482228254
C	-1.4221123412	2.7285196474	-0.2348454118
C	-3.7326026879	-0.2232331141	0.3367354106
C	-3.4176671658	-2.6227423782	0.6340447227
C	-3.8925635467	2.2085395761	0.9314324506
C	-2.1434801590	3.7618552010	0.3522867462
H	-0.4584616452	2.8971485125	-0.7036561185
C	-5.0792062395	-0.4681135274	0.0477816133
C	-4.7597579985	-2.8392465361	0.3417793341
H	-2.7417213091	-3.4364993942	0.8723496349
C	-3.3826290568	3.5015303426	0.9335236160
H	-1.7367878793	4.7678477753	0.3568477416
C	-5.5919763955	-1.7600311030	0.0516350876
H	-5.1534307878	-3.8503634386	0.3399864070
H	-3.9467460750	4.3034304989	1.3981303974
H	-4.8463414099	1.9970069121	1.4062331953
H	-5.7194217884	0.3731749098	-0.2017484228
H	-6.6385265953	-1.9256280218	-0.1819404336
C	3.9325466531	0.0251721859	0.1754753019
C	4.9761350610	-0.7952087194	0.6003632295

C	3.9418862891	1.3862515617	0.4943819271
C	6.0331133597	-0.2426365336	1.3190727330
H	4.9400562741	-1.8521948711	0.3683205864
C	5.0028172077	1.9253554877	1.2123983991
H	3.1074175072	2.0152840758	0.1956083299
C	6.0573072368	1.1145903487	1.6253353202
H	6.8456127679	-0.8862825148	1.6425509115
H	4.9984547336	2.9830987170	1.4567366453
H	6.8842353698	1.5358030135	2.1875937596
H	1.9716475285	-2.2542857945	-0.7582358053
H	1.3608781070	-1.1378603906	-3.0278128060
H	3.2379245655	1.0091461760	-1.9868728707
H	4.3104434171	-1.7139871290	-2.4194036540
H	3.0169429317	-2.9049953864	-2.6968407908

6a

0 1

C	0	-2.8060080000	1.7454320000	0.9120920000
N	0	-2.6265490000	0.5205030000	0.5588900000
N	0	-3.0201270000	0.0630520000	-0.7112840000
H	0	-2.1561270000	0.1266740000	-1.2826200000
H	0	-1.9082250000	-0.1123130000	1.0341220000
O	0	-0.3695050000	0.0328410000	-1.4813090000
C	0	-1.1959490000	3.1556550000	-0.5372300000
C	0	-0.9270260000	3.9020850000	0.5346800000
H	0	-1.6117240000	4.7173860000	0.7839010000
H	0	-0.5596360000	2.3047870000	-0.7891810000
O	0	-0.4595230000	-0.8343650000	0.9687280000
P	0	0.3359700000	-0.4462980000	-0.2505290000

O	0	1.4002430000	0.6966810000	0.2743280000
O	0	1.3596730000	-1.6313210000	-0.6912900000
C	0	2.5257020000	0.9865270000	-0.4680210000
C	0	2.3821400000	-2.0021570000	0.1573400000
C	0	3.6076550000	0.0948420000	-0.4615370000
C	0	2.5956140000	2.2064370000	-1.1311970000
C	0	3.5239160000	-1.1966020000	0.2564380000
C	0	2.2856620000	-3.2143620000	0.8290260000
C	0	4.7715270000	0.4757120000	-1.1367980000
C	0	3.7640960000	2.5616590000	-1.7966310000
H	0	1.7302050000	2.8622260000	-1.1130220000
C	0	4.5828780000	-1.6606620000	1.0439220000
C	0	3.3506400000	-3.6529740000	1.6076550000
H	0	1.3746470000	-3.7926260000	0.7210100000
C	0	4.8558500000	1.6964500000	-1.7968780000
H	0	3.8192010000	3.5129440000	-2.3163260000
C	0	4.5036170000	-2.8772010000	1.7119370000
H	0	3.2789760000	-4.5996600000	2.1337380000
H	0	5.7675750000	1.9668830000	-2.3195700000
H	0	5.6114030000	-0.2132920000	-1.1542310000
H	0	5.4691990000	-1.0400420000	1.1429050000
H	0	5.3355680000	-3.2140380000	2.3219490000
C	0	-3.4529430000	-1.2955190000	-0.6585460000
C	0	-4.4384520000	-1.6844530000	0.2477910000
C	0	-2.9154890000	-2.2165830000	-1.5578030000
C	0	-4.8854840000	-3.0015110000	0.2535110000
H	0	-4.8547400000	-0.9542490000	0.9362200000
C	0	-3.3848520000	-3.5255660000	-1.5561630000
H	0	-2.1199330000	-1.9033680000	-2.2284700000
C	0	-4.3668530000	-3.9240290000	-0.6516670000
H	0	-5.6496520000	-3.3044990000	0.9625850000

H	0	-2.9650480000	-4.2423940000	-2.2548020000
H	0	-4.7221330000	-4.9493040000	-0.6484310000
C	0	-2.5801470000	3.1679380000	-1.1483700000
H	0	-2.6390310000	2.4926230000	-2.0041980000
H	0	-2.8940620000	4.1636780000	-1.4767000000
C	0	-3.5451840000	2.6800610000	-0.0201410000
H	0	-4.4140850000	2.1613950000	-0.4344860000
H	0	-3.8783770000	3.5390030000	0.5679700000
H	0	-2.4064772800	2.0409098500	1.8597037600
H	0	-0.2754365400	3.5730299600	1.3170187800

6b

0 1

C	0	3.6335510000	-0.0025420000	0.5500630000
N	0	2.5363990000	0.5934110000	0.2471240000
N	0	2.3455760000	1.1835380000	-1.0221440000
H	0	1.6936150000	0.5367530000	-1.4975950000
O	0	0.1972830000	-0.5785220000	-1.3420050000
C	0	3.0508080000	-2.3967440000	-0.8172130000
C	0	3.0183570000	-2.9834430000	0.3797160000
H	0	3.9306190000	-3.4551760000	0.7585430000
H	0	2.1405370000	-1.9278760000	-1.2025190000
O	0	0.1061900000	0.3542250000	1.0735750000
P	0	-0.6047260000	-0.2058920000	-0.1352330000
O	0	-1.4931590000	-1.4563090000	0.4280650000
O	0	-1.7846400000	0.8033930000	-0.6370600000
C	0	-2.5845450000	-1.9135050000	-0.2792260000
C	0	-2.8306250000	1.0885940000	0.2145490000
C	0	-3.7766440000	-1.1751860000	-0.2793680000

C	0	-2.4980800000	-3.1521290000	-0.9030290000
C	0	-3.8546230000	0.1474590000	0.3804640000
C	0	-2.8769770000	2.3362550000	0.8235990000
C	0	-4.8893900000	-1.7350560000	-0.9154640000
C	0	-3.6176550000	-3.6861080000	-1.5310030000
H	0	-1.5489830000	-3.6767420000	-0.8760890000
C	0	-4.9507560000	0.5103670000	1.1696250000
C	0	-3.9764490000	2.6721510000	1.6057360000
H	0	-2.0474090000	3.0180280000	0.6646110000
C	0	-4.8178340000	-2.9784530000	-1.5332700000
H	0	-3.5516390000	-4.6532820000	-2.0192380000
C	0	-5.0169100000	1.7601160000	1.7753280000
H	0	-4.0196200000	3.6450590000	2.0852270000
H	0	-5.6935090000	-3.3886980000	-2.0257870000
H	0	-5.8154200000	-1.1671360000	-0.9376070000
H	0	-5.7472510000	-0.2130290000	1.3208570000
H	0	-5.8739880000	2.0178390000	2.3891820000
C	0	1.7062090000	2.4563620000	-0.8906540000
C	0	2.2383520000	3.4249120000	-0.0400440000
C	0	0.5715950000	2.7340460000	-1.6527110000
C	0	1.6290670000	4.6713730000	0.0509160000
H	0	3.1250520000	3.1982480000	0.5458690000
C	0	-0.0209370000	3.9897020000	-1.5656840000
H	0	0.1404040000	1.9564730000	-2.2770940000
C	0	0.5010160000	4.9608610000	-0.7140480000
H	0	2.0419890000	5.4216860000	0.7180590000
H	0	-0.9093490000	4.1986390000	-2.1534860000
H	0	0.0300210000	5.9359430000	-0.6433400000
C	0	4.3090300000	-2.2165520000	-1.6134410000
H	0	4.2034450000	-2.6291630000	-2.6240790000
H	0	5.1367150000	-2.7503440000	-1.1286190000

C	0	4.8120680000	0.0093290000	-0.3926690000
H	0	5.0216300000	1.0638470000	-0.6109580000
H	0	5.6719120000	-0.4009320000	0.1465320000
C	0	4.6458820000	-0.7240540000	-1.7413640000
H	0	3.8695180000	-0.2170440000	-2.3183450000
H	0	5.5853350000	-0.6008440000	-2.2913140000
H	0	1.6154900000	0.5357440000	0.8114640000
H	0	3.7648937500	-0.3733572600	1.5451233600
H	0	2.1820910000	-2.9829537200	1.0472180200

6c

0 1

C	0	2.3604790000	-1.9046850000	-0.5560380000
N	0	1.4512450000	-1.0062640000	-0.5573120000
N	0	1.1367810000	-0.4116240000	-1.8090320000
H	0	0.3582200000	-0.9520300000	-2.1980150000
O	0	-1.4853550000	-1.6054260000	-1.5526370000
C	0	5.3918440000	1.1497760000	-0.2891870000
C	0	6.1333700000	0.7705870000	0.7509630000
H	0	7.0212790000	0.1574890000	0.5762570000
H	0	4.5040340000	1.7594390000	-0.1001870000
O	0	-0.7933230000	-0.6416170000	0.7612140000
P	0	-1.8968550000	-0.9083200000	-0.3196500000
O	0	-3.0241020000	-1.6229220000	0.5846520000
O	0	-2.5890680000	0.5021180000	-0.6746880000
C	0	-4.3658790000	-1.5371610000	0.2439580000
C	0	-3.1359450000	1.2624340000	0.3475220000
C	0	-5.0610120000	-0.3352420000	0.4299560000
C	0	-5.0028820000	-2.6936840000	-0.1850130000

C	0	-4.3720320000	0.8925600000	0.8862590000
C	0	-2.4606270000	2.4049310000	0.7514290000
C	0	-6.4365080000	-0.3387930000	0.1744590000
C	0	-6.3706380000	-2.6692890000	-0.4333880000
H	0	-4.4126500000	-3.5950760000	-0.3079190000
C	0	-4.9336270000	1.7351510000	1.8516240000
C	0	-3.0403950000	3.2239990000	1.7139170000
H	0	-1.5005630000	2.6282260000	0.2956470000
C	0	-7.0895180000	-1.4902190000	-0.2495170000
H	0	-6.8724820000	-3.5705110000	-0.7702660000
C	0	-4.2787730000	2.8911240000	2.2609800000
H	0	-2.5232950000	4.1213510000	2.0383580000
H	0	-8.1562010000	-1.4643590000	-0.4466250000
H	0	-6.9886080000	0.5889810000	0.2946560000
H	0	-5.8856960000	1.4592040000	2.2960710000
H	0	-4.7295440000	3.5267260000	3.0162240000
C	0	0.7705310000	0.9457870000	-1.6824850000
C	0	1.3867600000	1.7744410000	-0.7394870000
C	0	-0.1769250000	1.4844370000	-2.5604090000
C	0	1.0784370000	3.1322330000	-0.7020340000
H	0	2.0975320000	1.3480980000	-0.0381050000
C	0	-0.4744490000	2.8397520000	-2.5146280000
H	0	-0.6889710000	0.8277780000	-3.2585270000
C	0	0.1569470000	3.6758420000	-1.5930880000
H	0	1.5668310000	3.7671190000	0.0316210000
H	0	-1.2158520000	3.2438340000	-3.1969130000
H	0	-0.0782980000	4.7347660000	-1.5625650000
C	0	3.2043860000	-2.3009150000	-1.7404210000
H	0	3.3803860000	-3.3804050000	-1.6799630000
H	0	2.6920820000	-2.0848560000	-2.6793330000
C	0	4.4511830000	-0.1147350000	-2.2178720000

H	0	3.5036120000	0.3393850000	-1.9044650000
H	0	4.4263190000	-0.1408980000	-3.3133810000
C	0	5.6074130000	0.7711180000	-1.7252810000
H	0	5.6553130000	1.6765000000	-2.3421260000
H	0	6.5655350000	0.2483160000	-1.8419790000
C	0	4.5478810000	-1.5445270000	-1.6817880000
H	0	4.9052890000	-1.5177190000	-0.6425840000
H	0	5.3002480000	-2.0999450000	-2.2532260000
H	0	0.1537610000	-0.7557100000	0.3867300000
H	0	5.9056276100	1.0088393800	1.7689360900
H	0	2.5463838800	-2.4292548000	0.3578361200

8a

0 1

C	0	-2.8060080000	1.7454320000	0.9120920000
N	0	-2.6265490000	0.5205030000	0.5588900000
N	0	-3.0201270000	0.0630520000	-0.7112840000
H	0	-2.1561270000	0.1266740000	-1.2826200000
H	0	-1.9082250000	-0.1123130000	1.0341220000
O	0	-0.3695050000	0.0328410000	-1.4813090000
C	0	-1.1959490000	3.1556550000	-0.5372300000
C	0	-0.9270260000	3.9020850000	0.5346800000
H	0	-1.6117240000	4.7173860000	0.7839010000
H	0	-0.5596360000	2.3047870000	-0.7891810000
O	0	-0.4595230000	-0.8343650000	0.9687280000
P	0	0.3359700000	-0.4462980000	-0.2505290000
O	0	1.4002430000	0.6966810000	0.2743280000
O	0	1.3596730000	-1.6313210000	-0.6912900000
C	0	2.5257020000	0.9865270000	-0.4680210000

C	0	2.3821400000	-2.0021570000	0.1573400000
C	0	3.6076550000	0.0948420000	-0.4615370000
C	0	2.5956140000	2.2064370000	-1.1311970000
C	0	3.5239160000	-1.1966020000	0.2564380000
C	0	2.2856620000	-3.2143620000	0.8290260000
C	0	4.7715270000	0.4757120000	-1.1367980000
C	0	3.7640960000	2.5616590000	-1.7966310000
H	0	1.7302050000	2.8622260000	-1.1130220000
C	0	4.5828780000	-1.6606620000	1.0439220000
C	0	3.3506400000	-3.6529740000	1.6076550000
H	0	1.3746470000	-3.7926260000	0.7210100000
C	0	4.8558500000	1.6964500000	-1.7968780000
H	0	3.8192010000	3.5129440000	-2.3163260000
C	0	4.5036170000	-2.8772010000	1.7119370000
H	0	3.2789760000	-4.5996600000	2.1337380000
H	0	5.7675750000	1.9668830000	-2.3195700000
H	0	5.6114030000	-0.2132920000	-1.1542310000
H	0	5.4691990000	-1.0400420000	1.1429050000
H	0	5.3355680000	-3.2140380000	2.3219490000
C	0	-3.4529430000	-1.2955190000	-0.6585460000
C	0	-4.4384520000	-1.6844530000	0.2477910000
C	0	-2.9154890000	-2.2165830000	-1.5578030000
C	0	-4.8854840000	-3.0015110000	0.2535110000
H	0	-4.8547400000	-0.9542490000	0.9362200000
C	0	-3.3848520000	-3.5255660000	-1.5561630000
H	0	-2.1199330000	-1.9033680000	-2.2284700000
C	0	-4.3668530000	-3.9240290000	-0.6516670000
H	0	-5.6496520000	-3.3044990000	0.9625850000
H	0	-2.9650480000	-4.2423940000	-2.2548020000
H	0	-4.7221330000	-4.9493040000	-0.6484310000
C	0	-2.2395040000	2.1643970000	2.2557330000

H	0	-2.6888280000	3.1218520000	2.5345780000
H	0	-2.5764940000	1.4200160000	2.9867320000
C	0	-0.0123890000	3.4401900000	1.6328500000
H	0	0.9532200000	3.1021040000	1.2431770000
H	0	0.1784360000	4.2477310000	2.3452190000
C	0	-0.6866590000	2.2316750000	2.3474610000
H	0	-0.4317600000	2.2399600000	3.4117350000
H	0	-0.2826380000	1.3007900000	1.9405010000
H	0	-2.1747574800	3.1643406800	-0.9693856700
H	0	-3.3287790100	2.4064341800	0.2527843500

8b

0 1

C	0	3.6335510000	-0.0025420000	0.5500630000
N	0	2.5363990000	0.5934110000	0.2471240000
N	0	2.3455760000	1.1835380000	-1.0221440000
H	0	1.6936150000	0.5367530000	-1.4975950000
O	0	0.1972830000	-0.5785220000	-1.3420050000
C	0	3.0508080000	-2.3967440000	-0.8172130000
C	0	3.0183570000	-2.9834430000	0.3797160000
H	0	3.9306190000	-3.4551760000	0.7585430000
H	0	2.1405370000	-1.9278760000	-1.2025190000
O	0	0.1061900000	0.3542250000	1.0735750000
P	0	-0.6047260000	-0.2058920000	-0.1352330000
O	0	-1.4931590000	-1.4563090000	0.4280650000
O	0	-1.7846400000	0.8033930000	-0.6370600000
C	0	-2.5845450000	-1.9135050000	-0.2792260000
C	0	-2.8306250000	1.0885940000	0.2145490000
C	0	-3.7766440000	-1.1751860000	-0.2793680000

C	0	-2.4980800000	-3.1521290000	-0.9030290000
C	0	-3.8546230000	0.1474590000	0.3804640000
C	0	-2.8769770000	2.3362550000	0.8235990000
C	0	-4.8893900000	-1.7350560000	-0.9154640000
C	0	-3.6176550000	-3.6861080000	-1.5310030000
H	0	-1.5489830000	-3.6767420000	-0.8760890000
C	0	-4.9507560000	0.5103670000	1.1696250000
C	0	-3.9764490000	2.6721510000	1.6057360000
H	0	-2.0474090000	3.0180280000	0.6646110000
C	0	-4.8178340000	-2.9784530000	-1.5332700000
H	0	-3.5516390000	-4.6532820000	-2.0192380000
C	0	-5.0169100000	1.7601160000	1.7753280000
H	0	-4.0196200000	3.6450590000	2.0852270000
H	0	-5.6935090000	-3.3886980000	-2.0257870000
H	0	-5.8154200000	-1.1671360000	-0.9376070000
H	0	-5.7472510000	-0.2130290000	1.3208570000
H	0	-5.8739880000	2.0178390000	2.3891820000
C	0	1.7062090000	2.4563620000	-0.8906540000
C	0	2.2383520000	3.4249120000	-0.0400440000
C	0	0.5715950000	2.7340460000	-1.6527110000
C	0	1.6290670000	4.6713730000	0.0509160000
H	0	3.1250520000	3.1982480000	0.5458690000
C	0	-0.0209370000	3.9897020000	-1.5656840000
H	0	0.1404040000	1.9564730000	-2.2770940000
C	0	0.5010160000	4.9608610000	-0.7140480000
H	0	2.0419890000	5.4216860000	0.7180590000
H	0	-0.9093490000	4.1986390000	-2.1534860000
H	0	0.0300210000	5.9359430000	-0.6433400000
C	0	3.8185670000	-0.5248910000	1.9517550000
H	0	4.5487100000	-1.3388640000	1.9089290000
H	0	4.3332900000	0.3040240000	2.4631110000

C	0	1.8458630000	-2.9827570000	1.3155930000
H	0	1.0238890000	-2.3923010000	0.8941070000
H	0	1.4697420000	-4.0062600000	1.4444770000
C	0	2.2345030000	-2.4431080000	2.7005880000
H	0	1.4020010000	-2.6168340000	3.3902890000
H	0	3.0830300000	-3.0287570000	3.0834920000
C	0	2.5846940000	-0.9508790000	2.7719760000
H	0	2.8033450000	-0.7149300000	3.8186190000
H	0	1.6997210000	-0.3633640000	2.5114040000
H	0	1.6154900000	0.5357440000	0.8114640000
H	0	3.9484253100	-2.2681947800	-1.3852431600
H	0	4.4690830000	0.0058741700	-0.1183047500

8c

0 1

C	0	3.5123530000	0.5066710000	0.6949520000
N	0	2.3409350000	0.8752990000	0.3221410000
N	0	2.1317280000	1.4821340000	-0.9378600000
H	0	1.5907500000	0.7689810000	-1.4513610000
H	0	1.3802030000	0.6613580000	0.8342040000
O	0	0.1939500000	-0.5477030000	-1.2763720000
C	0	3.3702520000	-1.8162610000	-1.2724980000
C	0	3.6900170000	-2.8013010000	-0.4298030000
H	0	4.7401540000	-2.9618130000	-0.1685330000
H	0	2.3200590000	-1.6597820000	-1.5278000000
O	0	-0.0538450000	0.4698820000	1.0854160000
P	0	-0.6841390000	-0.1978880000	-0.1177390000
O	0	-1.4954120000	-1.4856570000	0.4750720000
O	0	-1.9178850000	0.7015430000	-0.6898020000

C	0	-2.5547120000	-2.0344720000	-0.2163190000
C	0	-2.9859570000	0.9654970000	0.1425020000
C	0	-3.7893360000	-1.3699130000	-0.2538040000
C	0	-2.3925630000	-3.2932110000	-0.7823680000
C	0	-3.9530800000	-0.0264080000	0.3450910000
C	0	-3.1061670000	2.2324770000	0.6989400000
C	0	-4.8624980000	-2.0232360000	-0.8684360000
C	0	-3.4742010000	-3.9208170000	-1.3899020000
H	0	-1.4153320000	-3.7607210000	-0.7268320000
C	0	-5.0748010000	0.3050270000	1.1119090000
C	0	-4.2295470000	2.5362910000	1.4600730000
H	0	-2.3134290000	2.9521570000	0.5189840000
C	0	-4.7140290000	-3.2865570000	-1.4291300000
H	0	-3.3475600000	-4.9036780000	-1.8326990000
C	0	-5.2175710000	1.5738180000	1.6621630000
H	0	-4.3319910000	3.5238620000	1.8988290000
H	0	-5.5608010000	-3.7696650000	-1.9058190000
H	0	-5.8203510000	-1.5129380000	-0.9194400000
H	0	-5.8286160000	-0.4566390000	1.2912820000
H	0	-6.0927250000	1.8075460000	2.2599170000
C	0	1.3370430000	2.6645090000	-0.8184660000
C	0	1.6920460000	3.6615350000	0.0892450000
C	0	0.2328770000	2.8321450000	-1.6536710000
C	0	0.9367270000	4.8267280000	0.1616110000
H	0	2.5539800000	3.5207320000	0.7356250000
C	0	-0.5061070000	4.0084590000	-1.5844870000
H	0	-0.0616270000	2.0282980000	-2.3227270000
C	0	-0.1605570000	5.0084290000	-0.6776210000
H	0	1.2114070000	5.5988170000	0.8736210000
H	0	-1.3707950000	4.1305190000	-2.2293930000
H	0	-0.7452810000	5.9208900000	-0.6212170000

C	0	3.6766680000	-0.2452480000	1.9860540000
H	0	4.4023470000	-1.0439730000	1.7801570000
H	0	4.1846450000	0.4427350000	2.6767700000
C	0	2.4074700000	-0.8153910000	2.6277790000
H	0	2.6875230000	-1.2072580000	3.6126650000
H	0	1.6858060000	-0.0116580000	2.8079290000
C	0	1.7359570000	-1.9221690000	1.8073730000
H	0	1.5706220000	-1.5637840000	0.7863290000
H	0	0.7356810000	-2.0918360000	2.2160930000
C	0	2.7139830000	-3.6984870000	0.2754450000
H	0	1.7448890000	-3.6724280000	-0.2370090000
H	0	3.0746740000	-4.7333990000	0.2481990000
C	0	2.5199920000	-3.2456190000	1.7350540000
H	0	3.5074310000	-3.1450450000	2.2089230000
H	0	1.9970640000	-4.0278000000	2.2951970000
H	0	4.0942106300	-1.1699153000	-1.7230766600
H	0	4.3862855800	0.7734616900	0.1382073800

8d

0 1

C	0	2.3604790000	-1.9046850000	-0.5560380000
N	0	1.4512450000	-1.0062640000	-0.5573120000
N	0	1.1367810000	-0.4116240000	-1.8090320000
H	0	0.3582200000	-0.9520300000	-2.1980150000
O	0	-1.4853550000	-1.6054260000	-1.5526370000
C	0	5.3918440000	1.1497760000	-0.2891870000
C	0	6.1333700000	0.7705870000	0.7509630000
H	0	7.0212790000	0.1574890000	0.5762570000
H	0	4.5040340000	1.7594390000	-0.1001870000

O	0	-0.7933230000	-0.6416170000	0.7612140000
P	0	-1.8968550000	-0.9083200000	-0.3196500000
O	0	-3.0241020000	-1.6229220000	0.5846520000
O	0	-2.5890680000	0.5021180000	-0.6746880000
C	0	-4.3658790000	-1.5371610000	0.2439580000
C	0	-3.1359450000	1.2624340000	0.3475220000
C	0	-5.0610120000	-0.3352420000	0.4299560000
C	0	-5.0028820000	-2.6936840000	-0.1850130000
C	0	-4.3720320000	0.8925600000	0.8862590000
C	0	-2.4606270000	2.4049310000	0.7514290000
C	0	-6.4365080000	-0.3387930000	0.1744590000
C	0	-6.3706380000	-2.6692890000	-0.4333880000
H	0	-4.4126500000	-3.5950760000	-0.3079190000
C	0	-4.9336270000	1.7351510000	1.8516240000
C	0	-3.0403950000	3.2239990000	1.7139170000
H	0	-1.5005630000	2.6282260000	0.2956470000
C	0	-7.0895180000	-1.4902190000	-0.2495170000
H	0	-6.8724820000	-3.5705110000	-0.7702660000
C	0	-4.2787730000	2.8911240000	2.2609800000
H	0	-2.5232950000	4.1213510000	2.0383580000
H	0	-8.1562010000	-1.4643590000	-0.4466250000
H	0	-6.9886080000	0.5889810000	0.2946560000
H	0	-5.8856960000	1.4592040000	2.2960710000
H	0	-4.7295440000	3.5267260000	3.0162240000
C	0	0.7705310000	0.9457870000	-1.6824850000
C	0	1.3867600000	1.7744410000	-0.7394870000
C	0	-0.1769250000	1.4844370000	-2.5604090000
C	0	1.0784370000	3.1322330000	-0.7020340000
H	0	2.0975320000	1.3480980000	-0.0381050000
C	0	-0.4744490000	2.8397520000	-2.5146280000
H	0	-0.6889710000	0.8277780000	-3.2585270000

C	0	0.1569470000	3.6758420000	-1.5930880000
H	0	1.5668310000	3.7671190000	0.0316210000
H	0	-1.2158520000	3.2438340000	-3.1969130000
H	0	-0.0782980000	4.7347660000	-1.5625650000
C	0	5.8135770000	1.1051380000	2.1803880000
H	0	6.5843280000	1.7686220000	2.5940350000
H	0	4.8700310000	1.6634590000	2.2127560000
C	0	5.7174910000	-0.1424420000	3.0732120000
H	0	5.3890860000	0.1659090000	4.0736940000
H	0	6.7191130000	-0.5742640000	3.1938190000
C	0	4.7751980000	-1.2236750000	2.5375130000
H	0	5.2021190000	-1.6349050000	1.6135560000
H	0	4.7426660000	-2.0528170000	3.2573180000
C	0	3.3497010000	-0.7318980000	2.2749930000
H	0	3.0075360000	-0.1459320000	3.1381830000
H	0	3.3503430000	-0.0493890000	1.4135850000
C	0	2.6232710000	-2.6462080000	0.7357990000
H	0	3.6571570000	-3.0107050000	0.7162780000
H	0	1.9940170000	-3.5469100000	0.7028250000
C	0	2.3540000000	-1.8691760000	2.0267870000
H	0	1.3316300000	-1.4802170000	2.0164300000
H	0	2.4055660000	-2.5757250000	2.8642300000
H	0	0.1537610000	-0.7557100000	0.3867300000
H	0	2.9595522200	-2.1859610000	-1.3968085300
H	0	5.5455408200	0.8797996500	-1.3130962000

10+11

0 1

C	-3.5636741367	0.2977666210	2.2938582234
N	-2.8424768042	0.1658039461	1.2435106210
N	-3.3793879616	0.5469163183	0.0109575526

H	-2.5844933470	0.7571170714	-0.5991977792
O	-0.6595495822	0.8974223524	-0.9376155284
C	-3.3426525148	3.5952286544	2.1655968101
C	-2.1981904345	3.3291388477	2.7961397461
H	-2.1003779427	3.6125418014	3.8464287854
H	-3.4350028176	3.3091246144	1.1155861392
O	-0.3489353892	-0.5477936182	1.1823551113
P	0.2798831497	0.2131486466	-0.0265705490
O	1.3768573631	1.1595634339	0.6891091074
O	1.2080804974	-0.8346118848	-0.8235851409
C	2.4078367704	1.6836909761	-0.0767389434
C	2.3452291023	-1.3704635595	-0.2379380206
C	3.4801584130	0.8592759169	-0.4347479865
C	2.3767984731	3.0336379155	-0.3967240935
C	3.4874114233	-0.5768374095	-0.0782429237
C	2.3361188002	-2.7187706509	0.0919241041
C	4.5466375174	1.4470719764	-1.1228289793
C	3.4487570675	3.5935136481	-1.0830695562
H	1.5140551423	3.6190935673	-0.0972898298
C	4.6395739097	-1.1941775623	0.4202173006
C	3.4942246735	-3.3079203703	0.5866528163
C	4.5363471317	2.7998734119	-1.4427374143
H	3.4324919189	4.6479921145	-1.3386146701
C	4.6489441151	-2.5449659116	0.7477267155
H	5.5303627411	-0.5902513242	0.5678728050
C	-4.3155162347	-0.3116948347	-0.6147167504
C	-4.8463333028	-1.4553110321	-0.0147970367
C	-4.7295117014	0.0372520010	-1.9072685557
C	-5.7851420051	-2.2267651811	-0.6982322214
H	-4.5079640705	-1.7624488418	0.9689724561
C	-5.6578058454	-0.7429326886	-2.5797524691

H	-4.3144153371	0.9283024190	-2.3697880449
C	-6.1996495801	-1.8792255452	-1.9778938065
H	-5.9646137622	-0.4584081595	-3.5816743850
H	-1.3682231586	-0.3123151751	1.2520378742
H	-6.1853451196	-3.1157078279	-0.2196392522
H	-6.9310797697	-2.4840956674	-2.5031864561
H	1.4213813202	-3.2822434583	-0.0554207104
H	5.3808253622	0.8203402951	-1.4250476130
H	3.4933412615	-4.3616607839	0.8458907027
H	5.3717472576	3.2325059850	-1.9833202291
H	5.5536096986	-2.9994534579	1.1380152599
C	-1.0043040709	2.6890415819	2.1539822993
H	-0.1191811517	3.3302206672	2.2401718796
H	-0.7402355218	1.7358367968	2.6294964900
H	-1.1846021546	2.4983242127	1.0908902004
C	-4.5205242907	4.2886322230	2.7850871677
H	-4.3546527291	4.4746628022	3.8505636879
H	-4.7071071186	5.2529284572	2.2985662679
H	-5.4391875804	3.7004146411	2.6773099217
C	-4.9772523063	0.7904456016	2.2902521783
H	-5.1532643414	1.4350132104	1.4257527897
H	-5.6719990691	-0.0556622105	2.2273516814
H	-5.1816227454	1.3342390756	3.2161136295
C	-2.9401201295	-0.0607542599	3.6093527930
H	-1.9341447697	-0.4647073790	3.4782915773
H	-2.8886107323	0.8417531945	4.2296675208
H	-3.5571388603	-0.7921895546	4.1419799200

12-cis

0 1

C	-2.7100209275	0.6311687305	2.7846143850
N	-3.0295889956	-0.3397026290	1.6943655897
N	-3.0679972167	0.3985258285	0.4360841532
H	-2.0991603531	0.3012520702	-0.0705369518
O	-0.7376016952	0.3285719887	-0.6812356888
C	-3.1638797390	1.8633517564	0.7882420546
C	-2.2827343168	1.9089321134	2.0304146499
O	-0.4586370881	-1.5510457597	1.0688504182
P	0.1662377998	-0.5523612942	0.1607903479
O	1.1308453442	0.4072043977	1.0821726716
O	1.2540842028	-1.1806724593	-0.8718790558
C	2.0512520727	1.2320283048	0.4731484903
C	2.4532038954	-1.6687954763	-0.3940778551
C	3.2329783238	0.6874283076	-0.0480846151
C	1.8213595696	2.6027711336	0.4651043436
C	3.4665629497	-0.7737136022	-0.0272519663
C	2.6538851590	-3.0431670113	-0.3747122040
C	4.1856531720	1.5698247421	-0.5672170175
C	2.7825821087	3.4605545254	-0.0579311734
H	0.8912585183	2.9752617600	0.8830692381
C	4.7044027535	-1.3068738390	0.3462369033
C	3.8924187221	-3.5492723585	0.0033917474
C	3.9693684617	2.9432392932	-0.5726652811
H	2.6044092153	4.5312903850	-0.0640479720
C	4.9214317605	-2.6799253565	0.3600082491
H	5.4950443386	-0.6257745184	0.6487186698
C	-4.1111421140	-0.0739522758	-0.4670256381
C	-5.3796473825	-0.3640537380	0.0200778419
C	-3.7922427897	-0.1870912896	-1.8143972070
C	-6.3617712556	-0.7625959623	-0.8794864069

H	-5.5689575378	-0.3080222030	1.0871314451
C	-4.7875131966	-0.5879046570	-2.7037844641
H	-2.7773164760	0.0194325023	-2.1451295785
C	-6.0683201630	-0.8707338116	-2.2390603040
H	-4.5543788600	-0.6867830910	-3.7587733633
H	-2.2427851426	-0.9951404255	1.5755392827
H	-7.3570948986	-0.9990642556	-0.5175550239
H	-6.8391123835	-1.1872928256	-2.9344018170
H	1.8320364509	-3.6890478231	-0.6634272633
H	5.0990710877	1.1595016173	-0.9887566151
H	4.0526780430	-4.6226009314	0.0206289753
H	4.7208932650	3.6075812842	-0.9866800844
H	5.8881121395	-3.0712792283	0.6599531421
C	-1.5875659127	0.0736337954	3.6563619632
H	-1.3650314461	0.7743399852	4.4675232904
H	-1.8919026244	-0.8764260876	4.1076236283
H	-0.6809827000	-0.1014220726	3.0712299694
C	-3.9729625557	0.8340372157	3.6266055513
H	-4.7922192716	1.2707869622	3.0444196074
H	-4.3110950239	-0.1366661496	3.9992239866
H	-3.7767717557	1.4853644494	4.4841401435
C	-2.3863041056	3.2124964860	2.8089047385
H	-1.9655635090	4.0432472113	2.2344771369
H	-3.4268983959	3.4568708980	3.0461674809
H	-1.8296732090	3.1464991751	3.7487193581
C	-2.7393080715	2.7392519348	-0.3755400682
H	-3.3987911224	2.5991301636	-1.2368783877
H	-2.8047228382	3.7878515761	-0.0717785874
H	-1.7118425150	2.5101020846	-0.6725142074
H	-1.2439553337	1.7611623971	1.6971766377
H	-4.2118926515	2.0501105966	1.0534165562

12-trans

0 1

C	0	2.9462517535	2.0889082247	-1.4569611792
N	0	2.7971122281	0.6073778709	-1.3419806916
N	0	2.5652121466	0.3527104706	0.0630545650
H	0	1.5757554232	0.7211151648	0.3505459216
O	0	0.1401341423	1.0860278749	0.6786753407
C	0	3.5823464918	1.2194551090	0.7570415746
C	0	3.4315718571	2.5355832398	-0.0306449159
H	0	4.4218640105	2.9937932798	-0.1293732471
H	0	3.2354146896	1.3217439386	1.7904380607
O	0	0.0597310200	-0.2003200550	-1.5518176048
P	0	-0.6532122016	0.2823585597	-0.3376704934
O	0	-1.9795767782	1.1087396979	-0.8011032104
O	0	-1.3320680404	-0.9269259013	0.5293741050
C	0	-3.0057125164	1.3456345394	0.0884723182
C	0	-2.3770136985	-1.6402894518	-0.0187270691
C	0	-3.8796879592	0.3079829151	0.4417353224
C	0	-3.2010320234	2.6443216609	0.5424490936
C	0	-3.6580014735	-1.0736420311	-0.0400096142
C	0	-2.1446419907	-2.9359539307	-0.4626673759
C	0	-4.9715985590	0.6245488350	1.2565983191
C	0	-4.2926140054	2.9335493170	1.3532979786
H	0	-2.4915342961	3.4069214637	0.2401988507
C	0	-4.7125179838	-1.8636058573	-0.5090353612
C	0	-3.2090564205	-3.7007986386	-0.9266773330
C	0	-5.1832603970	1.9223155770	1.7077145925
H	0	-4.4468400672	3.9478104382	1.7077971205

C	0	-4.4958181441	-3.1654915927	-0.9460741052
H	0	-5.7095523044	-1.4334836557	-0.5469609692
C	0	2.5866598646	-1.0725703255	0.3914064906
C	0	3.3715039761	-1.9583904997	-0.3370377109
C	0	1.7953310893	-1.4908366465	1.4553124614
C	0	3.3720954007	-3.3006478959	0.0282813581
H	0	3.9456270599	-1.5950310837	-1.1812356749
C	0	1.8065701794	-2.8381455319	1.8065030361
H	0	1.1532325446	-0.7807872229	1.9688282073
C	0	2.5954459605	-3.7408090219	1.0986944987
H	0	1.1834278490	-3.1797512176	2.6262426643
H	0	1.9210360129	0.2922326071	-1.7864448528
H	0	3.9763064641	-4.0063568704	-0.5325578201
H	0	2.5965978442	-4.7910172019	1.3725628600
H	0	-1.1294170298	-3.3172479804	-0.4299171972
H	0	-5.6483115362	-0.1727725407	1.5513046209
H	0	-3.0331482126	-4.7132391405	-1.2762967955
H	0	-6.0349767502	2.1417245627	2.3434063374
H	0	-5.3279138736	-3.7576271490	-1.3131271565
C	0	4.9774146515	0.6202216661	0.7123151986
H	0	5.6744187321	1.3305688807	1.1652696801
H	0	5.0346164788	-0.3187134015	1.2678160603
H	0	5.2897448280	0.4388982931	-0.3199516295
C	0	2.5118044745	3.5123461407	0.7052625795
H	0	2.9714982244	3.8013576520	1.6558115215
H	0	2.3509910834	4.4239428149	0.1239685291
H	0	1.5370742053	3.0612487587	0.9188640430
C	0	1.6287989735	2.7391005909	-1.8944372824
H	0	1.7673539079	3.8109053712	-2.0669968455
H	0	1.2925670545	2.2860210965	-2.8322649826
H	0	0.8386937151	2.6018278743	-1.1532058641

C	0	4.0223940674	2.3520471833	-2.5099060692
H	0	4.1888429488	3.4282065201	-2.6241439451
H	0	4.9665106477	1.8780331467	-2.2258177636
H	0	3.7144672611	1.9481525082	-3.4793585502

TS2

0 1

C	2.7688086752	-1.5444013951	-1.9162760440
N	2.4426530862	-1.4998849826	-0.6363539531
N	2.8248371740	-0.4201190821	0.0799806686
H	2.1022946605	-0.1844955418	0.7944238110
O	0.4594458493	0.2140629094	1.2017326459
C	2.0956028516	1.1151544891	-1.5933547059
C	1.8020829675	0.2463401942	-2.6098190261
H	2.3947829392	0.2522584624	-3.5212276801
H	1.3596422369	1.3561664659	-0.8318337675
O	-0.0901709860	-1.8149419020	-0.3012389641
P	-0.5454759313	-0.5660363601	0.4125536061
O	-1.2342515932	0.3615871441	-0.7530009913
O	-1.8204490839	-0.8584728797	1.3753843795
C	-2.0919779864	1.3852862739	-0.4114548736
C	-3.0205677464	-1.2566690796	0.8224929513
C	-3.3916913765	1.0901407046	0.0229724865
C	-1.6720485201	2.6968461963	-0.5982685927
C	-3.8385649754	-0.3103306401	0.1928473965
C	-3.4240387887	-2.5759884282	0.9844475118
C	-4.2586191944	2.1610315963	0.2635489176
C	-2.5519802270	3.7447214417	-0.3522372168
H	-0.6606481700	2.8714781900	-0.9496985069
C	-5.0938259065	-0.7313411949	-0.2586571713

C	-4.6754111427	-2.9709033555	0.5252550709
H	-2.7471453053	-3.2658859592	1.4761798455
C	-3.8492190159	3.4766120590	0.0791113312
H	-2.2237949385	4.7691687848	-0.4969138223
C	-5.5141391328	-2.0460240466	-0.0937390019
H	-4.9944075985	-4.0007933240	0.6502938110
H	-4.5383029415	4.2906855358	0.2790305888
H	-5.2628493685	1.9467334029	0.6184395457
H	-5.7334005880	-0.0128132153	-0.7637356296
H	-6.4899886748	-2.3510962296	-0.4574677987
C	4.1837727082	-0.1461531283	0.3652536345
C	5.2140835829	-1.0402902689	0.0773612649
C	4.4639556899	1.0799190274	0.9747475806
C	6.5263397041	-0.6927856481	0.3879791614
H	4.9909587547	-2.0092676516	-0.3568090630
C	5.7759655326	1.4077935246	1.2885655465
H	3.6458567153	1.7587036068	1.2005134413
C	6.8153671405	0.5278643883	0.9893534057
H	7.3253944988	-1.3931825650	0.1661369208
H	5.9871145990	2.3587008287	1.7671502936
H	7.8401597236	0.7900494051	1.2294910436
H	1.4273720455	-1.7991513055	-0.4075411381
H	0.7968928035	-0.1621805756	-2.6797410065
H	3.0118003457	1.7005117423	-1.5971600228
H	3.7489607697	-1.1967877969	-2.2224176931
H	2.2929731379	-2.3291588171	-2.4946541917

TS3a

0 1

C	0	-2.8815100000	-1.0579200000	-1.6020120000
N	0	-2.2888340000	0.0066540000	-1.0169160000
N	0	-2.5449590000	0.1937760000	0.3241830000
H	0	-1.6268670000	0.2893460000	0.8196780000
H	0	-1.2969340000	0.2380450000	-1.2724240000
O	0	0.0359810000	0.0520360000	1.1329050000
C	0	-3.1010800000	-2.1396110000	0.5370460000
C	0	-2.3821090000	-2.6277470000	-0.5331740000
H	0	-2.9911210000	-3.2041110000	-1.2339280000
H	0	-2.7182160000	-1.9853760000	1.5401850000
O	0	0.3149370000	0.6950360000	-1.3566480000
P	0	0.9323520000	0.3646000000	-0.0286410000
O	0	1.9593470000	-0.8867650000	-0.3111150000
O	0	1.9857130000	1.5101140000	0.4585250000
C	0	2.9291950000	-1.1961690000	0.6158530000
C	0	3.1491220000	1.7211960000	-0.2492460000
C	0	4.0777510000	-0.3982160000	0.7110890000
C	0	2.7840150000	-2.3488980000	1.3785770000
C	0	4.2178550000	0.8256120000	-0.1086760000
C	0	3.2634860000	2.8723950000	-1.0189440000
C	0	5.0866140000	-0.8064560000	1.5893000000
C	0	3.7995870000	-2.7336980000	2.2465560000
H	0	1.8732000000	-2.9282650000	1.2700010000
C	0	5.4185610000	1.1343280000	-0.7561200000
C	0	4.4665880000	3.1564490000	-1.6551660000
H	0	2.4029000000	3.5276610000	-1.0966350000
C	0	4.9558320000	-1.9630870000	2.3496300000
H	0	3.6870010000	-3.6334940000	2.8432460000
C	0	5.5483040000	2.2883930000	-1.5202470000
H	0	4.5584080000	4.0552710000	-2.2567760000
H	0	5.7492760000	-2.2560250000	3.0295850000

H	0	5.9750240000	-0.1882130000	1.6843850000
H	0	6.2504280000	0.4408060000	-0.6685070000
H	0	6.4879510000	2.5042300000	-2.0185010000
C	0	-3.5551930000	1.0949610000	0.7292500000
C	0	-4.3181460000	1.8356820000	-0.1741870000
C	0	-3.7974060000	1.2031130000	2.1035670000
C	0	-5.3322170000	2.6641530000	0.3008020000
H	0	-4.0862800000	1.7856230000	-1.2328660000
C	0	-4.8047660000	2.0406410000	2.5634200000
H	0	-3.1850850000	0.6322270000	2.7966340000
C	0	-5.5840620000	2.7692580000	1.6649150000
H	0	-5.9180030000	3.2432580000	-0.4064470000
H	0	-4.9825830000	2.1239350000	3.6309960000
H	0	-6.3721000000	3.4205730000	2.0276930000
C	0	-4.5180600000	-1.7819510000	0.1376420000
H	0	-4.9204950000	-1.0070700000	0.7933210000
H	0	-5.1936910000	-2.6431290000	0.2010480000
C	0	-4.3797540000	-1.2754830000	-1.3336210000
H	0	-4.9481150000	-0.3566480000	-1.5057830000
H	0	-4.7444290000	-2.0238440000	-2.0429300000
H	0	-1.3352162200	-2.6688334000	-0.7504927000
H	0	-2.3377819500	-1.3967160700	-2.4590283500

TS3b

0 1

C	0	2.7958850000	-1.1892180000	1.3361120000
N	0	2.1941510000	-0.0322940000	0.9499450000
N	0	2.4080560000	0.3778600000	-0.3524900000
H	0	1.4747700000	0.4752230000	-0.8149890000

O	0	-0.1697600000	0.1760930000	-1.2257470000
C	0	2.7178420000	-1.9374940000	-1.0981590000
C	0	2.2119910000	-2.5471320000	0.0397650000
H	0	2.8621690000	-3.2999120000	0.4946710000
H	0	2.0360610000	-1.5378240000	-1.8442270000
O	0	-0.3944980000	0.8231750000	1.2682510000
P	0	-1.0398170000	0.4785370000	-0.0396940000
O	0	-2.0372110000	-0.7943380000	0.2715050000
O	0	-2.1282990000	1.5990430000	-0.5080080000
C	0	-3.0331800000	-1.1222750000	-0.6196000000
C	0	-3.2710960000	1.7979090000	0.2354780000
C	0	-4.1964400000	-0.3418980000	-0.6815990000
C	0	-2.8999970000	-2.2807470000	-1.3762640000
C	0	-4.3293520000	0.8849390000	0.1348820000
C	0	-3.3785920000	2.9522220000	1.0015560000
C	0	-5.2285230000	-0.7718280000	-1.5216770000
C	0	-3.9388790000	-2.6874580000	-2.2057980000
H	0	-1.9781180000	-2.8472620000	-1.2943700000
C	0	-5.5133710000	1.1789750000	0.8189800000
C	0	-4.5648690000	3.2215460000	1.6745580000
H	0	-2.5261820000	3.6210010000	1.0477720000
C	0	-5.1081240000	-1.9331840000	-2.2765160000
H	0	-3.8344880000	-3.5916050000	-2.7974450000
C	0	-5.6365550000	2.3358080000	1.5799510000
H	0	-4.6514140000	4.1225940000	2.2736130000
H	0	-5.9202210000	-2.2428170000	-2.9263620000
H	0	-6.1283900000	-0.1668610000	-1.5909620000
H	0	-6.3366020000	0.4720230000	0.7626930000
H	0	-6.5628490000	2.5401150000	2.1072260000
C	0	3.2659110000	1.4822320000	-0.5830370000
C	0	3.9665450000	2.1205620000	0.4412090000

C	0	3.4261230000	1.9001950000	-1.9076350000
C	0	4.8400550000	3.1594720000	0.1303430000
H	0	3.8037630000	1.8129260000	1.4686500000
C	0	4.2921240000	2.9453640000	-2.2017450000
H	0	2.8637780000	1.4016790000	-2.6928430000
C	0	5.0107110000	3.5757940000	-1.1865570000
H	0	5.3803570000	3.6552420000	0.9311030000
H	0	4.4066810000	3.2686020000	-3.2317350000
H	0	5.6884450000	4.3900270000	-1.4201910000
C	0	4.1944780000	-1.9384570000	-1.3294690000
H	0	4.4459870000	-1.4992940000	-2.2976520000
H	0	4.5402840000	-2.9799390000	-1.3398250000
C	0	4.3111770000	-1.3235190000	1.1822990000
H	0	4.7495050000	-0.5677940000	1.8489580000
H	0	4.5830410000	-2.2993260000	1.5996280000
C	0	4.9370050000	-1.1678470000	-0.2076890000
H	0	4.9895420000	-0.1150100000	-0.4805600000
H	0	5.9698050000	-1.5259620000	-0.1552060000
H	0	1.2231400000	0.1723100000	1.2620310000
H	0	1.1614211800	-2.7034170600	0.1692950300
H	0	2.4124907500	-1.5654668300	2.2615014700

TS3c

0 1

C	0	2.6170270000	0.8378210000	-1.0355050000
N	0	1.9388710000	-0.3281510000	-0.9132720000
N	0	2.0707840000	-1.0746000000	0.2329520000
H	0	1.1117100000	-1.2454350000	0.6110070000
O	0	-0.5006960000	-0.8549700000	1.0601850000

C	0	2.2007790000	0.8260580000	1.6717960000
C	0	1.9923970000	1.8102020000	0.7235760000
H	0	2.7996450000	2.5295430000	0.6049000000
H	0	1.3184570000	0.3487340000	2.0976060000
O	0	-0.7427530000	-0.1952810000	-1.4300520000
P	0	-1.3793770000	-0.5044580000	-0.1053180000
O	0	-2.3438360000	0.7739040000	0.2495940000
O	0	-2.4953840000	-1.6894210000	-0.2389350000
C	0	-3.3553230000	0.6464450000	1.1747690000
C	0	-3.6236210000	-1.4755420000	-0.9999000000
C	0	-4.5335780000	-0.0319900000	0.8318600000
C	0	-3.2191280000	1.2796430000	2.4047160000
C	0	-4.6689730000	-0.7001040000	-0.4811650000
C	0	-3.7318660000	-2.1077280000	-2.2325210000
C	0	-5.5776900000	-0.0411850000	1.7621720000
C	0	-4.2703170000	1.2555580000	3.3142570000
H	0	-2.2870550000	1.7904210000	2.6223980000
C	0	-5.8415410000	-0.5935030000	-1.2360310000
C	0	-4.9064400000	-1.9850880000	-2.9661820000
H	0	-2.8892310000	-2.6900320000	-2.5886890000
C	0	-5.4546800000	0.5966210000	2.9912940000
H	0	-4.1636040000	1.7501400000	4.2746540000
C	0	-5.9656560000	-1.2303280000	-2.4654890000
H	0	-4.9937160000	-2.4780800000	-3.9293150000
H	0	-6.2766160000	0.5702380000	3.6993940000
H	0	-6.4895040000	-0.5776230000	1.5144390000
H	0	-6.6542340000	0.0174500000	-0.8527170000
H	0	-6.8825450000	-1.1285810000	-3.0371220000
C	0	2.9069860000	-2.2145860000	0.2283550000
C	0	2.9589170000	-2.9750670000	1.4008240000
C	0	3.6796290000	-2.5794590000	-0.8756510000

C	0	3.7944910000	-4.0831840000	1.4688360000
H	0	2.3322820000	-2.6945370000	2.2436660000
C	0	4.5189040000	-3.6862990000	-0.7901020000
H	0	3.6029450000	-2.0038690000	-1.7923740000
C	0	4.5853460000	-4.4405510000	0.3782670000
H	0	3.8254360000	-4.6713940000	2.3805940000
H	0	5.1159500000	-3.9667320000	-1.6524740000
H	0	5.2378110000	-5.3053160000	0.4352640000
C	0	4.1297690000	0.9253580000	-0.9059020000
H	0	4.5018360000	0.4506940000	-1.8282840000
H	0	4.3806500000	1.9874140000	-1.0038340000
C	0	4.7418600000	1.0564220000	1.6268150000
H	0	4.7162000000	2.1476820000	1.5050880000
H	0	5.6142410000	0.8443010000	2.2539360000
C	0	3.5017780000	0.5624900000	2.3640900000
H	0	3.4420230000	1.0264550000	3.3598970000
H	0	3.6002470000	-0.5157260000	2.5452830000
C	0	4.9457730000	0.3738970000	0.2724490000
H	0	4.8111070000	-0.7039110000	0.3860180000
H	0	5.9913510000	0.5135950000	-0.0260650000
H	0	0.9433910000	-0.2912910000	-1.2362350000
H	0	2.2239699600	1.3893829000	-1.8638682300
H	0	0.9982610000	2.1645325700	0.5473844300

TS4a

0 1

C	0	-2.8815100000	-1.0579200000	-1.6020120000
N	0	-2.2888340000	0.0066540000	-1.0169160000
N	0	-2.5449590000	0.1937760000	0.3241830000

H	0	-1.6268670000	0.2893460000	0.8196780000
H	0	-1.2969340000	0.2380450000	-1.2724240000
O	0	0.0359810000	0.0520360000	1.1329050000
C	0	-3.1010800000	-2.1396110000	0.5370460000
C	0	-2.3821090000	-2.6277470000	-0.5331740000
H	0	-2.9911210000	-3.2041110000	-1.2339280000
H	0	-2.7182160000	-1.9853760000	1.5401850000
O	0	0.3149370000	0.6950360000	-1.3566480000
P	0	0.9323520000	0.3646000000	-0.0286410000
O	0	1.9593470000	-0.8867650000	-0.3111150000
O	0	1.9857130000	1.5101140000	0.4585250000
C	0	2.9291950000	-1.1961690000	0.6158530000
C	0	3.1491220000	1.7211960000	-0.2492460000
C	0	4.0777510000	-0.3982160000	0.7110890000
C	0	2.7840150000	-2.3488980000	1.3785770000
C	0	4.2178550000	0.8256120000	-0.1086760000
C	0	3.2634860000	2.8723950000	-1.0189440000
C	0	5.0866140000	-0.8064560000	1.5893000000
C	0	3.7995870000	-2.7336980000	2.2465560000
H	0	1.8732000000	-2.9282650000	1.2700010000
C	0	5.4185610000	1.1343280000	-0.7561200000
C	0	4.4665880000	3.1564490000	-1.6551660000
H	0	2.4029000000	3.5276610000	-1.0966350000
C	0	4.9558320000	-1.9630870000	2.3496300000
H	0	3.6870010000	-3.6334940000	2.8432460000
C	0	5.5483040000	2.2883930000	-1.5202470000
H	0	4.5584080000	4.0552710000	-2.2567760000
H	0	5.7492760000	-2.2560250000	3.0295850000
H	0	5.9750240000	-0.1882130000	1.6843850000
H	0	6.2504280000	0.4408060000	-0.6685070000
H	0	6.4879510000	2.5042300000	-2.0185010000

C	0	-3.5551930000	1.0949610000	0.7292500000
C	0	-4.3181460000	1.8356820000	-0.1741870000
C	0	-3.7974060000	1.2031130000	2.1035670000
C	0	-5.3322170000	2.6641530000	0.3008020000
H	0	-4.0862800000	1.7856230000	-1.2328660000
C	0	-4.8047660000	2.0406410000	2.5634200000
H	0	-3.1850850000	0.6322270000	2.7966340000
C	0	-5.5840620000	2.7692580000	1.6649150000
H	0	-5.9180030000	3.2432580000	-0.4064470000
H	0	-4.9825830000	2.1239350000	3.6309960000
H	0	-6.3721000000	3.4205730000	2.0276930000
C	0	-2.1107810000	-1.5381600000	-2.8168240000
H	0	-2.7190250000	-2.2658410000	-3.3610900000
H	0	-1.9024470000	-0.7062300000	-3.4966380000
C	0	-0.9164070000	-2.6852700000	-0.8374310000
H	0	-0.3654370000	-2.0478990000	-0.1406620000
H	0	-0.5155780000	-3.6991890000	-0.7457390000
C	0	-0.7723760000	-2.1740030000	-2.3107040000
H	0	-0.5021260000	-2.9940410000	-2.9810030000
H	0	0.0295310000	-1.4340350000	-2.3551970000
H	0	-4.1018401100	-1.8870090800	0.2549618500
H	0	-3.9241471000	-1.2093234100	-1.4152370700

TS4b

0 1

C	0	2.7958850000	-1.1892180000	1.3361120000
N	0	2.1941510000	-0.0322940000	0.9499450000
N	0	2.4080560000	0.3778600000	-0.3524900000
H	0	1.4747700000	0.4752230000	-0.8149890000

O	0	-0.1697600000	0.1760930000	-1.2257470000
C	0	2.7178420000	-1.9374940000	-1.0981590000
C	0	2.2119910000	-2.5471320000	0.0397650000
H	0	2.8621690000	-3.2999120000	0.4946710000
H	0	2.0360610000	-1.5378240000	-1.8442270000
O	0	-0.3944980000	0.8231750000	1.2682510000
P	0	-1.0398170000	0.4785370000	-0.0396940000
O	0	-2.0372110000	-0.7943380000	0.2715050000
O	0	-2.1282990000	1.5990430000	-0.5080080000
C	0	-3.0331800000	-1.1222750000	-0.6196000000
C	0	-3.2710960000	1.7979090000	0.2354780000
C	0	-4.1964400000	-0.3418980000	-0.6815990000
C	0	-2.8999970000	-2.2807470000	-1.3762640000
C	0	-4.3293520000	0.8849390000	0.1348820000
C	0	-3.3785920000	2.9522220000	1.0015560000
C	0	-5.2285230000	-0.7718280000	-1.5216770000
C	0	-3.9388790000	-2.6874580000	-2.2057980000
H	0	-1.9781180000	-2.8472620000	-1.2943700000
C	0	-5.5133710000	1.1789750000	0.8189800000
C	0	-4.5648690000	3.2215460000	1.6745580000
H	0	-2.5261820000	3.6210010000	1.0477720000
C	0	-5.1081240000	-1.9331840000	-2.2765160000
H	0	-3.8344880000	-3.5916050000	-2.7974450000
C	0	-5.6365550000	2.3358080000	1.5799510000
H	0	-4.6514140000	4.1225940000	2.2736130000
H	0	-5.9202210000	-2.2428170000	-2.9263620000
H	0	-6.1283900000	-0.1668610000	-1.5909620000
H	0	-6.3366020000	0.4720230000	0.7626930000
H	0	-6.5628490000	2.5401150000	2.1072260000
C	0	3.2659110000	1.4822320000	-0.5830370000
C	0	3.9665450000	2.1205620000	0.4412090000

C	0	3.4261230000	1.9001950000	-1.9076350000
C	0	4.8400550000	3.1594720000	0.1303430000
H	0	3.8037630000	1.8129260000	1.4686500000
C	0	4.2921240000	2.9453640000	-2.2017450000
H	0	2.8637780000	1.4016790000	-2.6928430000
C	0	5.0107110000	3.5757940000	-1.1865570000
H	0	5.3803570000	3.6552420000	0.9311030000
H	0	4.4066810000	3.2686020000	-3.2317350000
H	0	5.6884450000	4.3900270000	-1.4201910000
C	0	2.2501510000	-1.7247810000	2.6533370000
H	0	2.7876780000	-2.6499630000	2.8914470000
H	0	2.5140550000	-0.9975660000	3.4319500000
C	0	0.7278840000	-2.7679110000	0.2227480000
H	0	0.1916900000	-1.8896960000	-0.1494170000
H	0	0.4247950000	-3.6064950000	-0.4182650000
C	0	0.3356360000	-3.0817970000	1.6676270000
H	0	-0.7486110000	-3.2234770000	1.7125550000
H	0	0.7981880000	-4.0342480000	1.9663480000
C	0	0.7386170000	-1.9910070000	2.6599710000
H	0	0.4601830000	-2.3007030000	3.6727070000
H	0	0.1739720000	-1.0755960000	2.4494860000
H	0	1.2231400000	0.1723100000	1.2620310000
H	0	3.8563002400	-1.2832030700	1.2284722500
H	0	3.7749506300	-1.9381834000	-1.2637514700

TS4c

0 1

C	0	2.8330400000	1.0032550000	-1.0347430000
N	0	2.1363310000	-0.1537190000	-0.9159440000

N	0	2.2604730000	-0.8411170000	0.2700320000
H	0	1.3069360000	-0.9912570000	0.6715870000
H	0	1.1468560000	-0.1433340000	-1.2542660000
O	0	-0.2883770000	-0.5888790000	1.1157710000
C	0	2.5025390000	1.2103250000	1.5340360000
C	0	2.1383940000	2.0990790000	0.5470190000
H	0	2.8984420000	2.8362580000	0.2919370000
H	0	1.7435350000	0.7231080000	2.1424210000
O	0	-0.5474980000	-0.1533250000	-1.4228910000
P	0	-1.1752620000	-0.3851870000	-0.0778700000
O	0	-2.2075180000	0.8654620000	0.1681500000
O	0	-2.2274510000	-1.6339540000	-0.1187940000
C	0	-3.2142020000	0.7583040000	1.1012510000
C	0	-3.3604280000	-1.5403450000	-0.8967290000
C	0	-4.3541310000	-0.0053410000	0.8125100000
C	0	-3.1150620000	1.4895330000	2.2792460000
C	0	-4.4476980000	-0.7816290000	-0.4433600000
C	0	-3.4285780000	-2.2733390000	-2.0752500000
C	0	-5.4001990000	0.0032310000	1.7405660000
C	0	-4.1672260000	1.4812390000	3.1879600000
H	0	-2.2104390000	2.0609850000	2.4574100000
C	0	-5.6198560000	-0.7970840000	-1.2060270000
C	0	-4.6036830000	-2.2711040000	-2.8181590000
H	0	-2.5545260000	-2.8368690000	-2.3825720000
C	0	-5.3150850000	0.7396490000	2.9166410000
H	0	-4.0894570000	2.0522900000	4.1078780000
C	0	-5.7036470000	-1.5355310000	-2.3808680000
H	0	-4.6596890000	-2.8429720000	-3.7391020000
H	0	-6.1373340000	0.7255500000	3.6247510000
H	0	-6.2818220000	-0.5977800000	1.5354210000
H	0	-6.4654640000	-0.2010640000	-0.8737970000

H	0	-6.6212640000	-1.5277900000	-2.9602750000
C	0	3.1518700000	-1.9282510000	0.3723500000
C	0	3.9119700000	-2.3971900000	-0.7013670000
C	0	3.2655010000	-2.5423940000	1.6257910000
C	0	4.7988750000	-3.4533820000	-0.5085580000
H	0	3.7740310000	-1.9543190000	-1.6825450000
C	0	4.1447240000	-3.6027510000	1.8006540000
H	0	2.6508010000	-2.1836860000	2.4476570000
C	0	4.9243440000	-4.0585310000	0.7379540000
H	0	5.3829300000	-3.8146850000	-1.3494810000
H	0	4.2214580000	-4.0757850000	2.7746580000
H	0	5.6127080000	-4.8849550000	0.8791060000
C	0	2.4025340000	1.7771670000	-2.2717890000
H	0	3.0485970000	1.4094190000	-3.0797640000
H	0	1.3809090000	1.4729440000	-2.5308540000
C	0	2.4743270000	3.3151390000	-2.2260220000
H	0	3.3053130000	3.6548190000	-1.5925800000
H	0	2.7211020000	3.6554320000	-3.2368990000
C	0	1.1527200000	4.0000340000	-1.8316420000
H	0	0.3580140000	3.5535980000	-2.4433530000
H	0	1.2141450000	5.0529510000	-2.1306160000
C	0	0.7247080000	2.5363350000	0.2424080000
H	0	0.2398970000	1.8444780000	-0.4563710000
H	0	0.1224920000	2.5010610000	1.1564380000
C	0	0.7223250000	3.9447120000	-0.3608700000
H	0	1.3732700000	4.5949290000	0.2402170000
H	0	-0.2853180000	4.3672470000	-0.2920440000
H	0	3.5401421100	1.0587473400	1.7468833800
H	0	3.8797720700	0.9850951800	-0.8135594000

TS4d

0 1

C	0	2.6170270000	0.8378210000	-1.0355050000
N	0	1.9388710000	-0.3281510000	-0.9132720000
N	0	2.0707840000	-1.0746000000	0.2329520000
H	0	1.1117100000	-1.2454350000	0.6110070000
O	0	-0.5006960000	-0.8549700000	1.0601850000
C	0	2.2007790000	0.8260580000	1.6717960000
C	0	1.9923970000	1.8102020000	0.7235760000
H	0	2.7996450000	2.5295430000	0.6049000000
H	0	1.3184570000	0.3487340000	2.0976060000
O	0	-0.7427530000	-0.1952810000	-1.4300520000
P	0	-1.3793770000	-0.5044580000	-0.1053180000
O	0	-2.3438360000	0.7739040000	0.2495940000
O	0	-2.4953840000	-1.6894210000	-0.2389350000
C	0	-3.3553230000	0.6464450000	1.1747690000
C	0	-3.6236210000	-1.4755420000	-0.9999000000
C	0	-4.5335780000	-0.0319900000	0.8318600000
C	0	-3.2191280000	1.2796430000	2.4047160000
C	0	-4.6689730000	-0.7001040000	-0.4811650000
C	0	-3.7318660000	-2.1077280000	-2.2325210000
C	0	-5.5776900000	-0.0411850000	1.7621720000
C	0	-4.2703170000	1.2555580000	3.3142570000
H	0	-2.2870550000	1.7904210000	2.6223980000
C	0	-5.8415410000	-0.5935030000	-1.2360310000
C	0	-4.9064400000	-1.9850880000	-2.9661820000
H	0	-2.8892310000	-2.6900320000	-2.5886890000
C	0	-5.4546800000	0.5966210000	2.9912940000
H	0	-4.1636040000	1.7501400000	4.2746540000
C	0	-5.9656560000	-1.2303280000	-2.4654890000

H	0	-4.9937160000	-2.4780800000	-3.9293150000
H	0	-6.2766160000	0.5702380000	3.6993940000
H	0	-6.4895040000	-0.5776230000	1.5144390000
H	0	-6.6542340000	0.0174500000	-0.8527170000
H	0	-6.8825450000	-1.1285810000	-3.0371220000
C	0	2.9069860000	-2.2145860000	0.2283550000
C	0	2.9589170000	-2.9750670000	1.4008240000
C	0	3.6796290000	-2.5794590000	-0.8756510000
C	0	3.7944910000	-4.0831840000	1.4688360000
H	0	2.3322820000	-2.6945370000	2.2436660000
C	0	4.5189040000	-3.6862990000	-0.7901020000
H	0	3.6029450000	-2.0038690000	-1.7923740000
C	0	4.5853460000	-4.4405510000	0.3782670000
H	0	3.8254360000	-4.6713940000	2.3805940000
H	0	5.1159500000	-3.9667320000	-1.6524740000
H	0	5.2378110000	-5.3053160000	0.4352640000
C	0	0.5783430000	2.3142000000	0.4729620000
H	0	0.1035170000	1.7970510000	-0.3683290000
H	0	-0.0354760000	2.0432140000	1.3385010000
C	0	0.4881260000	3.8281660000	0.2533490000
H	0	1.0956180000	4.3429930000	1.0112420000
H	0	-0.5498950000	4.1287990000	0.4342950000
C	0	0.8823370000	4.3407220000	-1.1374970000
H	0	0.6442170000	5.4095080000	-1.1692510000
H	0	0.2323120000	3.8697840000	-1.8876080000
C	0	2.3579930000	4.1524200000	-1.5452330000
H	0	2.7163750000	5.0687550000	-2.0274820000
H	0	2.9866820000	4.0386080000	-0.6540590000
C	0	2.0559720000	1.6251280000	-2.2179220000
H	0	0.9641530000	1.6483690000	-2.1323370000
H	0	2.2548090000	0.9891820000	-3.0915670000

C	0	2.6060850000	3.0160540000	-2.5452460000
H	0	3.6767240000	2.9544050000	-2.7726070000
H	0	2.1244100000	3.3019110000	-3.4884180000
H	0	0.9433910000	-0.2912910000	-1.2362350000
H	0	3.6813540400	0.8994098200	-0.9443196000
H	0	3.1306166200	0.6376831900	2.1665857800

TS5-cis

0 1

C	-3.0193301988	-1.9304154134	0.4894059582
N	-2.2546907440	-1.1063736626	-0.2470492904
N	-2.4654108864	0.2421008055	-0.1608106702
H	-1.5448986753	0.7023702887	-0.3876127031
O	0.0814948295	0.9472340728	-0.7265016598
C	-2.5374562908	0.2593530616	2.1656307916
C	-2.6561848084	-1.0953793375	2.4340332336
O	0.3725794447	-1.4572712865	0.1639639952
P	0.9892304313	-0.1564973110	-0.2744896123
O	1.9436991931	0.3221972458	0.9680141927
O	2.1056041082	-0.3731991455	-1.4443974834
C	2.8983890076	1.2928878205	0.7579878035
C	3.2770998523	-1.0434100715	-1.1660495852
C	4.0898841779	0.9607123966	0.0994635641
C	2.6888457417	2.5631917179	1.2808074508
C	4.2946873509	-0.3972427765	-0.4508360233
C	3.4544516260	-2.3205227927	-1.6843892724
C	5.0773452305	1.9458551444	-0.0017844165
C	3.6842250382	3.5269889830	1.1659940328
H	1.7458417415	2.7678760903	1.7765353524

C	5.5083451327	-1.0725227762	-0.2866046336
C	4.6690972061	-2.9729010045	-1.5057287708
C	4.8830721911	3.2168994260	0.5268774650
H	3.5225870681	4.5201225307	1.5732310656
C	5.7004958956	-2.3463201271	-0.8091414318
H	6.2999462995	-0.5892985405	0.2794626171
C	-3.5501523457	0.8227437991	-0.8927923142
C	-4.1225411516	0.1959914387	-1.9992854731
C	-3.9961146891	2.0799623331	-0.4867762912
C	-5.1652768562	0.8207501426	-2.6766967308
H	-3.7336438952	-0.7629053115	-2.3267808603
C	-5.0268726461	2.7031253090	-1.1814268342
H	-3.5278453875	2.5600176127	0.3676931012
C	-5.6220231511	2.0722889115	-2.2717785314
H	-5.3693453496	3.6831384356	-0.8647771799
H	-1.2241621565	-1.3225696928	-0.2110423422
H	-5.6114678733	0.3305824080	-3.5363715908
H	-6.4311787824	2.5572292088	-2.8077105810
H	2.6327368735	-2.7744910212	-2.2273074798
H	5.9997719640	1.7065324042	-0.5236985641
H	4.8093891880	-3.9704118208	-1.9101677277
H	5.6607802833	3.9675574511	0.4300016403
H	6.6489591328	-2.8532449099	-0.6636338278
C	-4.5199238891	-1.7982846358	0.4283937249
H	-4.8630213198	-0.7842447154	0.6390137391
H	-4.8487917952	-2.0618280182	-0.5840175328
H	-4.9909260101	-2.4928251563	1.1242200787
C	-2.4672959254	-3.3278304679	0.5969673827
H	-1.3738050293	-3.3120531887	0.5953163092
H	-2.8228337180	-3.7984732709	1.5173430130
H	-2.8203386058	-3.9361028016	-0.2435919877

C	-1.2129698856	0.9608146695	2.2543342938
H	-1.0810134831	1.3140220891	3.2860998215
H	-0.3959460257	0.2650394467	2.0456709599
H	-1.1204426990	1.8203025417	1.5867837742
C	-3.8111446223	-1.6231445735	3.2589895599
H	-3.5795167977	-1.5123193500	4.3236203147
H	-4.7334858691	-1.0675527004	3.0646059520
H	-4.0064140316	-2.6870626374	3.0876939338
H	-3.4356297739	0.8763698954	2.2340506330
H	-1.7090366394	-1.6202571625	2.5748206478

TS5-trans

0 1

C	-2.8819524611	0.0896331628	2.0552972140
N	-2.2846761851	-0.5619522809	1.0276038327
N	-2.5503088831	-0.1238588179	-0.2424062131
H	-1.6359761682	-0.0212420508	-0.7403190610
O	0.0088690190	0.4212697083	-0.8471419349
C	-2.6505131738	2.1615079815	0.3139305617
C	-2.3067628828	2.0342616607	1.6553186497
H	-3.0318099769	2.4277713915	2.3697486656
O	0.3847969295	-0.9029220231	1.3406733980
P	0.9524862002	-0.2226951683	0.1281582083
O	2.0496831417	0.8638348967	0.6733935945
O	1.9191217222	-1.2202664077	-0.7303023468
C	2.9833794187	1.3876712579	-0.1933403770
C	3.1064525488	-1.6600118861	-0.1865544362
C	4.0847317547	0.6118097816	-0.5798965475
C	2.8538117286	2.7117469932	-0.5951990989

C	4.2090372748	-0.7960356196	-0.1424919196
C	3.2056469854	-2.9836926349	0.2243296000
C	5.0640784195	1.2168053238	-1.3740069699
C	3.8393849214	3.2910220338	-1.3863465408
H	1.9805474778	3.2670434479	-0.2696788587
C	5.4262041904	-1.3116235936	0.3141957864
C	4.4258297148	-3.4728377467	0.6771120369
C	4.9494416304	2.5433298847	-1.7739086046
H	3.7395685685	4.3251579705	-1.7008689078
C	5.5400622619	-2.6369135262	0.7180962644
H	6.2850863491	-0.6483135884	0.3683543861
C	-3.5989017094	-0.6982141590	-0.9984847840
C	-4.4085586780	-1.7260684912	-0.5131418236
C	-3.8074973526	-0.1910303804	-2.2854733522
C	-5.4394405562	-2.2199393394	-1.3068915869
H	-4.2020053016	-2.1503882657	0.4633807219
C	-4.8348464110	-0.6997045373	-3.0705950161
H	-3.1530116103	0.5921868521	-2.6598889874
C	-5.6622167841	-1.7095944857	-2.5827881067
H	-4.9872971553	-0.3044635827	-4.0698929487
H	-1.2659506294	-0.7755285769	1.1713578088
H	-6.0624921528	-3.0235829198	-0.9266977364
H	-6.4645176211	-2.1038538860	-3.1973144115
H	2.3196461136	-3.6069432418	0.1735074208
H	5.9149375957	0.6216980657	-1.6939868899
H	4.5056252486	-4.5064929946	0.9987552492
H	5.7190078187	2.9892461772	-2.3956929013
H	6.4932220268	-3.0135296210	1.0750498774
C	-3.9924182293	2.6127108659	-0.1709566732
H	-3.8820650864	3.5652408977	-0.7025176856
H	-4.4336025064	1.9039890967	-0.8831683549

H	-4.6939289684	2.7662574843	0.6529120669
H	-1.8374377471	2.1811001625	-0.4097149034
C	-0.8459949066	2.1618624456	2.0386018631
H	-0.6566956026	3.1685990537	2.4270324926
H	-0.5169592889	1.4505111470	2.7991600753
H	-0.2116575245	2.0189707736	1.1615789469
C	-4.3848593464	0.2336727600	2.0387751294
H	-4.7685900156	0.5355919041	1.0647776640
H	-4.8262074456	-0.7394721241	2.2861089339
H	-4.7069464459	0.9501288804	2.7980800007
C	-2.2912039512	-0.2564851022	3.3998748792
H	-1.2273754528	-0.4923388931	3.3151354934
H	-2.4312155326	0.5726496946	4.0983305674
H	-2.8125003177	-1.1296168109	3.8074135894

References

[1] Frisch, M. J.; Trucks, G. W.; Schlegel, H. B.; Scuseria, G. E.; Robb, M. A.; Cheeseman, J. R.; Scalmani, G.; Barone, V.; Petersson, G. A.; Nakatsuji, H.; Li, X.; Caricato, M.; Marenich, A. V.; Bloino, J.; Janesko, B. G.; Gomperts, R.; Mennucci, B.; Hratchian, H. P.; Ortiz, J. V.; Izmaylov, A. F.; Sonnenberg, J. L.; Williams; Ding, F.; Lipparini, F.; Egidi, F.; Goings, J.; Peng, B.; Petrone, A.; Henderson, T.; Ranasinghe, D.; Zakrzewski, V. G.; Gao, J.; Rega, N.; Zheng, G.; Liang, W.; Hada, M.; Ehara, M.; Toyota, K.; Fukuda, R.; Hasegawa, J.; Ishida, M.; Nakajima, T.; Honda, Y.; Kitao, O.; Nakai, H.; Vreven, T.; Throssell, K.; Montgomery Jr., J. A.; Peralta, J. E.; Ogliaro, F.; Bearpark, M. J.; Heyd, J. J.; Brothers, E. N.; Kudin, K. N.; Staroverov, V. N.; Keith, T. A.; Kobayashi, R.; Normand, J.; Raghavachari, K.; Rendell, A. P.; Burant, J. C.; Iyengar, S. S.; Tomasi, J.; Cossi, M.; Millam, J. M.; Klene, M.; Adamo, C.; Cammi, R.; Ochterski, J. W.; Martin, R. L.; Morokuma, K.; Farkas, O.; Foresman, J. B.; Fox, D. J. Wallingford, CT, 2016.

[2] Walker, M.; Harvey, A. J. A.; Sen, A.; Dessent, C. E. H. *J. Phys. Chem. A* **2013**, *117*, 12590-12600.

[3] Blaudeau, J.-P.; McGrath, M. P.; Curtiss, L. A.; Radom, L. *J. Chem. Phys.* **1997**, *107*, 5016-5021.

[4] Hehre, W. J.; Ditchfield, R.; Pople, J. A. *J. Chem. Phys.* **1972**, *56*, 2257-2261.

[5] Marenich, A. V.; Cramer, C. J.; Truhlar, D. G. *J. Phys. Chem. B.* **2009**, *113*, 6378-6396.

[6] Fukui, K. *J. Phys. Chem.* **1970**, *74*, 4161-4163.

[7] Tanaka, R.; Yamashita, M.; Chung, L. W.; Morokuma, K.; Nozaki, K. *Organometallics* **2011**, *30*, 6742-6750.

[8] *Schrödinger Release 2016-1: MacroModel, version 11.1, Schrödinger, LLC, New York, NY, 2016.*

[9] Legault, C. Y. *Université de Sherbrooke* **2009**, http://www.cylview.org (visited Dec 1st, 2021).

[10] Savin, A.; Becke, A. D.; Flad, J.; Nesper, R.; Preuss, H.; von Schnering, H. G. *Angew. Chem. Int. Ed.* **1991**, *30*, 409-412.

[11] Silvi, B.; Savin, A. *Nature* **1994**, *371*, 683.

[12] Savin, A.; Nesper, R.; Wengert, S.; Fässler, T. F. *Angew. Chem. Int. Ed.* **1997**, *36*, 1808-1832.

[13] Noury, S.; Krokidis, X.; Fuster, F.; Silvi, B. *Comput. Chem.* **1999**, *23*, 597-604.

[14] Capel, E.; Rodriguez-Rodriguez, M.; Uria, U.; Pedron, M.; Tejero, T.; Vicario, J. L.; Merino, P. *J. Org. Chem.* **2022**, *87*, 693-707.

[15] Ortega, A.; Manzano, R.; Uria, U.; Carrillo, L.; Reyes, E.; Tejero, T.; Merino, P.; Vicario, J. L. *Angew. Chem. Int. Ed.* **2018**, *57*, 8225-8229.

[16] Roca-López, D.; Polo, V.; Tejero, T.; Merino, P. *Eur. J. Org. Chem.* **2015**, 4143-4152.

[17] Johnson, E. R.; Keinan, S.; Mori-Sanchez, P.; Contreras-Garcia, J.; Cohen, A. J.; Yang, W. *J. Am. Chem. Soc.* **2010**, *132*, 6498-6506.

[18] Boto, R. A.; Peccati, F.; Laplaza, R.; Quan, C.; Carbone, A.; Piquemal, J.-P.; Maday, Y.; Contreras-García, J. *J. Chem. Theory Comput.* **2020**, *16*, 4150-4158.

[19] Humphrey, W.; Dalke, A.; Schulten, K. *J. Mol. Graph.* **1996**, *14*, 33-38.
